# Age-related loss of Notch3 underlies brain vascular contractility deficiencies, glymphatic dysfunction, and neurodegeneration in mice

**DOI:** 10.1172/JCI166134

**Published:** 2024-01-16

**Authors:** Milagros C. Romay, Russell H. Knutsen, Feiyang Ma, Ana Mompeón, Gloria E. Hernandez, Jocelynda Salvador, Snezana Mirkov, Ayush Batra, David P. Sullivan, Daniele Procissi, Samuel Buchanan, Elise Kronquist, Elisa A. Ferrante, William A. Muller, Jordain Walshon, Alicia Steffens, Kathleen McCortney, Craig Horbinski, Elisabeth Tournier‑Lasserve, Adam M. Sonabend, Farzaneh A. Sorond, Michael M. Wang, Manfred Boehm, Beth A. Kozel, M. Luisa Iruela-Arispe

**Affiliations:** 1 Department of Cell and Development Biology, Feinberg School of Medicine, Northwestern University, Chicago, Illinois, USA.; 2 National Heart, Lung, and Blood Institute, NIH, Bethesda, Maryland, USA.; 3 Molecular Biology Institute, University of California, Los Angeles, Los Angeles, California, USA.; 4 Department of Pathology,; 5 Department of Neurology, and; 6 Department of Radiology, Feinberg School of Medicine, Northwestern University, Chicago, Illinois, USA.; 7 Department of Biomedical Engineering, Northwestern University, Evanston, Illinois, USA.; 8 Laboratory of Cardiovascular Regenerative Medicine, NIH, Bethesda, Maryland, USA.; 9 Department of Neurological Surgery, Feinberg School of Medicine, Northwestern University, Chicago, Illinois, USA.; 10 Inserm NeuroDiderot, Université Paris Cité, Paris, France.; 11 Service de Génétique Neurovasculaire, Assistance Publique–Hôpitaux de Paris, Hôpital Saint-Louis, Paris, France.; 12 Northwestern Medicine Malnati Brain Tumor Institute of the Lurie Comprehensive Cancer Center, Feinberg School of Medicine, Chicago, Illinois, USA.; 13 Department of Neurology, University of Michigan, Ann Arbor, Michigan, USA.; 14 VA Ann Arbor Healthcare System, Ann Arbor, Michigan, USA.

**Keywords:** Neuroscience, Vascular Biology, Cardiovascular disease, Dementia, Neurological disorders

## Abstract

Vascular aging affects multiple organ systems, including the brain, where it can lead to vascular dementia. However, a concrete understanding of how aging specifically affects the brain vasculature, along with molecular readouts, remains vastly incomplete. Here, we demonstrate that aging is associated with a marked decline in Notch3 signaling in both murine and human brain vessels. To clarify the consequences of Notch3 loss in the brain vasculature, we used single-cell transcriptomics and found that Notch3 inactivation alters regulation of calcium and contractile function and promotes a notable increase in extracellular matrix. These alterations adversely impact vascular reactivity, manifesting as dilation, tortuosity, microaneurysms, and decreased cerebral blood flow, as observed by MRI. Combined, these vascular impairments hinder glymphatic flow and result in buildup of glycosaminoglycans within the brain parenchyma. Remarkably, this phenomenon mirrors a key pathological feature found in brains of patients with CADASIL, a hereditary vascular dementia associated with *NOTCH3* missense mutations. Additionally, single-cell RNA sequencing of the neuronal compartment in aging *Notch3-*null mice unveiled patterns reminiscent of those observed in neurodegenerative diseases. These findings offer direct evidence that age-related NOTCH3 deficiencies trigger a progressive decline in vascular function, subsequently affecting glymphatic flow and culminating in neurodegeneration.

## Abstract 2

<p>Notch3 protects against small vessel disease, and age-related declines in Notch3 triggers vascular deficiencies, glymphatic dysfunction, and neurodegeneration in mice.</p>

## Graphical abstract

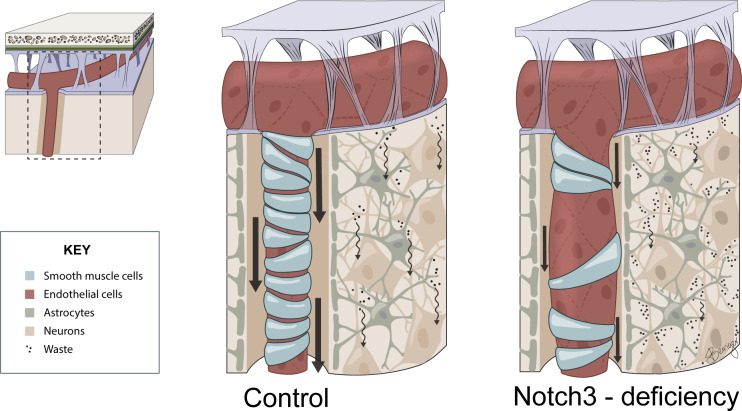

## Introduction

Aging introduces a series of complex molecular, structural, and functional changes to blood vessels, with important consequences for organ physiology (1). In the brain, compromised vascular function can trigger a spectrum of pathologies, ranging from acute incidents like strokes to enduring and incapacitating conditions such as cerebral hypoperfusion that can ultimately result in cognitive impairment and dementia (2–4). These vascular dysfunctions can be accelerated and intensified by concurrent medical conditions that negatively affect blood vessels, including diabetes, atherosclerosis, hypercholesterolemia, and hypertension (3). However, it is important to note that the presence of these conditions alone does not predict development of dementia, and their absence does not necessarily assure freedom from cognitive decline during the aging process. Consequently, we must inquire: What are the specific triggers and molecular markers associated with aging that can offer more robust predictions for the occurrence of cerebral hypoperfusion and subsequent cognitive decline? Answers to this question will contribute to the understanding, prevention, and treatment of cognitive deficiencies associated with aging and offered the foundation for this study.

At the core of vascular physiology resides cerebral blood flow (CBF) as the absolute readout of brain perfusion (5). The dynamic nature of CBF enables responses to various stimuli, such as increased brain activity and vasoactive challenges (6). Remarkably, cerebral autoregulation plays a pivotal role in maintaining CBF stability at a baseline level to ensure optimal brain perfusion. Notably, alterations in CBF have been observed in several neurodegenerative conditions, including Alzheimer’s disease, emphasizing the potential significance of CBF changes in the context of cognitive decline (7). The control of CBF lies within the purview of vascular smooth muscle cells (VSMCs) and pericytes, both contractile cells that finely tune blood flow and pressure (8, 9). Their capacity to contract and relax facilitates the necessary adjustments in CBF to meet the heightened metabolic demands of the neuronal tissue (10). This intricate process, initiated early in development, is closely intertwined with the brain parenchyma itself (11, 12). In fact, neuronal activity and blood flow are coupled and meticulously regulated, supporting the concept that the health of the brain relies on the crosstalk and integration of a neurovascular unit, rather than the parenchyma alone (13, 14). Furthermore, regulation necessitates a high-level coordination with upper branches of the vascular tree, as, upon increased specific needs of flow in one region (hyperemia), upper branches must dilate to prevent reductions in downstream microvascular pressure (15). Consequently, within the brain, a well-coordinated flow response relies on vasodilation from distal to proximal arterial segments and myogenic mechanisms that enhance flow in response to decreased pressure.

As key regulators of CBF, we focused our investigation primarily on the alterations linked to aging in VSMCs and pericytes. The initial point was the identification of Notch signaling as the predominant molecular indicator of aging-related changes in blood vessels. In particular, our findings revealed a significant reduction in both Notch3 and Jagged1 during the aging process, accompanied by decreases in downstream targets and regulators of the signaling pathway. Building on these results, we embarked on an integrated analysis of patient samples to validate these findings and additional mechanistic experiments with animal models. Using a mouse with Notch3 inactivation, we comprehensively explored how absence of this signaling pathway affects the molecular physiology, cell biology, and vascular function of brain vessels during aging. In the process, we uncovered a critical connection between Notch signaling and vascular contractility with consequences beyond regulation of CBF.

## Results

### Single-cell transcriptomics reveals Notch3 decline as a molecular readout of vascular aging.

**Figure 1:**
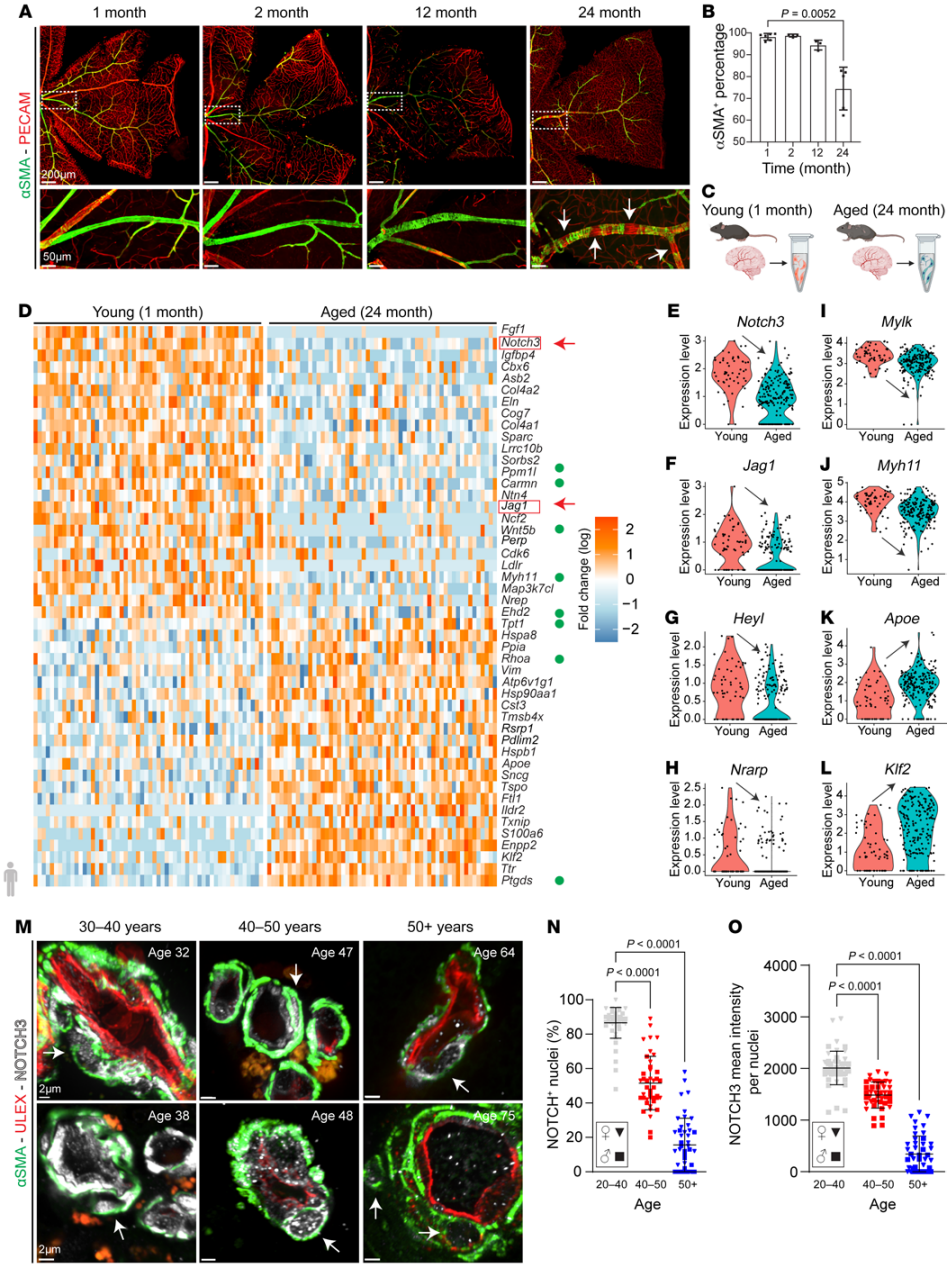
The aging vasculature experiences progressive loss of *Notch3* that leads to ongoing disorganization, dedifferentiation, and detachment of VSMCs. (**A**) Retinal vasculature from C57BL/6J mice at indicated ages. αSMA (green) identifies VSMCs, and PECAM (red) identifies endothelium. White arrows highlight VSMC loss. Scale bars: 200 μm (top row), 50 μm (bottom row). (**B**) Quantification of VSMC coverage at each time point from mixed-sex cohorts. Data are shown as the mean ± SD; *n* = 3–6. Welch’s *t* test. (**C**) Experimental design: Meningeal tissue and penetrating arteries were dissected from young (1 month) and aged (24 months) mice for scRNA-Seq. (**D**) Heatmap visualizing the top 50 differentially expressed genes (DEGs) in VSMCs. Green circles indicate genes that regulate muscle cell contraction. (**E**–**L**) Violin plots from selected transcripts. (**M**) Human brain vessel sections stained with αSMA (green) to visualize smooth muscle and NOTCH3 (white). White arrows indicate NOTCH3 in VSMC nuclei. Scale bars: 2 μm. (**N**) Quantification of NOTCH3^+^ nuclei in human brain VSMCs (20–50 μm vessel diameter) at indicated age ranges. (**O**) NOTCH3 mean intensity per nuclei in human brain VSMCs. For **N** and **O**, data are shown as the mean ± SD; *n* = 43–53 vessels, 6–7 patients per age group. Kruskal-Wallis with multiple testing correction.

To evaluate the cellular changes associated with vascular aging, we examined central retinal arteries of mice from 1 month to 24 months of age (Figure 1, A and B). The reason to use the retina relates to the stereotypical structure of its vasculature and the ease of identifying the same artery across multiple mice. Importantly, the retina also shares the embryological origin of vessels in the central nervous system. VSMC coverage of main retinal resistance arteries was complete and indistinguishable from 1 month up to nearly 12 months of age (Figure 1A). Thereafter and over time, we noted some minor disorganization in the arrangement of VSMCs followed by a precipitous loss of cells (Figure 1, A and B). Subsequently, we applied single-cell RNA sequencing (scRNA-Seq) on medium and small brain vessels to identify aging-associated transcriptional changes focusing specifically on VSMCs and pericytes (Figure 1C). After data quality control (Supplemental Figure 1, A–C; supplemental material available online with this article; https://doi.org/10.1172/JCI166134DS1), we clustered cells based on their expression profile using the Pagoda 2 pipeline (https://cran.r-project.org/web/packages/pagoda2/index.html) and identified 12 clusters that had approximately the same number of young and aged cells (Supplemental Figure 1, D and E). Clusters were further evaluated and annotated based on the expression of genes for cell type identity (Supplemental Figure 1, F and G). Vascular cells were identified by expression of *Pecam1* and *Cdh5* (endothelial cells); *Acta2* and *Myh11* (smooth muscle cells); and *Pdgfrb* and *Rgs5* (pericytes). In addition, we noted the presence of cortical neurons (*Pcp4* and *Enpp2*) and microglia (*Aif1* and *Trem2*) populations (Supplemental Figure 1, F and G). After strict quality controls, young and aged smooth muscle cells and pericytes were mined for transcriptional changes (Supplemental Tables 1 and 2).

A heatmap of the top 50 differentially expressed genes highlighted clear changes in genes associated with vascular smooth muscle contractile properties (Figure 1D, green dots). Importantly, of the 72 genes identified as significantly altered between young and aged VSMCs (adjusted *P* value < 0.05), 2 members of the Notch signaling pathway, *Notch3* and *Jagged1*, were within the top downregulated transcripts in aged VSMCs compared with controls (Figure 1, D–F). This in combination with downregulation of the downstream Notch targets *Heyl* and *Nrarp* (Figure 1, G and H) as well as decreased expression of contractile markers (*Mylk* and *Myh11*) (Figure 1, I and J) suggested that age-related loss of Notch signaling could be the driver of VSMC paucity. Upregulated genes included several heat shock and ER-stress transcripts (*Hspa8*, *Hsp90aa1*, *Hspb1*, *Txnip*) and prostaglandin H_2_ (*Ptgds*) (Figure 1D) as well as apolipoprotein E and Klf2 (*Apoe*, *Klf2*) (Figure 1, K and L).

Next, we examined expression of NOTCH3 in brain vessels from 27 human subjects ranging from 31 to 87 years of age who died from causes not associated with vascular dementia (Figure 1M). Evaluation of 115 small (1–2 VSMC layers) arteries from those patients revealed a consistent decline in nuclear and total NOTCH3 expression (Figure 1, N and O). These findings were consistent with the transcriptomics data from mice and pointed to Notch3 as an important molecular readout of aging in small brain vessels.

The Notch signaling pathway is essential to vascular morphogenesis, including VSMC recruitment, specification, and differentiation. These functions are mostly performed by *Notch1* during development (16–19). However, expression of *Notch3* emerges later in fetal life in arterial smooth muscle cells and remains as the predominant Notch receptor throughout adulthood (16). In fact, under physiological conditions, Notch3 is a marker for arterial mural cells (VSMCs and pericytes) (20). Surprisingly, inactivation of *Notch3* does not have deleterious effects on the viability of mice (21). Despite its high expression in VSMCs, large elastic arteries are relatively unaffected. In contrast, medium- and small-caliber arteries from Notch3-null mice experience loss of VSMCs in young adults (22, 23). Together, our data showing age-dependent reduction in Notch3 and loss of VSMCs in wild-type (WT) mice, and the published findings linking this gene with VSMC coverage, suggested that Notch3 might be a key regulator of VSMC aging.

### Inactivation of Notch3 results in accelerated VSMC aging with progressive dedifferentiation and detachment of VSMCs that leads to vascular abnormalities first manifested in the brain.

To characterize the time-dependent dynamics of VSMC loss in *Notch3^–/–^* animals, retinal VSMC coverage in *Notch3^–/–^* mice was quantified at multiple time points from 2 weeks to more than 104 weeks of age (Supplemental Figure 2A). At 2 weeks, arteries from *Notch3*-null mice were indistinguishable from control (Supplemental Figure 2B). Nonetheless, by 4 weeks and subsequent ages until 2 years old, we found progressive disorganization and detachment of VSMCs from main retinal arteries. Essentially, a 4-week-old *Notch3^–/–^* artery was equivalent to a 2-year-old artery from WT mice in terms of VSMC loss. Interestingly, VSMC detachment was not as pronounced in precapillary branches (Supplemental Figure 2D), indicating that it was the resistance arteries where the phenotype was most severely manifested. Brain resistance arteries were equally impacted by loss of *Notch3*. Specifically, we found that while large arteries like the carotid showed no apparent abnormalities, the middle cerebral artery and pial arteries showed loss of VSMCs (Supplemental Figure 3, A and B) followed by significant (2- to 3-fold) vessel enlargement in older mice (Supplemental Figure 3, C and D). We also found that reduction in smooth muscle cell coverage was preceded by decline in VSMC differentiation markers; in particular, calponin (*Cnn1*) was exquisitely sensitive to inactivation of *Notch3* (Supplemental Figure 3E).

**Figure 2:**
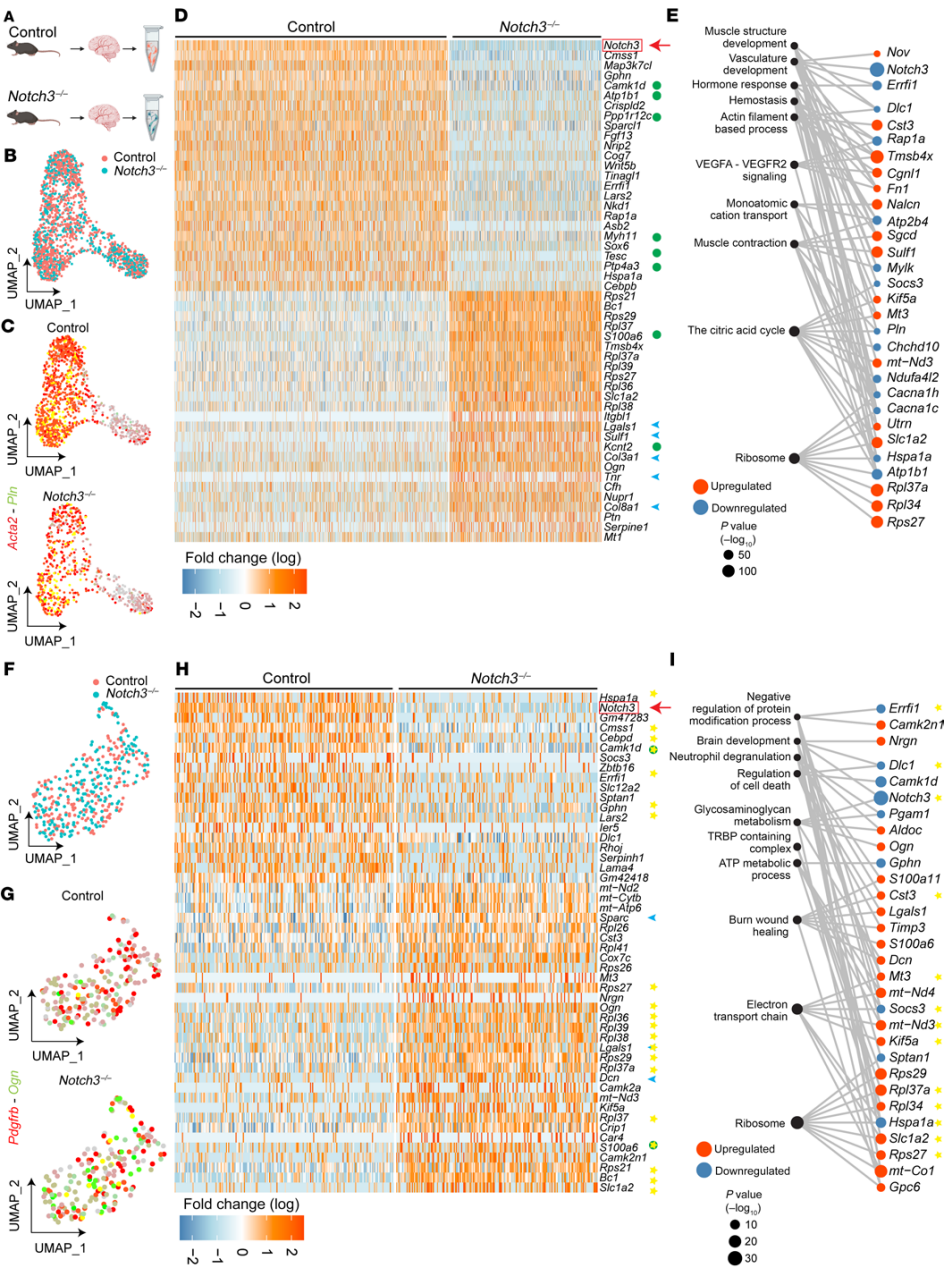
Loss of *Notch3* in VSMCs and pericytes results in a decrease in transcripts that regulate contractility and an increase in transcripts associated with extracellular matrix. (**A**) Schema of experimental design. Superficial brain vessels and penetrating brain arteries were dissected from mature (12-month-old) female and male *Notch3^–/–^* and littermate control mice and enzymatically dissociated to obtain single-cell suspensions for scRNA-Seq. *n* = 8 total mice, 2 female and 2 male pooled per genotype. (**B**) Uniform manifold approximation and projection (UMAP) plot of scRNA-Seq visualizing spread of data from VSMCs of the 2 genotypes. (**C**) Feature plot identifies *Acta2^+^Pln^+^* VSMCs in control and *Notch3^–/–^*. (**D**) Heatmap visualizing the top 50 DEGs identified in VSMCs. (**E**) Gene Ontology enrichment of *Notch3^–/–^* VSMCs from the top 10 unique ontology categories and selected member genes within each category. Dot color indicates direction of expression change upon loss of *Notch3^–/–^*, while size indicates significance of enrichment. (**F**) UMAP plot of scRNA-Seq visualizing spread of data from pericytes of the 2 genotypes at 12 months of age. (**G**) Feature plot identifies *Pdgfrb^+^Ogn^+^* pericytes in control and *Notch3^–/–^*. (**H**) Heatmap visualizing the top 50 DEGs identified in pericytes. (**I**) Gene Ontology enrichment of *Notch3^–/–^* pericytes from the top 10 unique ontology categories and selected member genes within each category. Dot color indicates direction of expression change upon loss of *Notch3^–/–^*, while size indicates significance of enrichment. Yellow stars indicate genes identified as DEGs in the same direction in both *Notch3^–/–^* VSMCs and *Notch3^–/–^* pericytes. For **D** and **H**, green circles indicate genes that regulate muscle cell contraction; blue arrowheads indicate ECM transcripts. Yellow stars indicate genes identified as DEGs in the same direction in both *Notch3^–/–^* VSMCs and *Notch3^–/–^* pericytes.

To understand the molecular mechanisms associated with the observed vascular abnormalities in *Notch3*-null mice, we performed single-cell transcriptomics on a cohort of *n* = 8 twelve-month-old (mature) *Notch3^–/–^* and WT littermates (Figure 2A). After quality control of the data (Supplemental Figure 4, A–H), we focused our evaluation on VSMCs and pericytes (Figure 2 and Supplemental Tables 1 and 2). As anticipated, we were able to collect more WT cells than Notch3-null cells, in keeping with their progressive loss with age; nonetheless, the recovery of over 1,000 VSMCs in the control and over 700 VSMCs in the null mouse enabled a robust analysis (Figure 2B). While VSMCs from both genotypes expressed α-smooth muscle actin (αSMA), levels of phospholamban (*Pln*), a gene product that regulates sarcoplasmic reticulum Ca^2+^-ATPase, were lower in *Notch3*-null than in WT cells (Figure 2C), suggesting differences in contractile function. In fact, a heatmap of the top 50 up- and downregulated genes revealed a signature highlighting loss of contractile properties (Figure 2D, green dots) and gain of extracellular matrix (Figure 2D, blue arrowheads). For example, a marked reduction in transcripts for proteins associated with calcium regulation (*Pln*, *Mylk*), sodium/potassium transport (*Atp1b1*), and muscle structure (*Myh11*, *Sgcd*, *Utrn*) underscored disrupted contractility and altered VSMC identity. Similarly, upregulation of many matrix transcripts (*Col3a1*, *Col8a1*, *Lgals1*, *Ogn*, and *Sulf1*) indicated higher synthesis of matrix proteins with resulting stiffness/fibrosis of the vasculature. Analysis of Gene Ontology categories is consistent with a phenotype that is deficient in supramolecular muscle organization and committed to increase deposition of matrix proteins (Figure 2E).

It is well established that biological sex contributes to vascular differences in the context of aging, including modulation of smooth muscle cell contractility and vascular stiffness (24). The initial 12-month scRNA-Seq data set was generated with pooled samples of equivalent female and male mice to avoid sex bias in the identification of Notch3 regulated genes. Using classical X and Y chromosome transcripts, we reidentified sex from the pooled libraries to assess whether sex was a modulator of the Notch3 effect in VSMCs (Supplemental Figure 5A). We identified 486 female and 407 male cells in control and 226 female and 179 male cells in *Notch3^–/–^* VSMCs. In addition, we identified a third set of cells, labeled N/A due to lack of clear expression of *Xist* or any of the Y chromosome markers. N/A cells made up 278 control and 247 Notch3-null cells (Supplemental Figure 5B) and were not used in the analysis. Differential expression of the sex-segregated data sets identified 327 (male to male) to 356 (female to female) transcripts between Notch3-null and control VSMCs. Overlay of these 2 data sets showed a strong overlap between sexes with 225 genes as shared between comparisons (Supplemental Figure 5, C and D). When these sex-stratified gene signatures were then compared with the top 50 VSMC differentially expressed genes in the original data set, we found up to 99% concurrence between the sex-specific comparisons and joint data, supporting the conclusion that biological sex does not act as a modifier of Notch3 in cerebral vascular VSMCs.

Pericytes were also affected by the loss of *Notch3* (Figure 2, F–I), and their differentially expressed gene signature overlapped (by 69%–75%) with the differentially expressed gene signature from VSMCs (Figure 2H, yellow stars). This concurrence in transcriptional profiles from 2 different cell types strongly suggested a core gene program that was most likely regulated by *Notch3* (Figure 2H). Gene Ontology enrichment analysis of *Notch3^–/–^* pericytes revealed alterations in proteins related to glycosaminoglycan metabolism (*Dcn*, *Ogn*, *Gpc6*, *Aldoc*, and *Pgam1*) and wound healing (Figure 2I). Given the critical decrease in transcripts associated with contractility and the increase in matrix proteins, we hypothesized that over time *Notch3^–/–^* mice would progressively show structural abnormalities and deficiencies in contractility.

**Figure 3:**
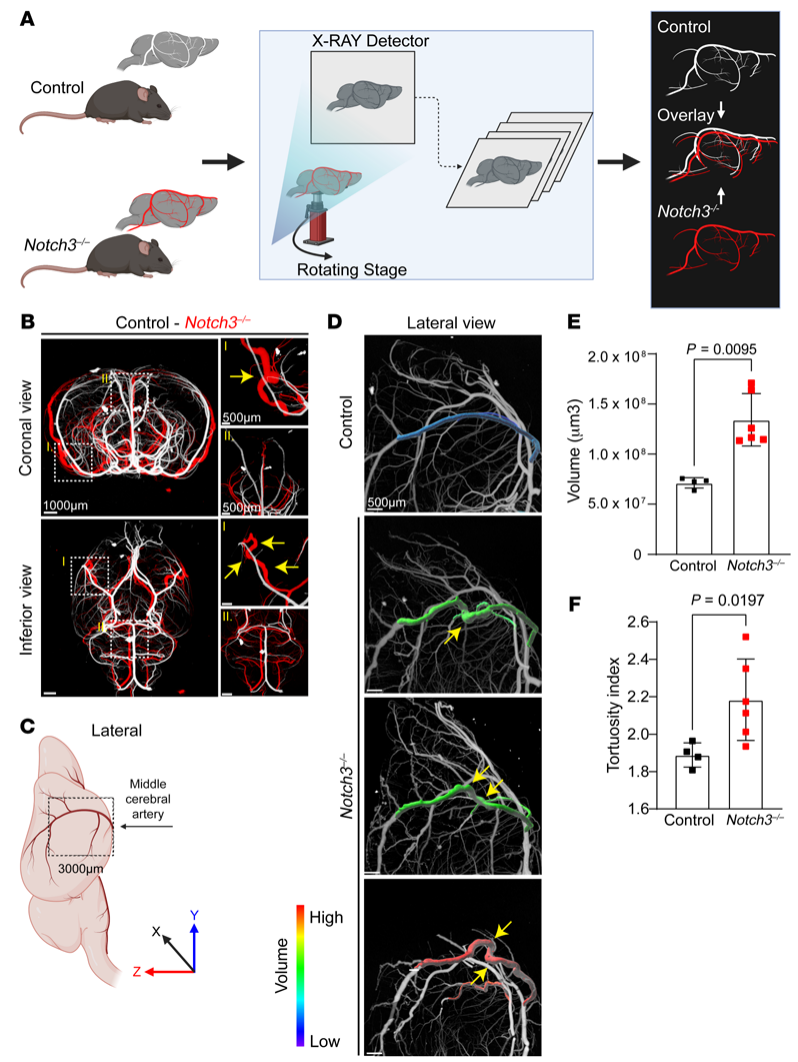
Notch3 deficiency leads to vascular dilation and tortuosity in the middle cerebral artery. (**A**) Schema of experimental analysis whereby micro-CT overlay of the brain vasculature of *Notch3^–/–^* (red) and control (white) mice identifies abnormalities. (**B**) Coronal and inferior micro-CT overlay of *Notch3^–/–^* and control animals at 18 months. Yellow arrows indicate tortuosity and aneurysms in *Notch3^–/–^* compared with control. Scale bars: 1000 μm (left), 500 μm (right). (**C**) Schema of the regions of middle cerebral artery (MCA) measured in **D**. (**D**) Volumetric analysis of the MCA from the circle of Willis to 3,000 μm in *Z* axis (from the MCA branch point upward) in control and *Notch3^–/–^*. Maximum-intensity projections of MCA were color-coded by volume and overlaid on the base micro-CT visualization (white) in the lateral view. Yellow arrows indicate tortuosity. Scale bars: 500 μm. (**E**) Quantification of MCA volumes from *Notch3^–/–^* and control littermates at 18 months. *n* = 4–6; Mann-Whitney test. (**F**) Quantification of tortuosity index for the MCA in a cohort of control and *Notch3^–/–^* animals. *n* = 4–6; Welch’s *t* test.

**Figure 4:**
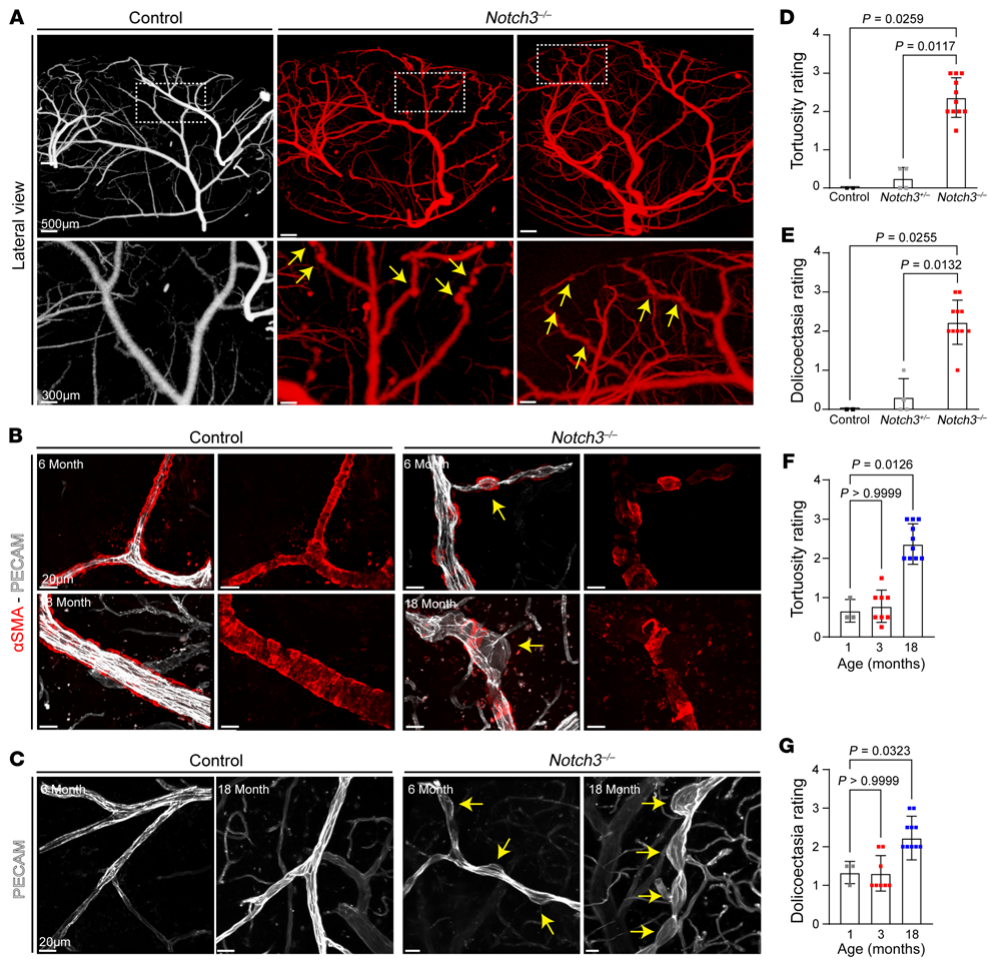
Microaneurysms and tortuosity in *Notch3^–/–^* mice are associated with progressive loss and disorganization of VSMCs. (**A**) Representative micro-CT images of microaneurysms in higher-order branches of the MCA. Yellow arrows indicate points of dilation. (**B**) Images illustrating microaneurysms and tortuosity in aging *Notch3^–/–^* brain penetrating arteries in the context of VSMC loss at 6 and 18 months as indicated. Yellow arrows indicate aneurysms in *Notch3^–/–^* animals. (**C**) Images of microaneurysms in aging *Notch3^–/–^* brain vessels. Yellow arrows point to dilations (PECAM, white). (**D**) Tortuosity rating in WT, heterozygous, and null mice. *n* = 3–11; Kruskal-Wallis test. (**E**) Dolichoectasia rating across three *Notch3* genotypes. *n* = 3–11; Kruskal-Wallis test. (**F**) Tortuosity rating at 3 progressive time points in *Notch3^–/–^* animals. *n* = 4–10; Kruskal-Wallis test. (**G**) Dolichoectasia at 3 progressive time points in *Notch3^–/–^* animals. *n* = 4–10; Kruskal-Wallis test. For **D** and **F**, numbers indicate severity (1, zero to minimal vasculature tortuosity; 3, severe tortuosity across 7–15 vessels per animal). For **E** and **G**, numbers indicate severity (1, zero to minimal dolichoectasia; 3, severe dolichoectasia across 10–15 vessels per animal).

To examine structural changes in the brain vasculature, we performed micro-CT on aged littermate WT and *Notch3^–/–^* mice (Figure 3A). From these evaluations, a consistent micro-CT signature emerged: Notch3-null aged mice exhibited tortuosity, vascular enlargement, and microaneurysms. Remarkably, enlargement of medium-sized vessels like the middle cerebral artery was only present as the artery ascended on the lateral aspects of the brain, suggesting that it was topologically associated with areas that required increased contractile strength (Figure 3B, overlapping vasculatures of null mice [red] and WT littermate [white]). In those areas, the vessel showed a 3- to 4-fold increase in volume and increased tortuosity (Figure 3, C–F, and Supplemental Figure 6, A and B). Secondary branches of the middle cerebral artery were characterized by beading (dilations and constrictions) along the course of the vessels in aged Notch3-null, but not in WT or heterozygous littermates (Figure 4, A–G). The microaneurysms were associated with disorganization or absence of smooth muscle cell coverage (Figure 4B). Beading of the vessel and development of microaneurysms were age dependent and were only observed after 6 months of age with progression over time (Figure 4, C, F, and G). Importantly, while reductions in VSMC coverage were also noted in other resistance arteries systemically in aged mice (Supplemental Figure 6C), the presence of microaneurysms and beaded vessels was confined to the brain.

### Notch3 is essential to maintain the contractile phenotype of VSMCs.

**Figure 5:**
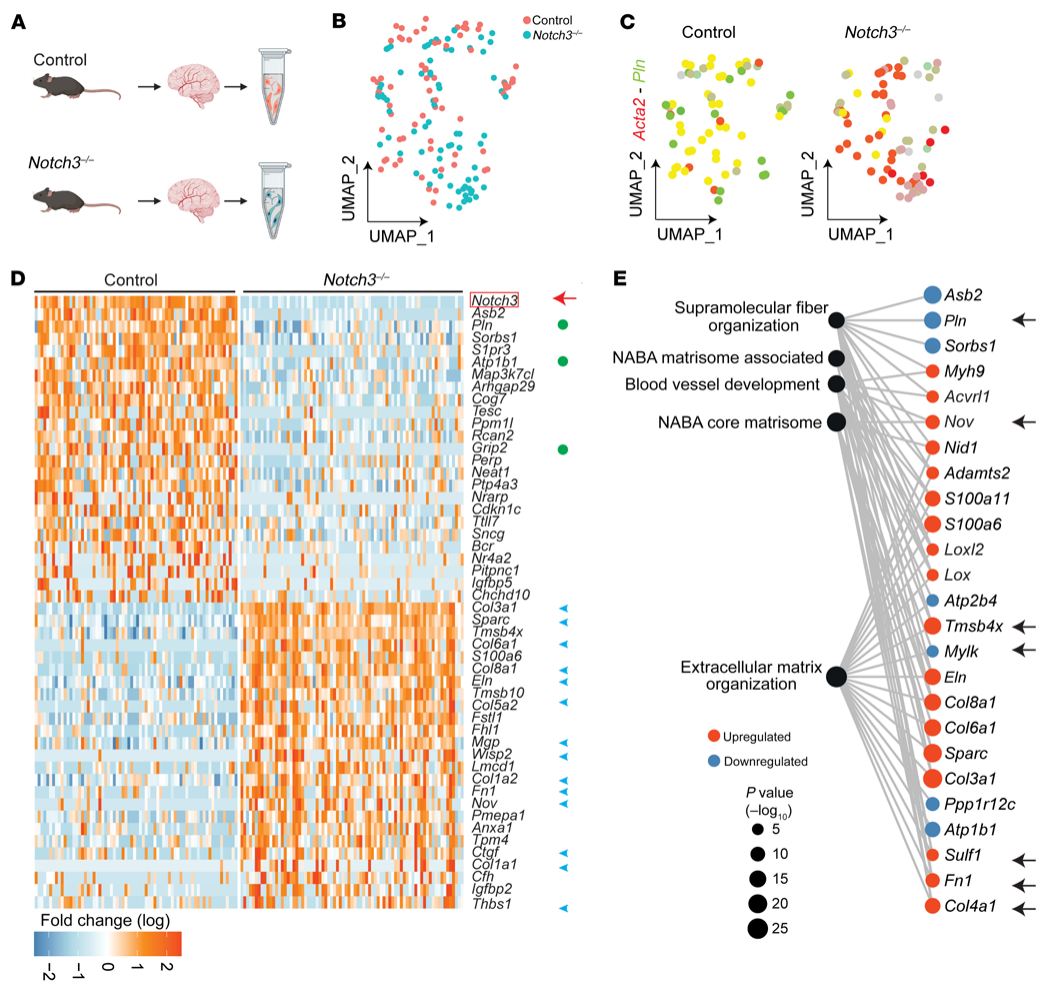
Transcriptional signature associated with *Notch3* loss at 1 month in VSMCs. (**A**) Graphical illustration of experimental design. Superficial brain vessels and penetrating brain arteries were dissected from 1-month *Notch3^–/–^* and littermate control mice and enzymatically dissociated to obtain single-cell suspensions for scRNA-Seq. (**B**) UMAP plot of scRNA-Seq visualizing spread of data from VSMCs of the 2 genotypes. (**C**) Feature plot identifies *Acta2^+^Pln^+^* VSMCs in control and *Notch3^–/–^*. (**D**) Heatmap visualizing the top 50 DEGs identified in VSMCs. Green circles indicate genes that regulate muscle cell contraction; blue arrowheads indicate ECM transcripts. (**E**) Gene Ontology enrichment of *Notch3^–/–^* VSMCs for the top 5 unique ontology categories and selected member genes within each category. Dot color indicates the direction of expression change upon loss of *Notch3* expression, while size indicates significance of enrichment. Arrows highlight gene products that were selected for validation using immunofluorescence (see Supplemental Figures).

**Figure 6:**
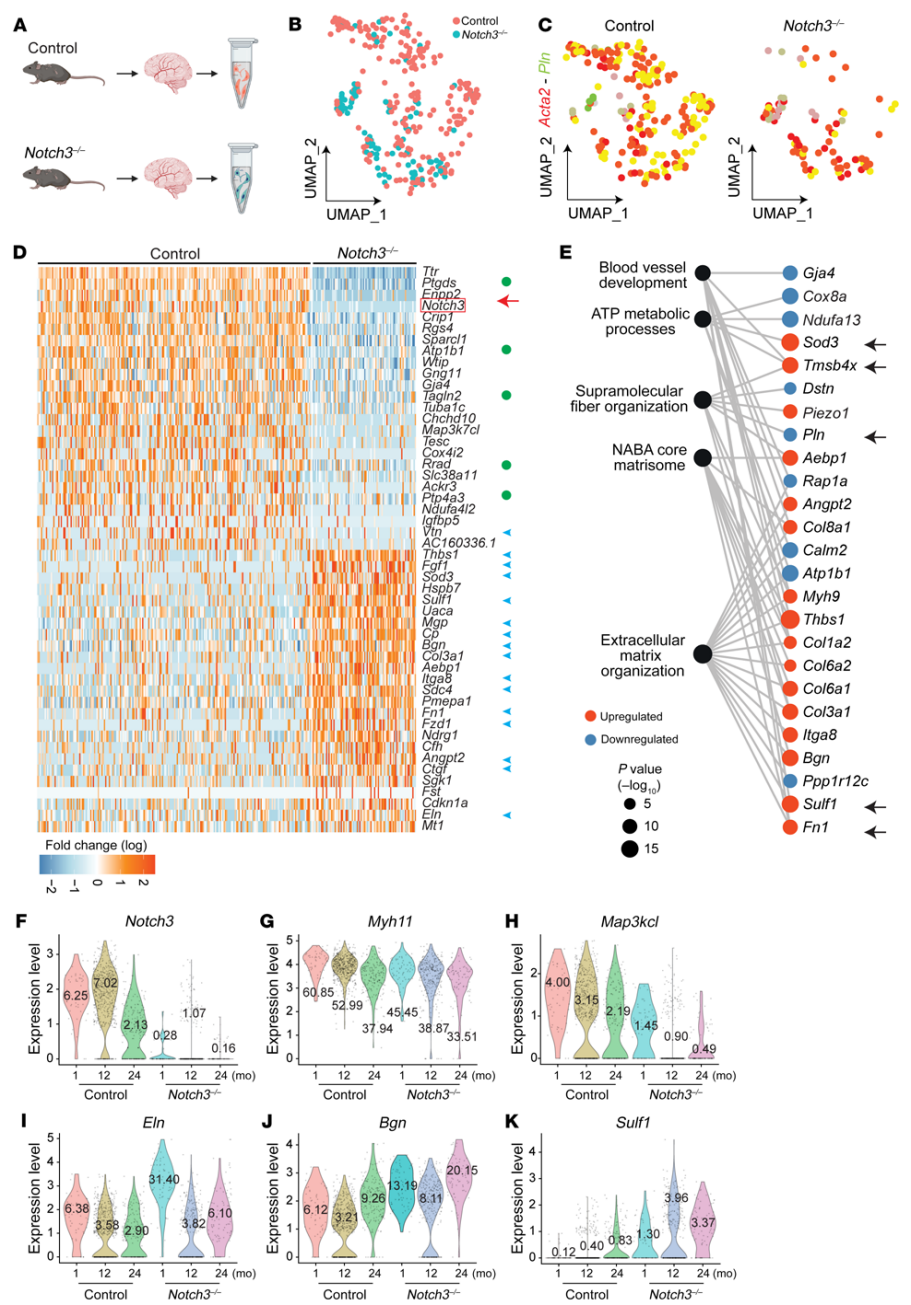
Transcriptional signature associated with Notch3 deficiency at 24 months in VSMCs. (**A**) Graphical illustration of experimental design. Superficial brain vessels and penetrating brain arteries were dissected from 24-month *Notch3^–/–^* and littermate control mice and enzymatically dissociated to obtain single-cell suspensions for scRNA-Seq. (**B**) UMAP plot of scRNA-Seq visualizing spread of data from VSMCs of the 2 genotypes. (**C**) Feature plot identifies *Acta2^+^Pln^+^* VSMCs in control and *Notch3^–/–^*. (**D**) Heatmap visualizing the top 50 DEGs identified in VSMCs. Green circles indicate genes that regulate muscle cell contraction; blue arrowheads indicate ECM transcripts. (**E**) Gene Ontology enrichment of *Notch3^–/–^* VSMCs from the top 5 unique ontology categories and selected member genes within each category. Dot color indicates the direction of expression change upon loss of *Notch3* expression, while size indicates significance of enrichment. Arrows highlight the gene products that were selected for validation by immunofluorescence (see Supplemental Figures). (**F**–**K**) Violin plots from selected transcripts across ages and genotypes.

Considering the progressive age-dependent effects in the Notch3-deficient vasculature, we performed 2 additional scRNA-Seq experiments at 1 month and 24 months of age. The objective was to potentially identify direct targets (1 month; Figure 5), but also to highlight the downstream compounded effect of aging in the context of Notch3 inactivation (24 months; Figure 6). Differential gene expression analysis at 1 month revealed that VSMCs from WT and null mice were already distinguishable (Figure 5, A–C, Supplemental Tables 1 and 2, and Supplemental Figure 7, A–H). A heatmap of the top 25 upregulated and downregulated genes at 1 month uncovered a signature with loss of contractile properties (green dots) and gain of matrix synthesis (blue arrowheads; Figure 5D). For example, a marked reduction in transcripts for proteins associated with calcium regulation (*Pln*, *Rcan2*), sodium/potassium transport (*Atp1b1*, *Tesc*), and Rho regulation (*Arhgap29*) underscored disrupted contractility. Similarly, upregulation of many matrix transcripts (*Col3a1*, *Sparc*, *Col6a1*, *Col8a1*, *Eln*, *Col5a2*, *Mgp*, *Col1a1*, *Thbs1*) implied deposition of matrix proteins with resulting stiffness/fibrosis of the vasculature. Transcriptional increase in matrix proteins was further supported by upregulation of matrix metalloproteinase inhibitors that block matrix remodeling (*Timp3*) and of connective tissue growth factor (*Ctgf*) and *Pmepa1*, which also regulate TGF-β signaling (also identified previously; refs. 18, 19). At 1 month, we also noticed the upregulation of 2 thymosins (*Tmsb4x* and *Tmsb10*) that bind and sequester actin monomers, as well as an increase in tropomyosin alpha 4 (*Tpm4*), possibly a compensatory response to rectify deficiencies in contraction. Analysis of Gene Ontology categories is consistent with a phenotype that is deficient in supramolecular fiber organization and committed to increase deposition of matrix proteins (Figure 5E).

A similar differential gene expression analysis was performed at 24 months to compare WT and *Notch3-*null littermates (Figure 6, A–C; Supplemental Tables 1 and 2; and Supplemental Figure 8, A–H) and elucidate the compounded effect of age and absence of *Notch3*. The total cell recovery during isolation of VSMCs in the 2-year-old cohort of null mice was lower than that from control, an expected outcome associated with the progressive loss of VSMCs already discussed. Nonetheless, the findings from this third scRNA-Seq provided further support and rigor for the findings with a sizable number of differentially expressed transcripts overlapping between all three data sets.

These findings clarified changes that were associated with aging and those that were more inherent to direct regulation by *Notch3* (Supplemental Figure 9). Consistent with the 1-month and 12-month data, at 24 months of age we also found a decrease in transcripts for genes that regulated calcium levels, with some of these overlapping (*Pln*, *Calm2*) and others new (*Rrad*). The data also showed consistency in the reduction of sodium/potassium transport transcripts (*Atp1b1*, *Tesc*), as well as changes in prostaglandin D_2_ production (*Ptgds*, *Enpp2*). Much like at 1 month and 12 months, transcripts for some extracellular matrix proteins were also increased in 24-month null animals, including *Thbs1*, *Mgp*, *Col3a1*, and *Fn1* among the top 25 upregulated genes. In addition, we noted upregulation in proteoglycans, particularly biglycan (*Bgn*) and syndecan 4 (*Sdc4*); a significant increase in extracellular sulfatase 1 (*Sulf1*), which regulates sulfatioæ’n of proteoglycans extracellularly; and upregulation of transcripts associated with stress-induced apoptosis (*Sod3*, *Uaca*, *Ndrg1*) (Figure 6D). Gene Ontology enrichment analysis, similar to observations from the 1-month mice, identified deficiencies in supramolecular fiber organization and increases in extracellular matrix, but it also found deficiencies in ATP metabolism that highlight evidence for biological stress in smooth muscle cells (Figure 6E). To place the findings in perspective, we combined all 3 data sets and compared WT with *Notch3*-null smooth muscle cells over time (Figure 6, F–K). In WT cells, Notch3 transcripts decreased with aging, as did the contractility transcripts *Myh11* and *Map3k7cl*; this decrease was more pronounced in the absence of *Notch3*. In contrast, matrix proteins such as elastin, biglycan, and *Sulf1* significantly increased upon loss of *Notch3*.

Findings were also validated at the protein level by immunocytochemistry of a cohort of up- and downregulated genes. We found consistent changes for all 8 of the targets tested in the middle cerebral artery (Supplemental Figure 10, A–H). Inactivation of *Notch3* also affected pericytes with similar outcomes to smooth muscle cells (Supplemental Figure 11, A–C, and Supplemental Table 2). Finally, we also found alterations in brain endothelial cells, which likely relate to dysfunctional endothelial cell–mural cell interactions, as endothelial cells do not express Notch3 (Supplemental Figure 11, D–F, and Supplemental Table 2). Importantly, previous publications have documented alterations in the blood-brain barrier of *Notch3*-null young adults (23).

**Figure 7:**
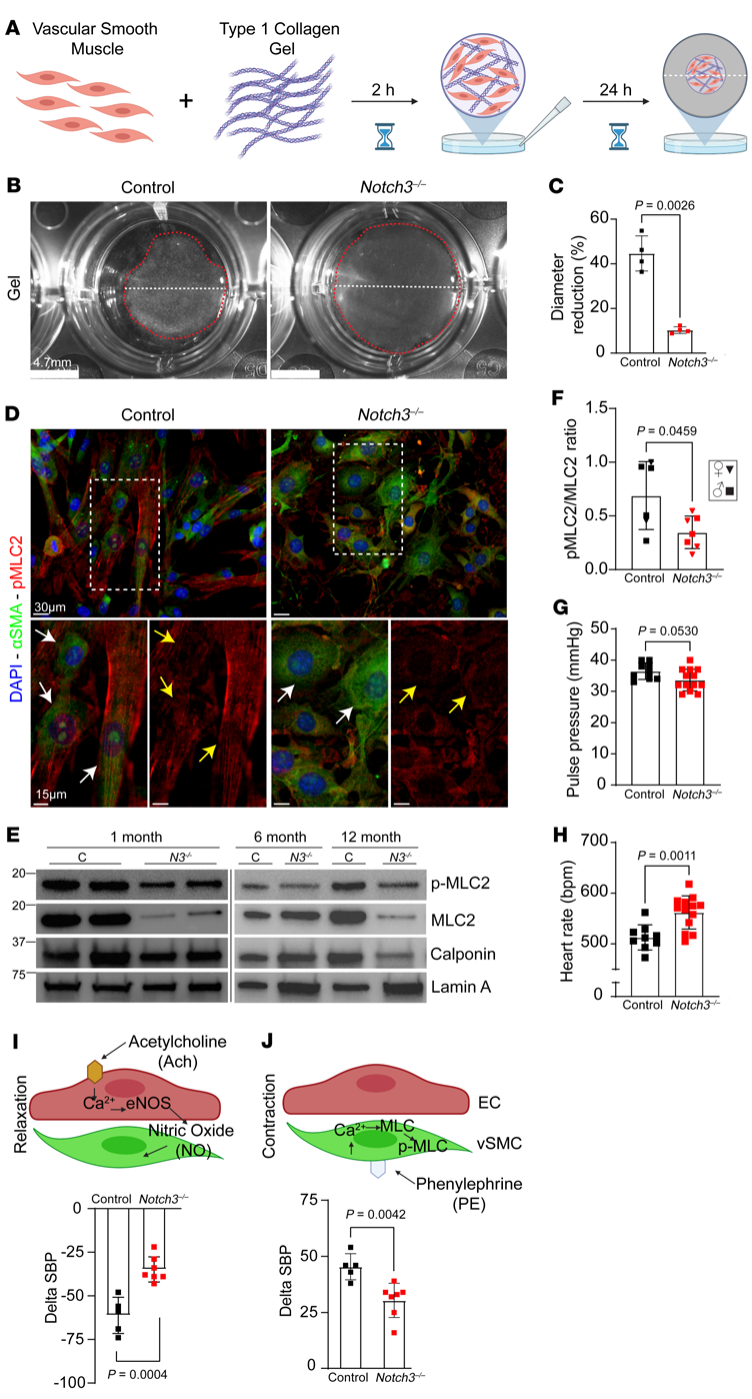
*Notch3* deficiency results in vascular dysfunction due to impaired contractility. (**A**) Diagram of experimental design. VSMCs from 1-month control and *Notch3^–/–^* mice were isolated and mixed with type I collagen to form a cellular hydrogel, detached, and observed 24 hours later. (**B**) Images of polymer gels from control and *Notch3^–/–^* cells after 24 hours. Note the differences in the contraction of the gel with regard to diameter (white dashed line) and total size of gel (red dashed line). (**C**) Quantification of percent gel diameter reduction 24 hours after plating. Bars indicate mean ± SD; *n* = 4 biological replicates; Welch’s *t* test. (**D**) Immunofluorescence of phosphorylated myosin light chain 2 (p-MLC2; red). White arrows highlight αSMA (green); yellow arrows highlight p-MLC2 (red). (**E**) VSMC-enriched lysates from mixed-sex cohorts of *Notch3^–/–^* and control aortae at the indicated ages were evaluated for expression of p-MLC2, MLC2, calponin, and lamin A (loading control). (**F**) Quantification of p-MLC2/MLC2 from a 1-month mixed-sex cohort. Data are shown as the mean ± SD; *n* = 6–7; Welch’s *t* test. Female data are indicated by inverted triangles, male data by squares. (**G**) Pulse pressure in 18-month-old male control and *Notch3^–/–^* mice. (**H**) Heart rate measured in 18-month male control and *Notch3^–/–^* mice. (**I**) Simplified diagram of the molecular pathway of acetylcholine-driven relaxation in VSMCs and quantification of systolic blood pressure response to acetylcholine in 18-month male control and *Notch3^–/–^* animals. (**J**) Simplified diagram of the molecular pathway of phenylephrine-driven contraction in VSMCs and quantification of systolic blood pressure response to phenylephrine in 18-month male control and *Notch3^–/–^* animals. For **G** and **H**, data are shown as the mean ± SD; *n* = 9–13, unpaired Student’s *t* test. For **I** and **J**, data are shown as the mean ± SD; *n* = 5–7, unpaired Student’s *t* test.

Next, given the apparent direct effect of *Notch3* on the expression of key contractile genes such as *Mylk*, we examined the functional effects of Notch3 loss on VSMC contractility. The molecular changes identified by scRNA-Seq were physiologically tested in collagen contraction assays using smooth muscle cells isolated from 1-month WT and *Notch3^–/–^* littermates (Figure 7, A and B). In fact, absence of *Notch3* impaired contractility. WT cells were able to contract collagen gels by 43% in 24 hours, in contrast to only 8% contraction by *Notch3-*null cells for the same time period, representing a 5-fold difference in contractile function. This effect was consistently reproduced with VSMCs isolated from 4 independent mouse cohorts of both sexes (Figure 7C). Evaluation of VSMCs in vitro confirmed loss of the contractile phenotype and acquisition of a synthetic phenotype by cells that lacked *Notch3*. In fact, while αSMA was present in both WT and null cells, the levels of phospho–myosin light chain 2 (p-MLC2) were significantly reduced in *Notch3^–/–^* cells. Furthermore, the elongated morphology of WT cells contrasted with the polygonal aspect of null cells (Figure 7D). Both attributes were consistent with the synthetic phenotype that has been previously ascribed when smooth muscle cells lose contractile properties (14). Reduction of total and phosphorylated MLC2 was also confirmed on protein lysates from aortae of WT and null mice at 1, 6, and 12 months (Figure 7E, see supplemental material for full, uncut gels) and further quantified using multiple independent lysates from control and null mice at 1 month of age (Figure 7F and Supplemental Figure 12, see supplemental material for full, uncut gels).

Physiological assessment of the aged *Notch3^–/–^* animals showed altered cardiovascular parameters including pulse pressure and heart rate, suggestive of attempted chronic compensation for poor vascular reactivity (Figure 7, G and H). In vivo hemodynamic responses to cholinergic and adrenergic stimuli were significantly affected in mutant mice (Figure 7, I and J). The findings were consistent with an inability of smooth muscle cells to contract and revealed inadequate vasoreactivity.

### Absence of Notch3 leads to chronic cerebral hypoperfusion, glymphatic dysfunction, and neurodegeneration.

**Figure 8:**
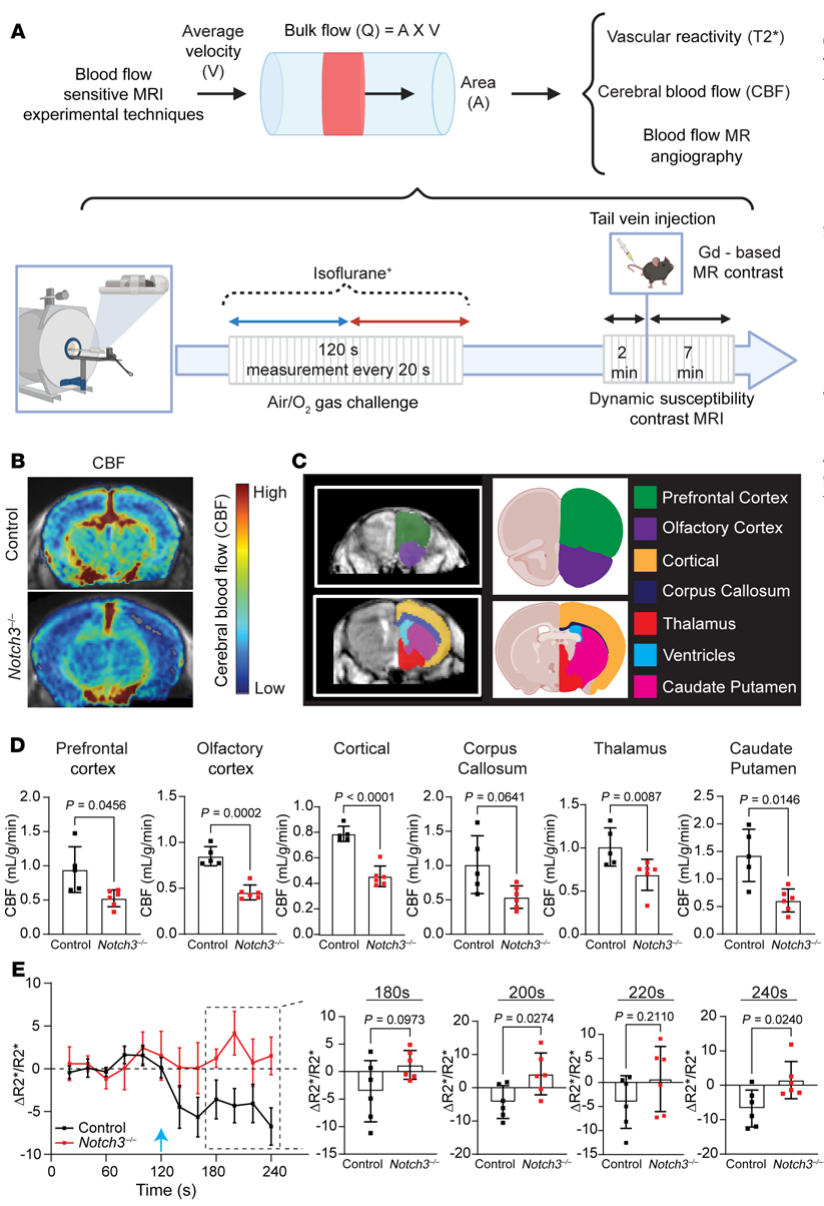
*Notch3* deficiency delays cerebral vascular blood flow. (**A**) Schematic depiction of the MRI techniques used to obtain blood flow parameters and timing of acquisition. (**B**) Representative MRI image with superimposed cerebral blood flow (CBF) parameters obtained from dynamic susceptibility contrast MRI for each group. (**C**) Regional map of the brain regions used for CBF measurements. (**D**) Quantification of CBF measurements at the indicated regions. (**E**) Average R2* MRI during oxygen challenge across control and *Notch3^–/–^* animals. The blue arrow indicates the time at which the switch between room air and oxygen occurred during the experimental design indicated in **A**. For **D** and **E**, *n* = 6 animals per group in each genotype; Welch’s *t* test. Data are shown as the mean ± SD (**D**) and ± SEM (**E**).

The limitations in VSMC contractility were further supported by MRI studies (Figure 8A). MRI evaluation showed a reduction in cerebral blood flow across multiple regions in *Notch3^–/–^* mice when compared with controls (Figure 8, B–D). Dynamic multi-gradient echo sequence was used to acquire sequential R2* maps between air-gas exchange to assess cerebral oxygenation (Figure 8E). These studies confirmed impaired oxygenation of the prefrontal cortex in *Notch3^–/–^* mice, supporting the conclusion that deficient vasoreactivity in *Notch3*-null mice contributes to poor oxygenation in comparison with control littermates, leading to chronic hypoperfusion.

**Figure 9:**
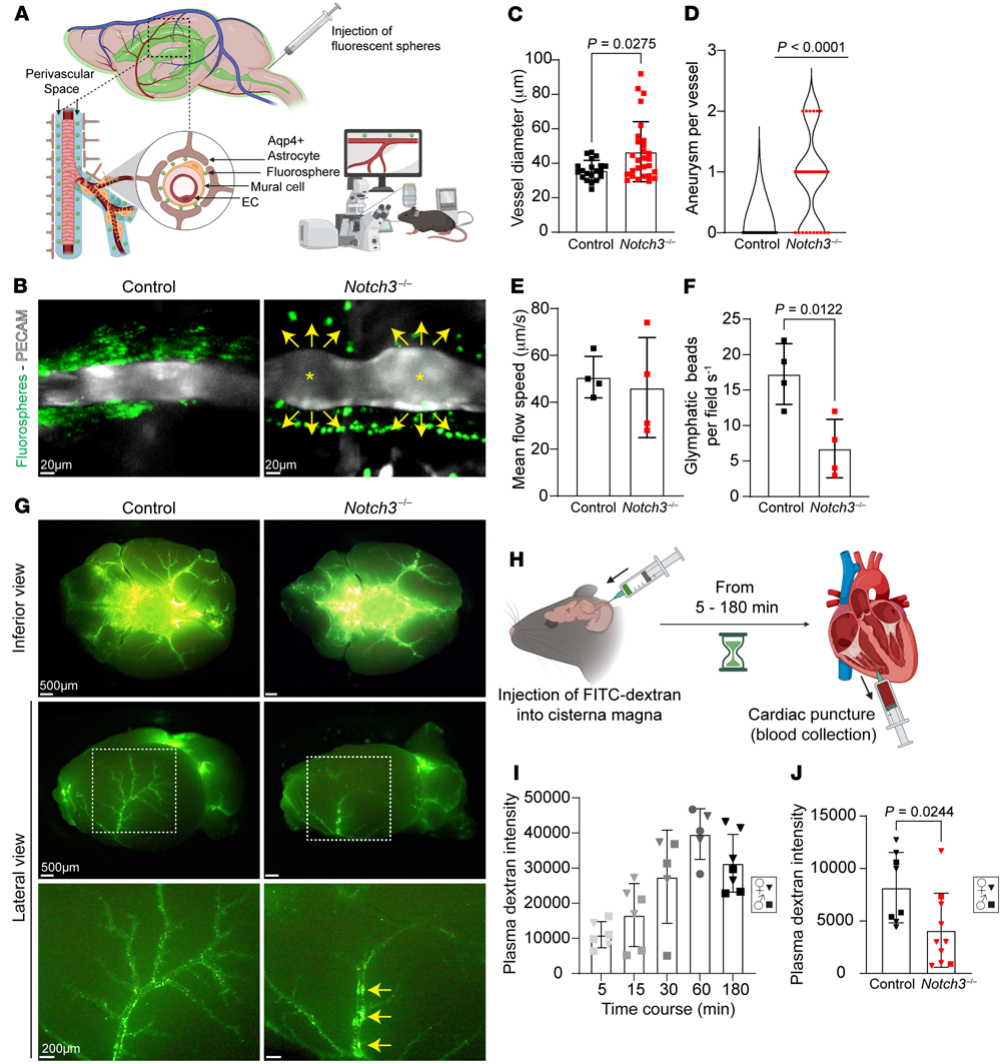
*Notch3* deficiency delays glymphatic flow. (**A**) Experimental design. Mice were anesthetized and injected with PECAM antibodies. A cannula inserted in the cisterna magna delivered fluorescent beads, which reached the perivascular space and were visualized by live imaging from the intact skull. (**B**) In vivo images of PECAM-labeled vessels in *Notch3^–/–^* and control. Arrows indicate flow of FITC-beads; asterisks indicate aneurysms. (**C**) Quantification of vessel diameter (from live images). Data are shown as the mean ± SD; *n* = 20 vessels, 5 animals (control), and 28 vessels, 8 animals (*Notch3^–/–^*), from mixed sexes at 6 months. Mann-Whitney test. (**D**) Quantification of aneurysms per vessel. *n* = 20 vessels, 5 animals (control), and 28 vessels, 8 animals (*Notch3^–/–^*), from mixed sexes at 6 months. One-sample Wilcoxon’s test. (**E**) Quantification of mean bead velocity. (**F**) Quantification of beads per visual field in mixed sexes at 6 months. Data are shown as the mean ± SD; *n* = 4; Welch’s *t* test for **E** and **F**. (**G**) Representative inferior and lateral images of *Notch3^–/–^* and control brains harvested 3 hours after cisternal injection. Yellow arrows indicate bead stagnation. (**H**) Experimental design. Mice were injected with FITC-dextran into the cisterna magna; blood was collected by cardiac puncture to assess fluorescence as a measurement of glymphatic clearance. (**I**) Quantification of fluorescence intensity after cisternal FITC-dextran injection across multiple time points in a mixed-sex cohort of 3-month C57BL/6J mice. *n* = 5–7 animals per time point and/or condition, as indicated in graph; unpaired Student’s *t* test. (**J**) Quantification of plasma fluorescence intensity 5 minutes after cisternal FITC-dextran injection in a mixed-sex cohort of 6-month *Notch3^–/–^* and control animals. Data are shown as the mean ± SD; *n* = 8–10 animals per time point and/or condition, as indicated; Welch’s *t* test. For **I** and **J**: squares, male; inverted triangles, female.

It has been demonstrated that poor vascular contractility affects the glymphatic system, a perivascular network that subserves a pseudo-lymphatic function and promotes fluid balance and interstitial waste removal (25). To ascertain the effect of compromised vascular reactivity on the glymphatic system, we first injected fluorescent beads into the cisterna magna and observed their flow in anesthetized mice whose blood vessels had been labeled by a non-blocking anti-PECAM monoclonal antibody (Figure 9A). Supporting our previous findings, evaluation of PECAM-labeled arteries of live mice consistently showed dilations in the null mouse and narrow straight arteries in WT littermates (Figure 9, B–D, and Supplemental Videos 1 and 2). Fluorescent beads were found to travel in the perivascular space in both groups; however, the number of beads was notably reduced in null mice (Figure 9, B, E, and F, and Supplemental Videos 1 and 2). The distribution of beads in the perivascular space was also different, showing flow close to the vessel in control and farther from the vessel in the Notch3-null cohort (Figure 9B, yellow arrows). Of the bead events identified in both cohorts, we observed no significant difference in bead velocity between null and control littermates (Figure 9E and Supplemental Videos 1 and 2).

To determine that relatively equal numbers of beads were injected into the cisterna magna, we evaluated the distribution of beads at the base of the brain following euthanasia and dissection (Figure 9G). Importantly, the lateral view revealed that in the *Notch3^–/–^* brains, a large proportion of the fluorescent beads were retained in the perivascular space of the middle cerebral arteries, while this was not the case in controls (Figure 9G, yellow arrows). The pattern of retention was consistent with the tortuosity noted in the middle cerebral arteries as they ascend the lateral aspects of the brain (Figure 3, B and D). This retention explained the low number of beads found in the pial arteries (Figure 9G) of the *Notch3*-null mice but did not explain the distance of the beads from the vessels, which gave the impression that a blockade, perhaps from matrix proteins, was obstructing the flow.

While the use of fluorescent beads enabled a crude assessment of flow, a caveat to this approach was the size of the beads (1 μm), which cannot be compared with the flow of small molecules. Thus, we injected FITC-conjugated dextran in the cisterna magna of *Notch3^–/–^* and WT littermates and evaluated the course of drainage into the right ventricle (Figure 9H). Initial validation of the procedure in a large cohort (*n* = 5–7 mice per time point) indicated a reproducible, time-dependent, and quantifiable detection of fluorescence (Figure 9I). Using this approach, we observed a statistically significant reduction in the drainage of FITC dextran in the *Notch3*-null mice when compared with WT littermates (Figure 9J).

**Figure 10:**
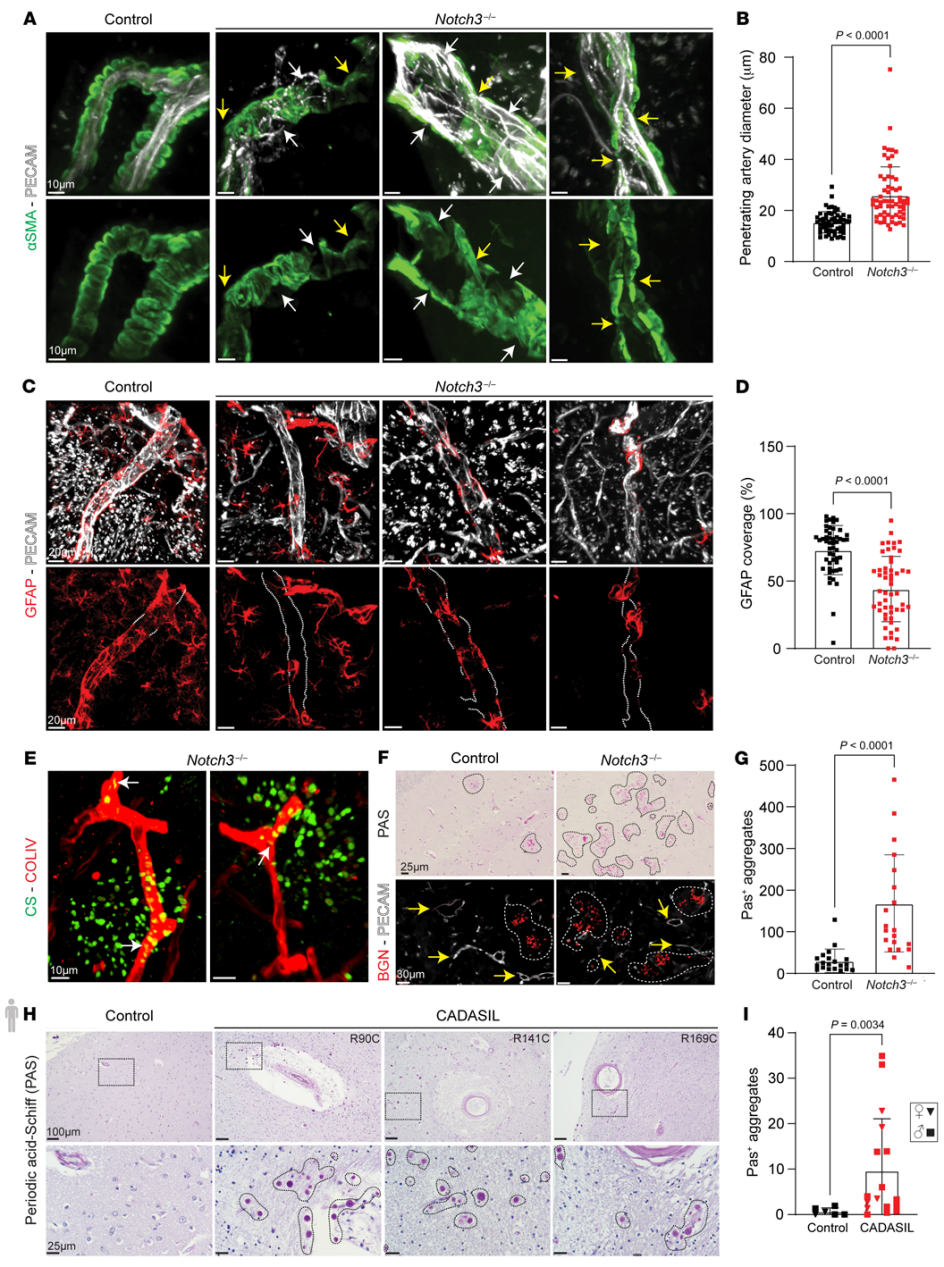
Deletion of *Notch3* results in enlargement of penetrating arteries, detachment of astrocytes, and accumulation of extracellular proteoglycans in the brain parenchyma. (**A**) Penetrating arteries of 24-month control and *Notch3^–/–^* mice stained with αSMA (green) and PECAM (white). White arrows indicate loss of VSMC coverage; yellow arrows indicate dilations and tortuosity. (**B**) Diameter of penetrating arteries. Data are shown as the mean ± SD; *n* = 10–16 vessels per animal, 5 mice per group. Mann-Whitney test. (**C**) Penetrating arteries of 24-month control and *Notch3^–/–^* mice stained with GFAP (red) and PECAM (white). White dotted lines highlight regions of poor GFAP^+^ coverage. (**D**) Percentage of vessel covered by GFAP^+^ astrocytes in control and *Notch3^–/–^*. Data are shown as the mean ± SD; *n* = 9–13 vessels per animal, 5 mice per group. Mann-Whitney test. (**E**) Vascular capillaries stained with type IV collagen (red) and chondroitin sulfate (green) in *Notch3^–/–^*. White arrows identify intracellular chondroitin sulfate. (**F**) Periodic acid–Schiff (PAS) staining in coronal sections of aged (24-month) control and *Notch3^–/–^*. Identification of biglycan (red). Yellow arrows indicate vessels; dashed white lines highlight biglycan. (**G**) Quantification of PAS^+^ aggregates in control and *Notch3^–/–^*. Data are shown as the mean ± SD; *n* = 5 visual fields per animal, 4 mice per genotype. Mann-Whitney test. (**H**) PAS staining in brain sections from control and CADASIL patients. Dotted black lines highlight PAS granules. (**I**) Average number of PAS^+^ aggregates in age-matched, mixed-sex cohort of control and CADASIL patient brain samples. Data are shown as the mean ± SD; *n* = 6–10 visual fields per individual, *n* = 6 control and 17 CADASIL patients. Mann-Whitney test. Squares, male; inverted triangles, female.

Structurally, several consistent alterations were noted in the penetrating arteries of *Notch3*-null mice. First, loss of smooth muscle cell coverage and enlargement of luminal inner diameter were regularly found by 2 years of age (Figure 10, A and B, and Supplemental Figure 13A). Upon injection of PECAM antibodies, penetrating arteries were easily identifiable in WT mice as daggers infiltrating the brain (Supplemental Figure 13A). In contrast, owing to their dilation, penetrating arteries of age-matched *Notch3^–/–^* littermates were indistinguishable from other vessels at lower magnification (Supplemental Figure 13, A and B). Furthermore, we observed detachment of astrocytes, providing further support to the alterations in the perivascular space, which was also enlarged (Figure 10, C and D, and Supplemental Figure 13, C–F).

Given the structural and functional deficiencies in the glymphatic system, we predicted that *Notch3^–/–^* animals may show increased protein accumulation in the brain parenchyma. While we were unable to detect APP or tau, there was an accumulation of chondroitin sulfate–positive material in the brain parenchyma in close proximity to capillaries. Chondroitin sulfate was also noted in intracellularly in capillaries (Figure 10E). Using periodic acid–Schiff (PAS) to detect multiple negatively charged glycosaminoglycans and proteoglycans, we found an excess of PAS^+^ granules in *Notch3*-null mice when compared with littermates. These granules also contained biglycan, indicating an accumulation of proteoglycans and glycosaminoglycans in the *Notch3^–/–^* mouse brain (Figure 10, F and G) consistent with the scRNA-Seq data. Thus, it seems that brain vessel dysfunction impairs the glymphatic system with accumulation of glycosaminoglycans in the parenchyma. This observation was also tested in brain tissue from 23 patients with CADASIL (cerebral autosomal dominant arteriopathy with subcortical infarcts and leukoencephalopathy) and controls (Supplemental Table 3). Our data show a significant increase in the frequency of PAS^+^ granules in CADASIL brains relative to matched controls (Figure 10, H and I). We also evaluated the presence of chondroitin sulfate proteoglycans of WT mice at 2 months and 24 months of age. While we did not detect chondroitin sulfate proteoglycans at 2 months, accumulation was noted in the perivascular space by 24 months (Supplemental Figure 13G). The levels of such accumulation were more pronounced in the absence of *Notch3* by 12 months (Supplemental Figure 13H).

**Figure 11:**
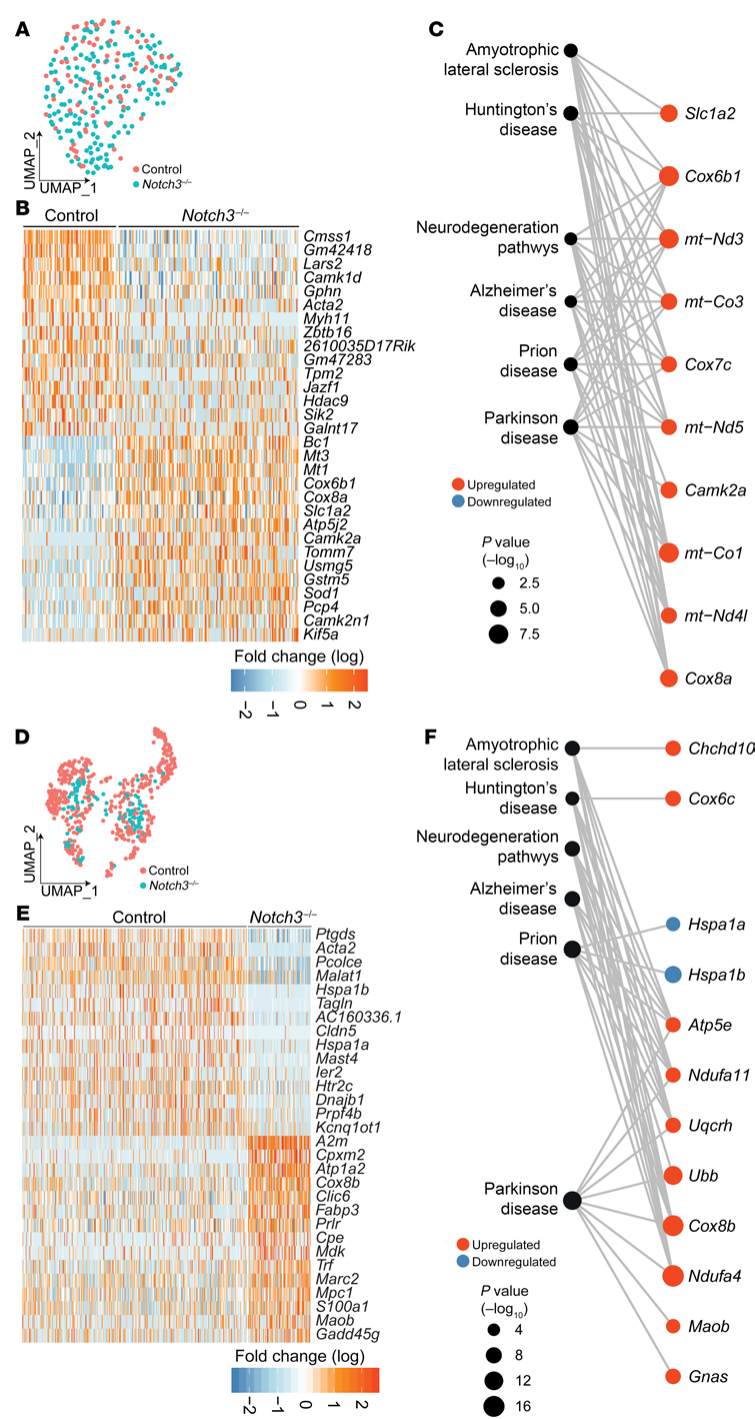
Deletion of *Notch3* leads to progressive transcriptional alterations in the neuronal compartment, revealing neurodegeneration. (**A**) UMAP plot of scRNA-Seq data from 12-month cortical neurons. (**B**) Heatmap of the top 30 DEGs in control and *Notch3^–/–^* cortical neurons. (**C**) Network of selected neurodegenerative disease–associated KEGG pathways of cortical neuron DEGs in *Notch3^–/–^*. (**D**) UMAP data from 24-month cortical neurons. (**E**) Heatmap of the top 30 DEGs in control and *Notch3^–/–^* cortical neurons at 24 months. (**F**) Network visualization of selected neurodegenerative disease–associated KEGG pathways and member genes from the top 20 enriched pathways.

Finally, scRNA-Seq evaluation of the neuronal constituency of *Notch3^–/–^* at 12 and 24 months revealed progressive alterations in transcripts associated with neuroinflammation and neurodegeneration (Figure 11, A–F). Furthermore, Kyoto Encyclopedia of Genes and Genomes (KEGG) pathway analyses highlighted multiple neurodegenerative diseases as signatures associated with *Notch3^–/–^* aged mice (Figure 11, C and F). Importantly, these transcripts were not identified in young *Notch3^–/–^* mice; instead they were preceded by alterations in metabolism (Supplemental Figure 13, I–K) that subsequently evolved into neurodegeneration. These findings underscore the effect of vascular insufficiency on neuronal function and offer a window on how age-dependent vascular dysfunction affects brain health.

## Discussion

Here, we used single-cell transcriptomics to identify age-associated molecular changes in brain vessels and found that Notch3 and Jagged1 were drastically decreased in aging, implying age-dependent reductions in Notch signaling. Indeed, we verified such reductions in human brain vessels at the protein level. Next, we used a mouse model with deletion of Notch3 to understand the resulting functional implications for the brain vasculature and the downstream consequence to the brain parenchyma. *Notch3* is essential for maintaining a differentiated state in smooth muscle cells (23, 26), which here we characterized at the single-cell transcriptome level as being associated with regulation of calcium and contractile proteins. Thus, absence of *Notch3* leads to a progressive loss of contractile function. Contractility is a critical component of the functional and structural response of the vessel wall during acute or chronic changes in blood pressure. First, we found that defective contractility and loss of VSMCs resulted in structural alterations in the microvasculature, some of these unique to the brain (microaneurysms and vascular beading). Next, we showed a progressive accumulation of extracellular matrix, which altered the perivascular space and reduced astrocyte-vascular association. The combination of impaired vascular contractility and altered structure of the perivascular space manifested as reduced glymphatic flow. Importantly, glymphatic flow was further challenged by the high production of matrix proteins with notable accumulation of glycosaminoglycans in the brain parenchyma. These pathological changes ultimately translated into a neuronal transcriptional response seen in neurodegenerative diseases with decrease in chaperones (like HSP70, as shown by reduction in *Hspa1a* and *Hspa1b* transcripts) and increases in ubiquitination and oxidative stress (NDUFA4, COX6C) particularly in a subset of cortical neurons that are susceptible to chronic stress (27). Overall, our findings provide support for the temporal sequence of events and the molecular connections that link vascular dysfunction to neuronal degeneration and identify Notch3 as a critical culprit in cerebral small vessel disease that emerges with age.

Cerebral small vessel disease is an age-dependent disorder that adversely affects brain health (28). The condition is associated with stroke and vascular dementia and frequently co-occurs with Alzheimer’s disease pathology, where it may result in accelerated and more severe neurodegeneration (29). It is estimated that over half of the elderly population exhibits radiological evidence of small vessel disease, yet a granular understanding of factors that contribute to this pathology particularly in aging has remained incomplete and its molecular regulation poorly characterized (30).

The brain imaging signatures of sporadic, age-dependent small vessel disease include white matter hyperintensities, lacunar infarcts, microhemorrhages, and dilated perivascular spaces. These findings are also prominently observed in CADASIL, a genetic disorder linked to NOTCH3 missense mutations that result in vascular dementia (31). Interestingly, several adult patients with heterozygous Notch3 stop codon mutations leading to haploinsufficiency have been diagnosed with cerebral small vessel disease (32). These and other emerging data indicate that inactivation of a single NOTCH3 allele in humans can result in a late-onset autosomal dominant small vessel disease with incomplete penetrance.

Importantly, albeit less frequent, missense biallelic null mutations in *NOTCH3* were recently identified in patients and are clinically characterized by migraines, seizures, recurrent strokes starting in early childhood, and progressive cognitive impairment (33–35). Thus, the use of *Notch3*-null mice as a proxy to understand the neurovascular consequences of vascular dysfunction is biologically relevant, and the findings presented here might shed light on disease progression.

A unifying theme between the null mouse model and the human disease is impaired smooth muscle cell contractility and altered blood flow (26, 36–39). Our scRNA-Seq data revealed a critical role of *Notch3* in the regulation of calcium dynamics and myosin light chain kinase, both absolutely required for contractility. Importantly, we also identified significant accumulation of glycosaminoglycans specifically in the Notch3-deficient model that was also found in patients with CADASIL. Recognizing the caveats of direct extrapolation from experimental models to human disease, and the existing pathological disparity between *Notch3*-transgenic mice models and CADASIL patients, these findings provide compelling evidence for neurovascular mechanisms underpinning small vessel disease–related neurodegeneration.

An important aspect of this work is the link between aging and progressive reduction in Notch3. We found that in aging, VSMCs significantly and progressively show a decline in Notch3; this finding was reproduced in human brain vessels at the protein level. We then showed molecularly and physiologically that Notch3 is responsible for maintaining vascular contractility. Combined, the results imply that NOTCH3 deficiency in aging underlies impaired vasoreactivity and vascular stiffness. The data also underscore the importance of VSMCs in regulating vascular reactivity, a process fundamental for efficient cerebral autoregulation and particularly vulnerable to age-related risk factors (40–45). It is unclear whether additional changes, such as detachment of astrocytes, might be a direct or indirect consequence of NOTCH3 deficiencies. However, the pathway is primed to coordinate cell-cell communication and function. Importantly, loss of *Notch3* has been previously associated with impaired perivascular macrophage recruitment (46). This outcome negatively affects vascular health, which relies on macrophages, and alters the immune compartment of the brain with unclear consequences during the aging process.

Epidemiological and clinical data have established that age-related cerebral small vessel disease, manifested as white matter hyperintensities on brain MRI, is associated with neurovascular dysfunction as well as age-related motor and cognitive decline (8, 12, 47–49). The structural and functional changes seen in the perivascular space, which result in impaired clearance via the glymphatic system, provide intriguing evidence for one possible mechanism whereby vascular risk factors may directly result in the accumulation of aberrant proteins (i.e., proteinopathies, and here we include glycosaminoglycans) in age-related neurodegenerative diseases (50–52).

## Methods

### Mice

B6;129S1-*Notch3*^tm1^Grid/J (*Notch3^–/–^*) (JAX:010547) and C57BL/6J (JAX:000664) mice were purchased from The Jackson Laboratory. Animals were housed at UCLA, Northwestern Medicine, and National Heart, Lung, and Blood Institute facilities.

### Human brain tissue

Formalin-fixed frontal lobe sections were acquired from the Alzheimer’s Disease Research Core at the University of Michigan and the Northwestern Nervous System Tumor Bank. Detailed characteristics including age, sex, and *NOTCH3* mutation are described in Supplemental Table 3.

### Immunostaining

#### Tissue sections.

Paraffin-embedded blocks were sectioned at 5 μm, and antigen retrieval was performed in either citrate buffer (pH 6) or Tris-EDTA buffer (pH 9) followed by primary and secondary antibody incubations.

#### Vibratomed slices.

Fixed tissue samples were vibratomed at 100 μm and blocked for 48 hours, followed by incubation with primary antibodies for 48–72 hours (4°C) and secondary antibodies overnight (4°C).

#### Fixed cells.

Cells were fixed with 2% PFA or methanol for 10 minutes followed by permeabilization for 2 hours, and incubated with primary antibodies overnight and secondary antibodies for 2 hours. Information on antibodies can be found in Supplemental Methods. Images were collected with the Nikon A1R confocal system with NIS Elements acquisition software and analyzed with Imaris software (9.9.0, Bitplane). A subset of samples were imaged using a Nanozoom RS digital slide scanner (Hamamatsu) (H&E) and analyzed with the companion NDP Viewer software or the Nikon Eclipse light microscope using a ×10 air objective (PAS). A list of antibodies can be found in Supplemental Methods.

### Vascular casting of mouse brains

Animals were injected with heparin (1 U/g, i.p.) and then euthanized. A catheter prefilled with 10^−4^ M sodium nitroprusside (SNP) in PBS was introduced into the thoracic aorta and secured in place with 2 sutures. SNP/PBS was perfused to both maximally dilate the vasculature and remove all blood; then Microfil (Flowtech Inc.) was injected at an 8:1:1 ratio (polymer/diluent/curing agent) until the distal vasculature of the brain was filled (53). Brains were dissected and placed in 10% buffered formalin.

### Micro-CT scan and parameters and reconstruction

Brain samples were scanned on a Bruker Skyscan 1276 system with voltage 50 kV, current 100 μA, and a 0.25 mm Al filter. Image capture was set with a pixel size of 6 μm. Per scan, we acquired an average of 2,100 projection images at a 0.2° rotation step over 360° total to improve the signal-to-noise ratio. Reconstruction of the data was generated with NRecon software (Bruker) for .bmp files for downstream processing by Imaris (9.7.2, Bitplane). Additional information on visualization and quantitative analysis is provided in Supplemental Methods.

### Retinal VSMC coverage analysis

Quantification of retinal VSMC coverage of arterial vessels was performed using the NIS Elements software to calculate the relative percentage of αSMA^+^ area. Additional details and imaging parameters are provided in Supplemental Methods.

### Brain vessel isolation and single-cell sequencing

*Notch3^–/–^* and control littermates were euthanized at 1, 12, and 24 months. After sacrifice, mice were perfused with versene, and brains were dissected in PBS. Using a microscope, meninges and penetrating vasculature were carefully dissected from the brain and incubated in 500 μL of digestion solution for 25 minutes at 37°C on an orbital shaker while pipetting up and down every 2–3 minutes to mix the solution and generate a single-cell suspension. Detailed information on suspension cocktail components as well as postdigestion clean-up prior to cell counting and viability assessment is given in Supplemental Methods. Libraries were prepared using 10× Genomics Chromium Single Cell 3′ Library & Gel Bead Kit v3 per the manufacturer’s protocol. For the generation of single-cell gel beads in emulsion, cells were loaded onto the Chromium single-cell instrument (10X Genomics) with an estimated targeted cell recovery of approximately 5,000 cells. Sequencing was performed on an Illumina Novaseq 6000. Libraries were processed using the Cell Ranger pipeline (10X Genomics) and analyzed using the R package Seurat (54). The following R packages were used for data visualization in combination with Seurat: ggplot2, ggraph, igraph, and tidyverse.

### Vascular smooth muscle culture and contraction assays

A portion of the descending aorta was dissected into small fragments (1 mm^3^). Explants were plated in DMEM + 10% FBS. Cells were allowed to grow and trypsinized only after 1 week for subculture. Differential plating (5 minutes) was performed to separate fast-attaching cells (mostly fibroblasts) from VSMCs. The purity of the VSMC culture was confirmed by immunodetection of αSMA. To assess vascular smooth muscle contractility, a neutralized solution of collagen I was mixed with VSMC cell suspension and plated into 6-well plates. Gels were allowed to polymerize for 2 hours, and then medium was added to the wells. A p200 pipette tip was used to detach the gels from the lateral aspects of the wells. Gels were returned to the incubator for 24 hours and then fixed in 2% PFA for imaging and staining.

### Western blotting

Lysates from aortic VSMCs were prepared in 8 M urea and run under denatured conditions on an SDS-PAGE 4%–12% gradient gel, transferred onto nitrocellulose membranes, and blocked and incubated overnight at 4°C with primary antibodies. Immunocomplex detection was performed with the enhanced chemiluminescence SuperSignal West Femto Maximum Sensitivity Substrate (Thermo Fisher Scientific) and SuperSignal West Pico Maximum Sensitivity Substrate (Thermo Fisher Scientific) using the ChemiDoc XRS+ Molecular Imager (Bio-Rad). Densitometry analysis was performed using ImageLab Software. Information on antibodies used is available in Supplemental Methods.

### In vivo hemodynamics and vascular reactivity

Under anesthesia, a pressure catheter (1.0F, model SPR1000, Millar Instruments) was introduced into the right carotid artery and advanced to the ascending aorta of the animal. Each animal was allowed to acclimate for 5 minutes, at which time isoflurane was reduced to 1%. Pressures were recorded using Chart 8 software (ADInstruments) and analyzed from 1 to 4 minutes.

For reactivity, a central venous catheter (Instech, PE-10 tubing) was placed in the jugular vein; in addition, an arterial pressure catheter (Millar Instruments) was used to measure continuous blood pressure (BP) and heart rate. Baseline BP was established, and mice were then injected with increasing bolus concentrations (10 μg/kg and 100 μg/kg) of phenylephrine or increasing bolus concentrations (10 μg/kg and 100 μg/kg) of acetylcholine diluted in PBS. BP and heart rate were monitored throughout the procedure, and the animal’s BP was allowed to return to baseline before administration of the next dose. Response to vasoactive drugs was calculated as absolute maximal deviation of systolic BP from baseline after drug administration(s) before return to baseline.

### MRI perfusion and angiography

MRI experiments were conducted using a 7T ClinScan MRI (Bruker) and dedicated mouse brain coils. Details of image acquisitions, processing, and analysis are given in Supplemental Methods.

### Surgical cisterna magna cannulation and thinned-skull preparation

Mice were anesthetized with ketamine/xylazine at 100 mg/kg and 10 mg/kg, respectively, and placed in a stereotactic frame under a heating pad. A midline incision was made across the scalp, and the skin and periosteum were removed to expose the skull surface. Artificial cerebral spinal fluid at 37°C was applied to the exposed skull surface, and a high-speed drill was used to thin 2 circular areas of the skull (~1–1.5 mm in diameter) lateral to the superior sagittal suture between the bregma and lambda sutures anteriorly and posteriorly, respectively, corresponding to the cortical middle cerebral artery surface bilaterally (55). Cisterna magna cannulation was performed by tilting of the animal’s head forward within the stereotactic frame to expose the neck muscles and occipital crest. The overlying skin and dorsal neck muscles were separated with forceps along the midline, exposing the cisterna magna. A 30G sterile needle was filled and inserted at 45° at the center of the cisterna magna at a depth of approximately 1 mm. The needle tip was secured in place using cyanoacrylate glue with accelerator on the adjacent dural membrane. Once secured, the tubing was cut and sealed. A thin silicone ring was affixed to the exposed skull at the margins of the skin using Vetbond tissue adhesive to create a well for artificial cerebrospinal fluid (aCSF) during intravital imaging. Thirty minutes before initiation of intravital imaging, mice underwent retro-orbital injection with non-blocking rat anti–mouse PECAM-1 antibody for blood vessel labeling conjugated to DyLight-650.

### Intravital imaging of perivascular flow

Imaging was performed using an Olympus BX-51WI Fixed Stage illuminator with a Yokogawa CSU-X1-A1 spinning disk, a Hamamatsu EMCCD C9100-50 camera, and a Modular Laser System with solid-state diode lasers with DPPS modules for 488, 561, and 640 nm and the appropriate filters as previously described (56). Synchronization was managed by a Prosync 2 Controller. *Z* axis movement and objective positioning were controlled by a Piezoelectric MIPOS100 System. Fifteen minutes before intravital imaging, green fluorescent polystyrene microspheres (FluoSpheres, 1.0 μm, 505/515 nm, 0.4% solids in aCSF) were briefly sonicated and infused at 2 μL/min for 5 minutes through the cannula using an infusion pump. Images were collected with a ×20 water immersion objective using Volocity software. No UV light/excitation was used for this method. Images from the Volocity software were transferred to Imaris. The largest artery in each visual field was measured along the widest axis for diameter analysis. Aneurysms were counted per vessel from 2–6 vessels per animal with a total of 5–8 animals per genotype quantified.

### Image analysis and fluorescent bead tracking

Images were analyzed using ImageJ software. Fluorescent microsphere tracking was performed using individually acquired 1-minute single-channel recordings obtained across multiple intravital imaging sessions. Microsphere flow speed was measured by tracking of total perivascular distance traveled over time within a given series of frames and averaging of flow speed from 3 separate observed particles per recording.

### Dextran clearance experiments

FITC-conjugated 70 kDa dextran (5 μL at 1% in aCSF) was infused into the cisterna magna of a mixed-sex cohort of 3-month-old C57BL/6J mice. Animals were euthanized from 5 to 180 minutes after injection, and 400 μL of blood was collected using cardiac puncture. Blood was mixed with 10% citrate buffer to obtain plasma, which was evaluated using a Synergy LX plate reader. To assess changes in early clearance in *Notch3^–/–^* mice and controls, the sample process was applied and plasma was measured 5 minutes after injection.

### Quantification of diameter of penetrating arteries and perivascular space

One-hundred-micrometer coronal sections injected with PECAM-1–647 were scanned using a Nikon A1R confocal microscope. In Imaris, 3D renderings of the cerebral vasculature were generated and diameter measured at 100 μm from the meninges into the cortex. A total of 10–16 vessels were measured per mouse, 5 mice per genotype quantified. To measure perivascular space, transversal sections stained with aquaporin 4 antibodies along with DAPI were scanned using a ×40 objective and images transferred into Imaris. For each vessel, the smallest distance was measured from the top of DAPI^+^ nuclei to the edge of the aquaporin 4 staining. Nine to sixteen vessels were quantified per mouse, 3 mice per genotype.

### Quantification of GFAP coverage in penetrating arteries

Two-hundred-micrometer coronal sections were stained with PECAM-1 and GFAP antibodies and scanned using a Nikon A1R confocal microscope. Images were processed using NIS Elements. In Imaris, surface feature was used to generate 3D renderings of the cerebral vasculature. For each vessel, 2 measurements across the total length of the vessel were generated using measurement tools and then averaged to obtain percentage of vessel length covered by GFAP^+^ astrocytes. Nine to thirteen vessels were measured per animal, 5 animals per genotype.

### Identification and quantification of PAS aggregates

Five-micrometer paraffin-embedded coronal sections were stained with periodic acid–Schiff (PAS). Images were collected with Nikon Eclipse light microscope using ×10 air objective. Images measured 1,378.25 total μm area. Five images per field of view were captured per animal and quantified using NIS Elements software spot quantification macro.

### Statistics

For non-transcriptomic data sets, data were analyzed using GraphPad Prism version 10.0 for Windows. Two-tailed unpaired *t* test was used for data with a Gaussian distribution and equal variance. Two-tailed unpaired *t* test with Welch’s correction was used for data with a Gaussian distribution and unequal variance. Alternatively, data with non-parametric distributions were analyzed by Mann-Whitney *U* test. *P* values less than 0.05 were regarded as statistically significant. For multiple conditions, the Kruskal-Wallis test was used with non-parametric distributions with multiple testing corrections. All data are presented as the mean ± SD, unless noted. For scRNA-Seq the R package Seurat (version 3.0.2) was used. The FindAllMarkers Seurat function was used to identify cluster markers for each cell population; the default Wilcoxon’s rank-sum test was used to calculate statistical significance, and genes were filtered using adjusted *P* value ≤ 0.05. Pathway enrichment analysis was performed with Metascape, which uses hypergeometric distribution in the calculation of significance for each gene enrichment category. For KEGG pathway analysis, the Database for Annotation, Visualization and Integrated Discovery (DAVID) was used.

### Study approval

For all animal studies, protocols and experimental procedures were previously reviewed and approved by the Institutional Animal Care and Use Committees of UCLA, Northwestern University, and the NIH. These protocols were conducted in accordance with federal regulations as outlined in the *Guide for the Care and Use of Laboratory Animals* (National Academies Press, 2011). For human studies, samples were collected and processed in accordance with the standards for human research set by the University of Michigan and Northwestern University and in accordance with Declaration of Helsinki principles.

### Data availability

All scRNA-Seq data sets were deposited in the NCBI’s Gene Expression Omnibus database (GEO GSE204803), and values for all data points in graphs are reported in the Supporting Data Values file.

## Author contributions

MCR designed and performed experiments and wrote and edited the manuscript. FM performed bioinformatics analysis. RHK, AM, GEH, JS, SM, AB, DPS, DP, SB, EK, AS, JW, and CH performed experiments. KM coordinated patient sample distribution. AMS provided human patient samples and supervised experiments. MMW and FAS provided human patient samples and intellectual input. ETL, EAF, MB, WAM, and BAK provided intellectual discussion. MLIA conceived the study, designed experiments, and wrote and edited the manuscript. All authors had the opportunity to comment on the final manuscript.

## Supplementary Material

Supplemental data

Unedited blot and gel images

Supplemental table 1

Supplemental table 2

Supplemental table 3

Supplemental video 1

Supplemental video 2

Supporting data values

## Version 1 11/28/2023

In-Press Preview

## Version 2 01/16/2024

Electronic publication
