# Supplementary material for: Age-related loss of Notch3 underlies brain vascular contractility deficiencies, glymphatic dysfunction, and neurodegeneration in mice
Source: J Clin Invest. 2024 Jan 16;134(2):e166134. doi: 10.1172/JCI166134 (PMC10786701; doi:10.1172/JCI166134)

Supplemental Materials

Age-related loss of Notch3 underlies brain vascular contractility deficiencies, glymphatic dysfunction, and neurodegeneration in mice.

Authors: Milagros C. Romay, Russell H. Knutsen, Feiyang Ma, Ana Mompeón, Gloria E. Hernandez, Jocelynda Salvador, Snezana Mirkov, Ayush Batra, David P. Sullivan, Daniele Procissi, Samuel Buchanan, Elise Kronquist, Elisa A. Ferrante, William A. Muller, Jordain Walshon, Alicia Steffens, Kathleen McCortney, Craig Horbinski, Elisabeth Tournier-Lasserve, Adam M. Sonabend, Farzaneh A. Sorond, Michael M. Wang, Manfred Boehm, Beth A. Kozel, and M. Luisa Iruela-Arispe

This file contains the following:
Supplemental Methods
Supplemental References
Supplemental Figures 1-13

Supplemental Methods.

Immunohistochemistry: Associated Reagents and Materials. For tissue fixation, samples were collected and fixed in 2% PFA in 1X PBS (Fisher Scientific, AAJ61899AP) overnight at 4 °C with gentle agitation. For tissue permeabilization, blocking and antibody staining samples were treated with a solution containing 5% normal donkey serum (Jackson Immuno Research Laboratories #017-000-121) with 0.1% Triton X-100 (Fisher Scientific, BP151500) 0.025% Tween 20 (Fisher Scientific, BP337500) in 1X HBSS for 1 hour (paraffin sections) or 24 - 48 hours prior to immunostaining (whole mount/vibratome). In addition to antibodies listed above, DAPI (Fisher Scientific, D1306) was used to visualize nuclei when indicated. In the specific case of human brain sections, Ulex Europaeus Agglutinin I (Vector Laboratories, DL-1067), was used to identify endothelial cells when indicated. For vibratome sections, samples were mounted on glass slides supported with SS1X20-SecureSeal Imaging Spacers (Grace Bio-Labs, 654006). For all staining samples, Prolong Gold (Thermo Fisher Scientific, P36930) was used as the mounting media. For CD31 staining with DIANOVA DIA-310, tyramide amplification was performed using the Alexa Fluor 647 Tyramide SuperBoost Kit (Thermo Fisher Scientific, B40936).

Middle Cerebral Artery (MCA) Histology and Analysis. Aged *Notch3*$^{-/-}$ and matched control mice were sacrificed and perfused with 1X PBS to flush the blood from the vasculature. The brain was then removed from the skull and a 2mm trimmed segment of MCA (emanating off the Circle of Willis), and surrounding neural tissue was then excised and placed in 10% buffered formalin overnight. The tissue samples were then transferred to 70% ethanol, paraffin embedded, and serially sectioned at 5 µm. Sections were subsequently stained for hematoxylin and eosin (H&E) and vessel circumference measured. Stained slides were then scanned using a Nanozoom RS digital slide scanner (Hamamatsu) and analyzed utilizing the companion NDP Viewer software (Hamamatsu Photonics, Hamamatsu, Japan). Statistical analysis across genotypes was performed using GraphPad Prism version 9.0 for Windows (GraphPad Software, La Jolla California, USA).

MCA Volumetric and Tortuosity Quantification. To assess volumetric changes in the MCA of control and *Notch3*$^{-/-}$ animals, surface volume renderings of the vessel castings were generated using the grayscale channel of the micro-CT data using the surface render feature with background subtraction. Once the surface was rendered, a reference frame was set using set reference frame feature to manually set axis of the posterior distance of the brain (moving from the Circle of Willis upwards towards top of the brain) as the Z axis. Measurement of the volumetric space of the MCA was performed by setting a reference distance covering 3000µm in Z from the start of the MCA at the branch point from the Circle of Willis. In this 3000µm set reference distance, all surface components of the MCA surface rendering generated within the region were merged into one large surface using the unify feature. This large surface was then quantified using the volume statistics feature in Imaris. To color code the volumetric data, the individual MCA volumes were duplicated into a new surface and overlayed onto the grayscale micro-CT channel visualized in white. Color coding of volume size was set using the statics coloring feature

in the duplicated surface by using lowest MCA volume calculated as a bottom reference volume and largest MCA volume as top reference. Tortuosity index of the MCA was calculated by measuring the path length of the MCA (including vascular tortuosity) relative to the 3000 µm boundary in Z from the MCA (the Euclidean distance)(1, 2). Statistical significance of the MCA volumetric comparisons and tortuosity index across genotypes was performed using GraphPad Prism version 9.0 for Windows (GraphPad Software, La Jolla California, USA).

Tortuosity (TR) and Vascular Dilation Rating (VDR)
Post vascular casting, brains were placed under a dissecting microscope and qualitatively assessed/scored for both MCA tortuosity and global vascular pathology/dilations. Two blinded raters independently scored each sample on a scale of 0-3 where, 0 is no pathology, 1 mild, 2 moderate, and 3 severe tortuosity or vascular dilations. Scores were then assessed for inter-rater reliability. If 2 values for a given measure deviated by more than 1 then the samples were reevaluated. If the new values fell within 1 then the scores were updated and kept. If the deviation remained, then the values were omitted from analysis. The remaining scores were then averaged between raters and reported.

Retinal vSMC coverage analysis. To quantify vascular smooth muscle cell coverage of arterial vessels, aged match control and *Notch3*$^{-/-}$ retina were stained with for anti-alpha smooth muscle actin (αSMA, to identify vSMCs) and PECAM (to identify endothelium) and imaged using the Nikon A1R confocal system with the NIS Elements acquisition software using a 10X objective and both Z-stack and tile scan features enabled to capture the entirety of the retinal vasculature in one image. The resultant large image file was then pre-processed in the NIS elements software using the following options: stitch, maximum intensity projection and denoise to generate the 2D image used for quantification. In the 2D image, each of the primary arterial vessels were defined as regions of interest (ROI) by tracing the arterial branch from to optic nerve to the retinal leaf edge, excluding subsequent lower-order branches. The total number of fluorescent αSMA pixels present in the ROI is quantified and then subtracted from the total number of pixels present in the ROI. The resultant number is then subtracted by 1 and divided by the total pixel number to calculate the relative percentage of αSMA + area in the vessel branch. This analysis is then repeated across all arterial branches and averaged to generate the relative αSMA + area per each retina. Statistical significance of the αSMA + area was performed using GraphPad Prism version 9.0 for Windows (GraphPad Software, La Jolla California, USA).

Digestion solution for brain single cell suspensions. For preparing tissue samples for single cell sequencing 500 of cell dissociation solution composed of: DNase I (Qiagen, 79254), 10mM HEPES (MT25060CI, Corning) and Liberase TH (Sigma-Aldrich, 5401135001, 70ug/ml) in 1X HBSS.

Post-digestion sample clean-up for single cell suspensions. Following cell digestion, Cell suspensions were centrifuged at 200xg for 3 min and supernatants were removed. 500ul of pre-warmed 1X trypsin per sample was added to each pellet and resuspended for 5 min. Cell suspensions were then transferred to 15ml tubes containing 6ml of DMEM 10% FBS to neutralize the enzyme digestion solution and filtered through 30um cell strainers (Miltenyi Biotec, 130-041-

407) to remove undigested tissue debris. Filtered cell suspensions were centrifuged at 200xg for 3 min and cell pellets were washed adding 10ml 1X DPBS. After centrifugation at 200xg for 3 min and removal of the supernatants, pellets were washed in 8ml DPBS 0.04% ultrapure BSA (Thermo Scientific, AM2618) and centrifuged again (200xg for 3 min). After supernatant removal, the final cell pellets were resuspended in 30-50ul DPBS 0.04% ultrapure BSA for cell counting and viability assessment before loading the Chromium Next GEM Chip G.

Preparation of aortic enriched lysates for western blots. Aortic vSMC enriched lysates were prepared from descending aortic fragments. Animals were euthanized in a CO_2 chamber following institutional guidelines. Following sacrifice, the animals were perfused with ice cold 1X PBS in the left ventricle using a 12mL syringe with 22G needle to remove blood prior to aortic vessel isolation. Following perfusion, the thoracic aorta was dissected from the animal by cutting the vessel from the first intercoastal artery to the diaphragm for protein lysis. Once isolated, the adventitial fat was removed from aortic fragment and the fragment was flushed with 100 µL of 8M urea using a 1mL syringe with a 30G blunt needle inserted into the vessel lumen to remove endothelial cell protein. Following the luminal urea flush, the aortic fragment was diced, and the resultant tissue pieces were manually disrupted using a disposable pellet pestle (Fisher Scientific 12-141-364) in 1.5mL tube with 100 µL of 8M urea. The resultant vessel slurry was left on ice for 20 minutes and vortexed every 5 minutes to assist tissue digestion. The resultant vessel lysate was then centrifuged at 10,000 x g at 4°C for 15 minutes to separate the cytosolic protein from the remaining extracellular matrix.

MRI perfusion and angiography. All MRI experiments were conducted using a 7T CLinScan MRI (Bruker, Germany) and dedicated mouse brain coils. After insertion of tail-vein catheter for delivery of gadolinium contrast, each mouse was anesthetized and placed in dedicated holder with body temperature recorded and maintained through warm circulating water. Respiration was monitored using a pneumatic pillow placed under the chest of each mouse. Signals were recorded using the dedicated physiological monitoring software (SAI instruments). The following sequences were used to acquire images depicting whole brain anatomy and angiography, brain regional R2* parametric values during Air and Oxygen gas inhalation and brain perfusion data maps following injection of contrast agent (Prohance, Bracco): 1) Angiography: multislice time-of-flight flow sensitive sequences with TR=24 msec, TE=5msec, in plane spatial resolution ~ 30µm and slice thickness ~0.2 mm; 2) R2* maps during gas challenge: multiple gradient echo sequence was used to acquire images with different echo times (n=8 TE values ranging from 2msec to 24 msec) and a TR=100msec with FA=15. The sequence was repeated 12 times with a scheme which involved administering isoflurane anesthesia with medical air (4 repetitions) followed by isoflurane plus 100% oxygen (4 repetitions) and then back to air; 3) sequential (n=50 repetitions) 2D multi-slice gradient echo sequence TR=20 msec and TE=2 msec FA=15. Spatial resolution ~150 µm, Slice thickness 1 mm. Whole-brain coverage was achieved for all scanned mice. Gadolinium contrast was administered during dynamic acquisition of T2* weighted imaging at a dose of 0.5mmol/Kg of PROHANCE. Following acquisition, DICOM image were exported off-line and processed using different software packages (ITK-SNAP, JIM7.0 XInapse). 3D rendered angiography patterns was generated using an automated threshold-based segmentation algorithm included in ITK-SNAP. The resulting segmented regions was transformed into STL

format and visualized using MeshLab2022 software. Whole brain R2* maps with mouse breathing isoflurane plus air and isoflurane plus oxygen were generate using the built-in voxel by voxel least square fitting tool in JIM7.0. Quantitative whole brain analysis was done through segmentation of mice brain and extraction of whole brain R2* values for each of the mice scanned and then after averaging of the obtained values for each cohort. Relative changes from baseline (AIR pre-oxygen) was recorded and compared between the two groups. Dynamic contrast enhanced sequences was processed using built-in perfusion analysis tool of Jim7.0. The resulting parametric maps from mice across groups was compared to determine differences in relevant parameters (cerebral blood perfusion, Cerebral blood volume and mean transit time). Relative AIR-Oxygen-AIR changes in R2* were compared across cohorts.

Detailed Antibody Information.

Usage	Antibody Name	Vendor	Catalog	Dilution
Immunostaining - Paraffin	Anti-smooth muscle actin (αSMA)	Sigma-Aldrich	F3777	1:750
Immunostaining – Paraffin	Notch3	Abcam	ab23426	1:200
Immunostaining – Paraffin	Mouse/Rat CD31	DIANOVA	DIA-310	1:50
Immunostaining – Paraffin	Collagen IV	Abcam	ab6586	1:200
Immunostaining – Paraffin	Anti-Fibronectin	Abcam	ab2413	1:50
Immunostaining – Paraffin	Phosphorylated Myosin Light Chain 2	Cell Signaling Technology	3671S	1:200
Immunostaining – Paraffin	Anti-Phospholamban	abcam	ab219626	1:100
Immunostaining – Paraffin	Anti-CCN3(Nov)	abcam	ab137677	1:50
Immunostaining – Paraffin	Thymosin-beta 4 (Tmsb4x)	Proteintech,	19850-1-AP,	1:200
Immunostaining – Paraffin	Anti-Superoxide Dismutase 3	abcam	ab21974	1:200
Immunostaining – Paraffin	Anti-Sulfatase 1	abcam	ab32763	1:200
Immunostaining – Paraffin	Anti-Biglycan	Kerafast,	ENH020-FP,	1:200
Immunostaining – Paraffin	Donkey – Anti Rabbit – Alexa-568	Thermo Fisher Scientific	A10042	1:500
Immunostaining –	anti-smooth muscle actin (αSMA)	Sigma-Aldrich	F3777	1:750

Vibratome/Whole Mount				
Immunostaining – Vibratome/Whole Mount	Mouse/Rat CD31	R and D Systems	AF3628	1:500
Immunostaining – Vibratome/Whole Mount	Anti-PECAM1 Antibody, clone 390	EDM Millipore	CBL1337	25ul of 1.2µg/mL, intra-occular injection 5 minutes prior to sacrifice.
Immunostaining – Vibratome/Whole Mount	Anti-Calponin 1	Abcam	ab46794	1:200
Immunostaining – Vibratome/Whole Mount	Anti-MYH11	Sigma-Aldrich	HPA015310-100U	1:200
Immunostaining – Vibratome/Whole Mount	Anti-Tagln/Transgelin	abcam	ab14106	1:200
Immunostaining – Vibratome/Whole Mount	Anti-Water Channel Aquaporin 4	Millipore Sigma	A59971	1:500
Immunostaining – Vibratome/Whole Mount	Anti-GFAP	Proteintech	, 16825-1-AP	1:500
Immunostaining – Vibratome/Whole Mount	Monoclonal anti-chondrotin sulfate CS-56	Millipore Sigma	C8035	1:400
Immunostaining – Vibratome/Whole Mount	Donkey – Anti Rabbit – Alexa-568	Thermo Fisher Scientific	A10042	1:500

Immunostaining – Vibratome/Whole Mount	Goat – Anti Mouse IgM – Alexa-488	Thermo Fisher Scientific	A21042	1:500
Immunostaining – Vibratome/Whole Mount	Donkey – Anti Goat– Alexa-488	Thermo Fisher Scientific	A11057	1:500
Western Blot	Phosphorylated-Myosin Light Chain 2	Cell Signaling	3671S	1:1000
Western Blot	Myosin Light Chain 2	Cell Signaling	8505S	1:1000
Western Blot	Anti-Lamin A	Abcam	ab26300	1:1000
Western Blot	Anti-Calponin 1	Abcam	ab46794	1:5000

SUPPLEMENTAL REFERENCES

1. Ciurica S, Lopez-Sublet M, Loeys BL, Radhouani I, Natarajan N, Vikkula M, Maas A, Adlam D, and Persu A. Arterial Tortuosity. *Hypertension.* 2019;73(5):951-60.
2. Knutsen RH, Gober LM, Kronquist EK, Kaur M, Donahue DR, Springer D, Yu ZX, Chen MY, Fu YP, Choobdar F, et al. Elastin Insufficiency Confers Proximal and Distal Pulmonary Vasculopathy in Mice, Partially Remedied by the KATP Channel Opener Minoxidil: Considerations and Cautions for the Treatment of People With Williams-Beuren Syndrome. *Front Cardiovasc Med.* 2022;9(886813.

SUPPLEMENTAL FIGURE LEGENDS

Supplemental Figure 1. Quality control and characterization of young (1 month) and aged (24 month) scRNA-seq datasets. (A) Violin plots visualizing the number of identified transcripts (nFeature_RNA) and distribution of average transcriptional expression (nCount_RNA) by genotype in the scRNA-seq data. **(B)** UMAP plot of scRNA-seq data visualizing spread of all cells by genotype. **(C)** Violin plots visualizing the number of identified transcripts and distribution of average transcriptional expression by cluster in the scRNA-seq data. **(D)** UMAP plot of scRNA-seq data visualizing spread of data by identified clusters. **(E)** Distribution of cells in each cluster by genotype as percentage. **(F)** Dot plot visualizing the expression of classical marker genes used to identify cell types in the young and aged scRNA-seq data (EC = endothelial cell). **(G)** UMAP plot of scRNA-seq data visualizing spread of all cells by cell type. **(H)** Violin plots visualizing the expression of I) classical vascular smooth muscle markers, II) Notch Receptors, III) Notch Ligands and IV) Canonical Notch target genes in the vascular cell types (smooth muscle, pericyte, arterial, capillary and venous endothelium).

Supplemental Figure 2. Accelerated age-dependent loss of vSMCs in the retinal arteries of *Notch3*$^{-/-}$ mice. (A) Schematic illustrating quantification of vSMC vascular coverage. Following imaging of retinal vasculature, individual arteries were skeletonized and αSMA staining was

quantified and used to calculate vSMC coverage per arterial branch. **(B)** Representative images of retinal vasculature stained with αSMA (green) to identify smooth muscle in combination with PECAM (red) for endothelium at the indicated time points from a mixed sex, aged-match cohort of control and *Notch3*$^{-/-}$ mice. White arrows indicate gaps in arterial vSMC coverage along the artery. Quantification of vSMC coverage across multiple retinas per time point from sex mixed cohorts. **(C)** Quantification of vSMC coverage with progressive age in *Notch3*$^{-/-}$ and littermate control mice. Error bars=mean± SD, n=4-6, per timepoint and genotype. P-value calculated using Welch's t-test (2 weeks, 4 weeks, 52 weeks and 104 weeks) or Mann-Whitney (8 weeks and 26 weeks). **(D)** Representative images of higher order branches of retinal arterial vessels from aged (104 weeks) control and *Notch3*$^{-/-}$ mice stained with αSMA (green) to identify smooth muscle in combination with PECAM (red) for endothelium. Note loss of vascular smooth muscle coverage in branches of the arterial tree in *Notch3*$^{-/-}$ animals compared to the primary artery.

Supplemental Figure 3. Absence of Notch3 results in progressive vSMC loss, disorganization, and de-differentiation. (A) Arterial tree in the mouse brain. **(B)** Representative staining of indicated blood vessels in **A** immunolabeled with αSMA (green) in control and *Notch3*$^{-/-}$ animals at 2 months (n=15, 7 control (3F/4M), and 8 *Notch3*$^{-/-}$ (5M/3F)). White arrows=lack of vSMC coverage. **(C)** H&E staining of the middle cerebral artery (MCA) 18 month male control and *Notch3*$^{-/-}$ animals. **(D)** Quantification of MCA diameter. Error bars=mean±SD, n= 3-5. Unpaired Student's t-test. **(E)** Immunofluorescence of indicated vSMC markers [Calponin(*Cnn1*), Myosin Heavy Chain 1(*Myh11*), Transgelin(*Tagln*)] in red and alpha-SMA (green) from pial brain arteries of mixed sex at 2 months. White arrows highlight loss of vSMC marker expression. Yellow arrows highlight tortuosity.

Supplemental Figure 4. Quality control and characterization of mature (12 month) scRNA-seq datasets. (A) Violin plots visualizing the number of identified transcripts (nFeature_RNA) and distribution of average transcriptional expression (nCount_RNA) by genotype in the scRNA-seq data. **(B)** UMAP plot of scRNA-seq data visualizing spread of all cells by genotype. **(C)** Violin plots visualizing the number of identified transcripts and distribution of average transcriptional expression by cluster in the scRNA-seq data. **(D)** UMAP plot of scRNA-seq data visualizing spread of data by identified clusters. **(E)** Distribution of cells in each cluster by genotype as percentage. **(F)** Dot plot visualizing the expression of classical marker genes used to identify cell types in the scRNA-seq data,(EC - endothelial cell). **(G)** UMAP plot of scRNA-seq data visualizing spread of all cells by cell type. (H) Violin plots visualizing the expression of I) classical vascular smooth muscle markers, II) Notch Receptors, III) Notch Ligands and IV) Canonical Notch target genes in the vascular cell types (smooth muscle, pericyte, and endothelium).

Supplemental Figure 5. Sex does not impact the top 50 differential expressed genes (DEG) emerging from loss of *Notch3*. (A) Graphical representation of the experimental design used to identify the role of sex in modulating DEG in the context of Notch3 loss. vSMCs from the 12 month comparison between *Notch3*$^{-/-}$ and littermate control animals (group I) were isolated and segregated into female vSMCs or male vSMCs based on the expression of pattern of 8 genes from either the X (*Xist*) or Y chromosome (*Ddx3y, Eif2s3y, Kdm5d, Uba1y, Usp8y* and *Zfy*). Once sex was determined, pairwise comparisons between female *Notch3*$^{-/-}$ and control (group II) as well

as male *Notch3*$^{-/-}$ and control vSMCs (group III) was performed and overlap between gene lists derived from the two sex-specific comparison and the initial data were identified. **(B)** UMAP plot of scRNA-seq visualizing spread of data from vSMCs of the three sexes identified with N/A indicating those cells whose chromosomal sex could not be confirmed **(C)** Venn diagram visualizing the overlap between significantly downregulated genes identified in each of the sex-specific comparisons as well as the combined dataset. **(D)** Venn diagram visualizing the overlap between significantly upregulated genes identified in each of the sex-specific comparisons as well as the combined dataset. For **C** and **D**, below each intersection category is the list of genes belonging to the indicated intersection (at p adj ≤ 0.05). Genes in italicized, bold red font are those belonging to the top 50 differential expressed vSMC genes.

Supplemental Figure 6. vSMC deficiencies as a result of Notch3 loss affects arteries of multiple organs, an effect exacerbated by aging. (A) Volumetric analysis of middle cerebral arteries (MCA) from the Circle of Willis to 3000μm in Z axis (from the MCA branch point upward towards the posterior of the brain) in young (1 month) control and *Notch3*$^{-/-}$ mice. Maximum intensity projection of MCA vessels color-coded by volume and overlayed on the base micro-CT visualization (white) in the lateral view. **(B)** Quantification of MCA volumes at 1 month in a cohort of *Notch3*$^{-/-}$ and control littermates. Mean+/-SD, n= 6, student's t-test with Welch's correction. **(C)** Staining of arterial blood vessels, immunolabeled with alpha-SMA (to highlight vascular smooth muscle) from each of the indicated organs in 24+ month old male control and *Notch3*$^{-/-}$ animals. Yellow arrows indicate sites with loss of vascular smooth muscle coverage.

Supplemental Figure 7. Quality control and characterization of young (1 month) scRNA-seq datasets. (A) Violin plots visualizing the number of identified transcripts (nFeature_RNA) and distribution of average transcriptional expression (nCount_RNA) by genotype in the scRNA-seq data. **(B)** UMAP plot of scRNA-seq data visualizing spread of all cells by genotype. **(C)** Violin plots visualizing the number of identified transcripts and distribution of average transcriptional expression by cluster in the scRNA-seq data. **(D)** UMAP plot of scRNA-seq data visualizing spread of data by identified clusters. **(E)** Distribution of cells in each cluster by genotype as percentage. **(F)** Dot plot visualizing the expression of classical marker genes used to identify cell types in the young scRNA-seq data (EC - endothelial cell). **(G)** UMAP plot of scRNA-seq data visualizing spread of all cells by cell type. **(H)** Violin plots visualizing the expression of I) classical vascular smooth muscle markers, II) Notch Receptors, III) Notch Ligands and IV) Canonical Notch target genes in the vascular cell types (smooth muscle, pericyte, arterial, capillary and venous endothelium).

Supplemental Figure 8. Quality control and characterization of aged (24 month) scRNA-seq datasets. (A) Violin plots visualizing the number of identified transcripts (nFeature_RNA) and distribution of average transcriptional expression (nCount_RNA) by genotype in the scRNA-seq data. **(B)** UMAP plot of scRNA-seq visualizing spread of all cells by genotype. **(C)** Violin plots visualizing the number of identified transcripts and distribution of average transcriptional expression by cluster in the scRNA-seq data. **(D)** UMAP plot of scRNA-seq visualizing spread of all cell types by cluster. **(E)** Distribution of cells in each cluster by genotype as percentage. **(F)** Dot plot visualizing the expression of classical marker genes used to identify cell types in the old

scRNA-seq data (EC - endothelial cell). **(G)** UMAP plot of scRNA-seq visualizing spread of all cells by cell type. **(H)** Violin plots visualizing the expression of I) classical vascular smooth muscle markers, II) Notch Receptors, III) Notch Ligands and IV) Canonical Notch target genes in the vascular cell types (smooth muscle, pericyte, arterial and venous endothelium).

Supplemental Figure 9. Unique and shared gene signatures identified in *Notch3*$^{-/-}$ vSMCs. (A) Graphical representation of the experimental design used to identify genes regulated by Notch3 in vascular smooth muscle cells at 1 month (Young), 12 month (Mature) and 24 month (Aged). **(B)** Upset plot visualizing overlap between three timepoint comparisons. Below each intersection category is the list of the genes belonging to this category (at padj< 0.05).

Supplemental Figure 10. Validation of *Notch3*$^{-/-}$ regulated genes in cross-sections of the middle cerebral artery (MCA). (A) Immunofluorescence (IF) staining of phosphorylated myosin light chain 2 (pMLC2) in red in the MCA sections from 18 month old male control and *Notch3*$^{-/-}$ mice. The arrowhead indicates absence of pMLC2 in alpha-smooth muscle actin (αSMA) positive cells. Violin plot of *Mylk* expression in young control and *Notch3*$^{-/-}$ vascular smooth muscle cells (vSMCs). **(B)** IF of Phospholamban (*Pln*) in red. The arrowhead indicates absence of co-localized staining between Pln and αSMA. Violin plot of Pln expression in young control and *Notch3*$^{-/-}$ vSMCs. **(C)** IF of collagen IV in red. The arrow indicate the increased deposition of collagen IV in the MCA of *Notch3*$^{-/-}$. Violin plot of *Col4a1* expression in young control and *Notch3*$^{-/-}$ vSMCs. **(D)** IF of Fibronectin (*Fn1*) in red. The arrow indicate the increased deposition of fibronectin in the MCA of *Notch3*$^{-/-}$. Violin plot of Fn1 expression in young control and *Notch3*$^{-/-}$ vSMCs. **(E)** IF of Sulfatase 1 (*Sulf1*) in red. The arrow indicates increased expression of SULF1 in MCA vSMCs from *Notch3*$^{-/-}$ mice. Violin plot of *Sulf1* expression in young control and *Notch3*$^{-/-}$ vSMCs. **(F)** IF of TMSB4X in red. The arrow indicates an increased expression of TMSB4X in MCA vSMCs from *Notch3*$^{-/-}$ mice. Violin plot of *Tmsb4x* in young control and *Notch3*$^{-/-}$ vSMCs. **(G)** IF of CCN3 (*Nov*) in red. The arrow indicates increased CCN3 expression in *Notch3*$^{-/-}$ vSMCs. Violin plot of *Nov* (CCN3) in young control and *Notch3*$^{-/-}$ vSMCs. **(H)** IF of SOD3 in red. The arrow indicates increased expression of SOD3 in *Notch3*$^{-/-}$ MCA vSMCs. Violin plot of *Sod3* expression in young control and *Notch3*$^{-/-}$ vSMCs.

Supplemental Figure 11. Drifts in the transcriptional profile of vascular cells in the absence of Notch3. (A) Heatmap visualizing top 40 differentially expressed genes in young (1 month) pericytes. Red arrow indicates the location of *Notch3*$^{-/-}$ in relation to other transcripts. **(B)** Heatmap visualizing top 40 differentially expressed genes in aged (24 month) pericytes. Red arrow indicates the location of *Notch3*$^{-/-}$ in relation to other transcripts. **(C)** Gene ontology enrichment of *Notch3*$^{-/-}$ pericytes differentially expressed gene signature including the top 3 unique ontology categories and selected transcripts within each category. Dot color indicates average enrichment (Log Fold Change), while size indicates significance of enrichment. **(D)** Heatmap visualizing top 40 differentially expressed genes in young arterial endothelial cells. Pink ovals identify genes critical in vascular development. **(E)** Heatmap visualizing top 40 differentially expressed in old arterial endothelial cells. Pink ovals identify genes critical in vascular development. **(F)** Gene Ontology enrichment of *Notch3*$^{-/-}$ arterial endothelial cell gene signature including the top 3 unique ontology categories and selected transcripts within each category.

Supplemental Figure 12. *Notch3* deficiency results in reduced levels of myosin light chain phosphorylation at 1 month. (A) vSMC enriched lysates from mixed sex cohorts of *Notch3*$^{-/-}$ and control aortas at one month of age were evaluated for the expression of pMLC2, MLC2, calponin, and lamin A (as a loading control).

Supplemental Figure 13. Significant alterations in the association of penetrating arteries with astrocytes and metabolic changes in cortical neurons upon Notch3 deficiency. (A) PECAM immunofluorescence (IF) of coronal brain sections to highlight the cerebral vasculature in 24-month-old male control and *Notch3*$^{-/-}$ animals. Red arrows highlight the presence of prominent penetrating arteries in the control animals. Increases in the diameter of penetrating arteries affects their identification in *Notch3*$^{-/-}$ mice. **(B)** Quantification of the inner diameter of penetrating arteries in control littermates and *Notch3*$^{-/-}$ mice at 24months. **(C)** PECAM (white) and GFAP (red) IF of penetrating arteries in 24-month-old male control and *Notch3*$^{-/-}$ animals. White dotted lines indicate locations of gaps in GFAP+ coverage of the penetrating arteries. **(D)** Quantification of vessel covered by GFAP+ astrocytes in 24-month control and *Notch3*$^{-/-}$. For **B** and **D**, error bars represent mean± SD, n = 5 mice per group. Welch's test. **(E)** Transversal sections of penetrating arteries stained with PECAM (white) and GFAP (red) IF to assess alterations in the perivascular space. **(F)** Quantification of the perivascular space in penetrating arteries from 12 month control and *Notch3*$^{-/-}$. Error bars represent mean± SD, n = 3 mice per group. Welch's test. **(I)** UMAP plot of scRNA-seq data in young (1 month) cortical neurons of the indicated genotypes. **(J)** Heatmap of the top 30 differentially expressed genes in young *Notch3*$^{-/-}$ cortical neurons relative to control. **(K)** Network visualization of KEGG pathway enrichment and selected member genes in young cortical neurons.

Supplemental Figure 1

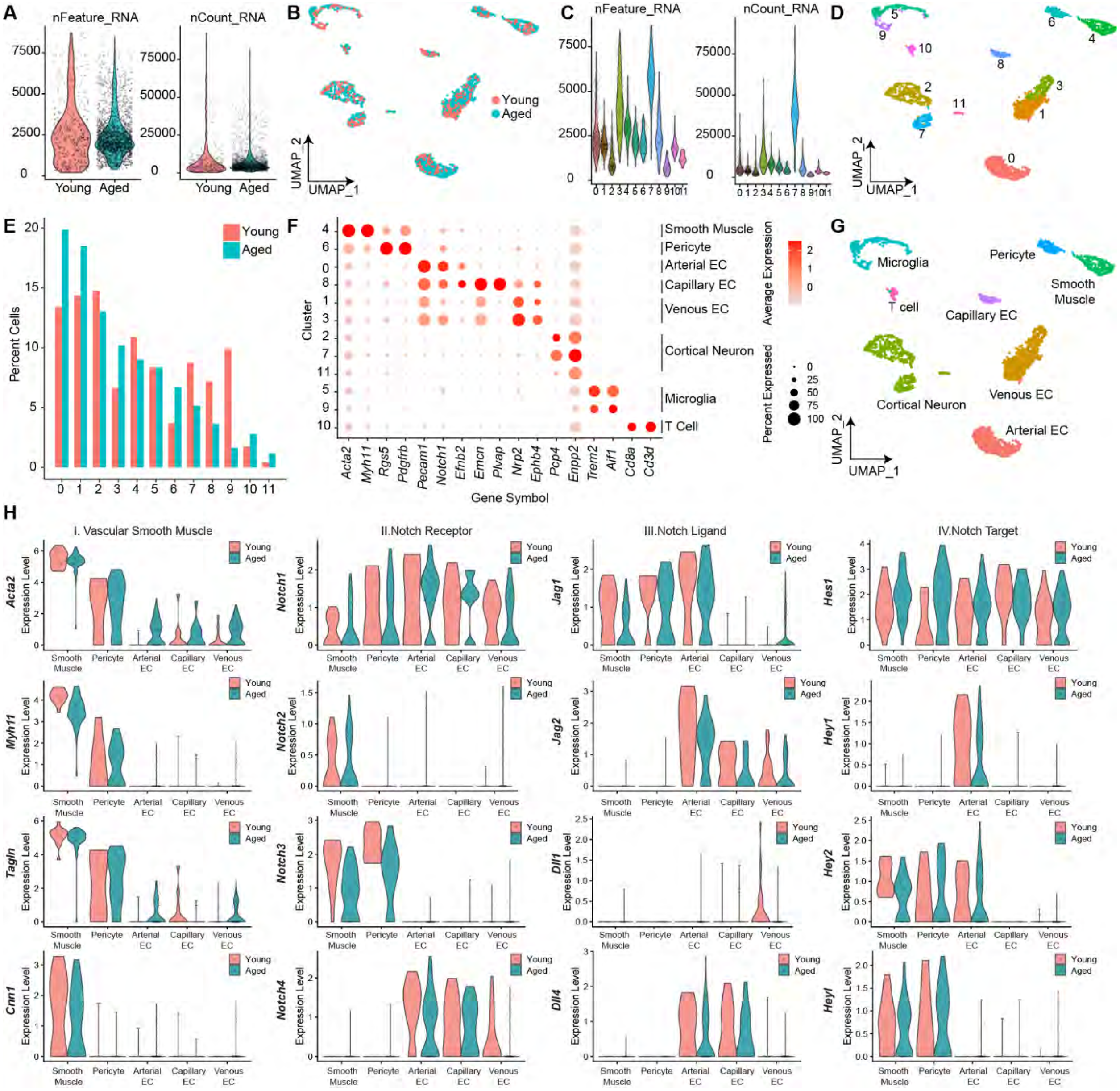

Supplemental Figure 2

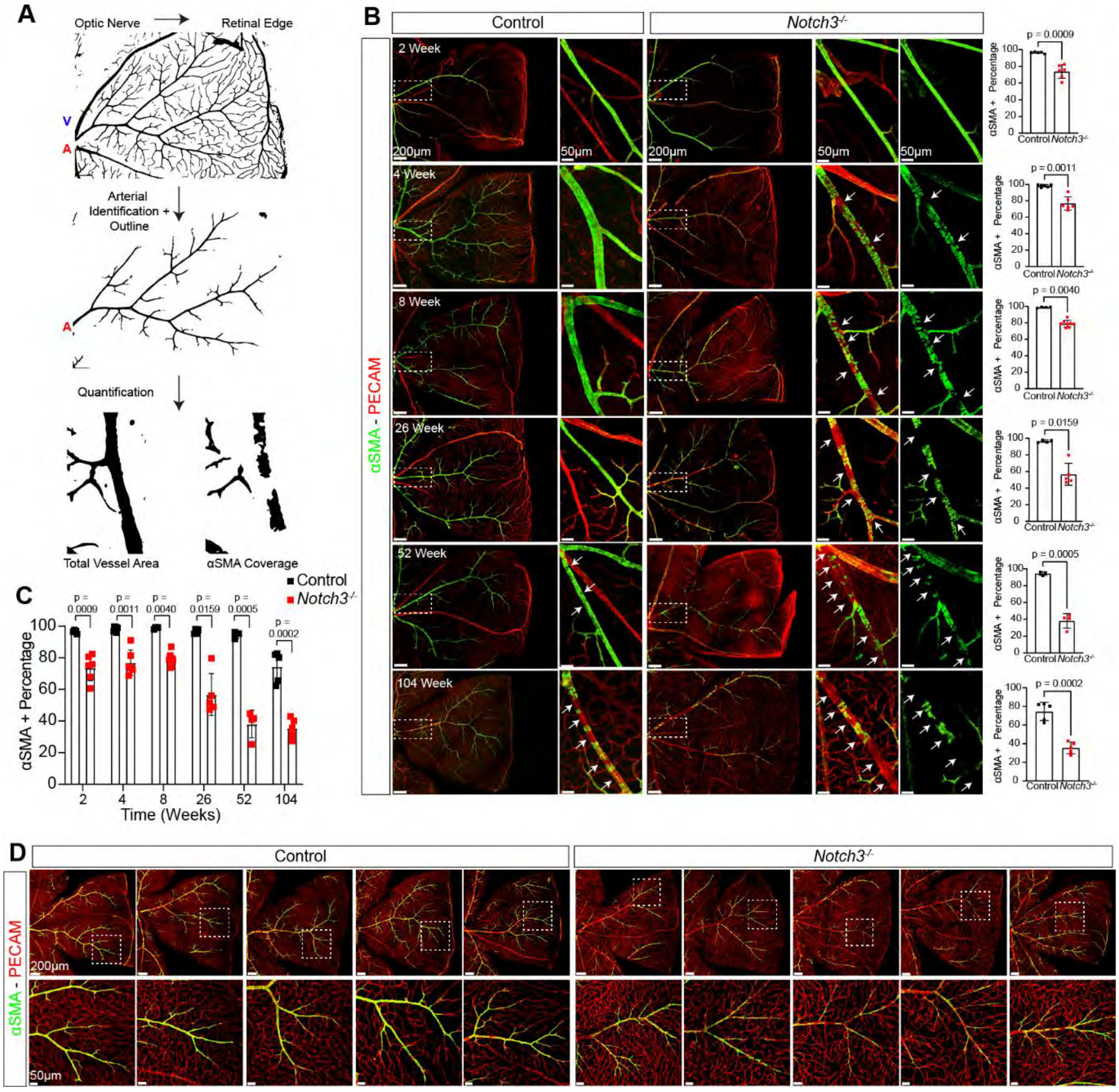

Supplemental Figure 3

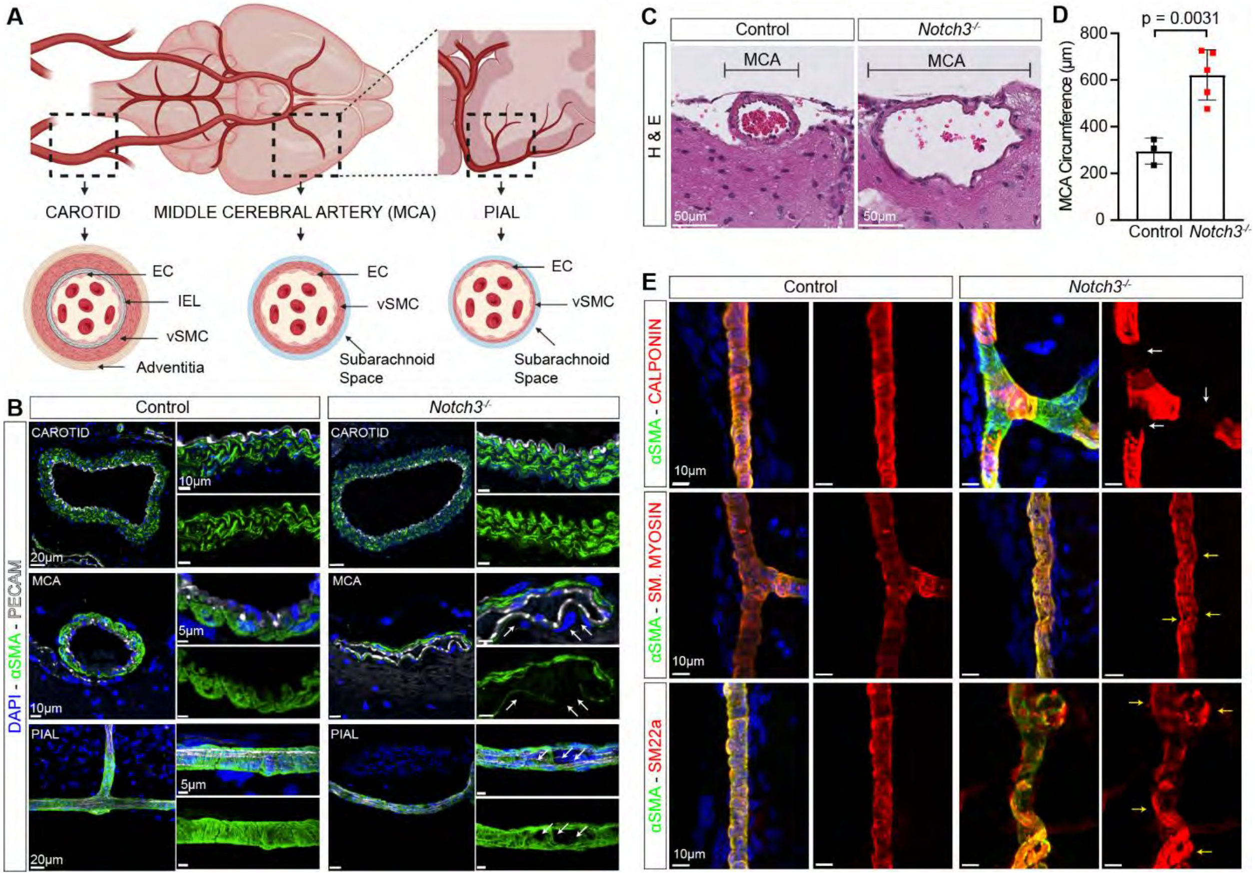

Supplemental Figure 4

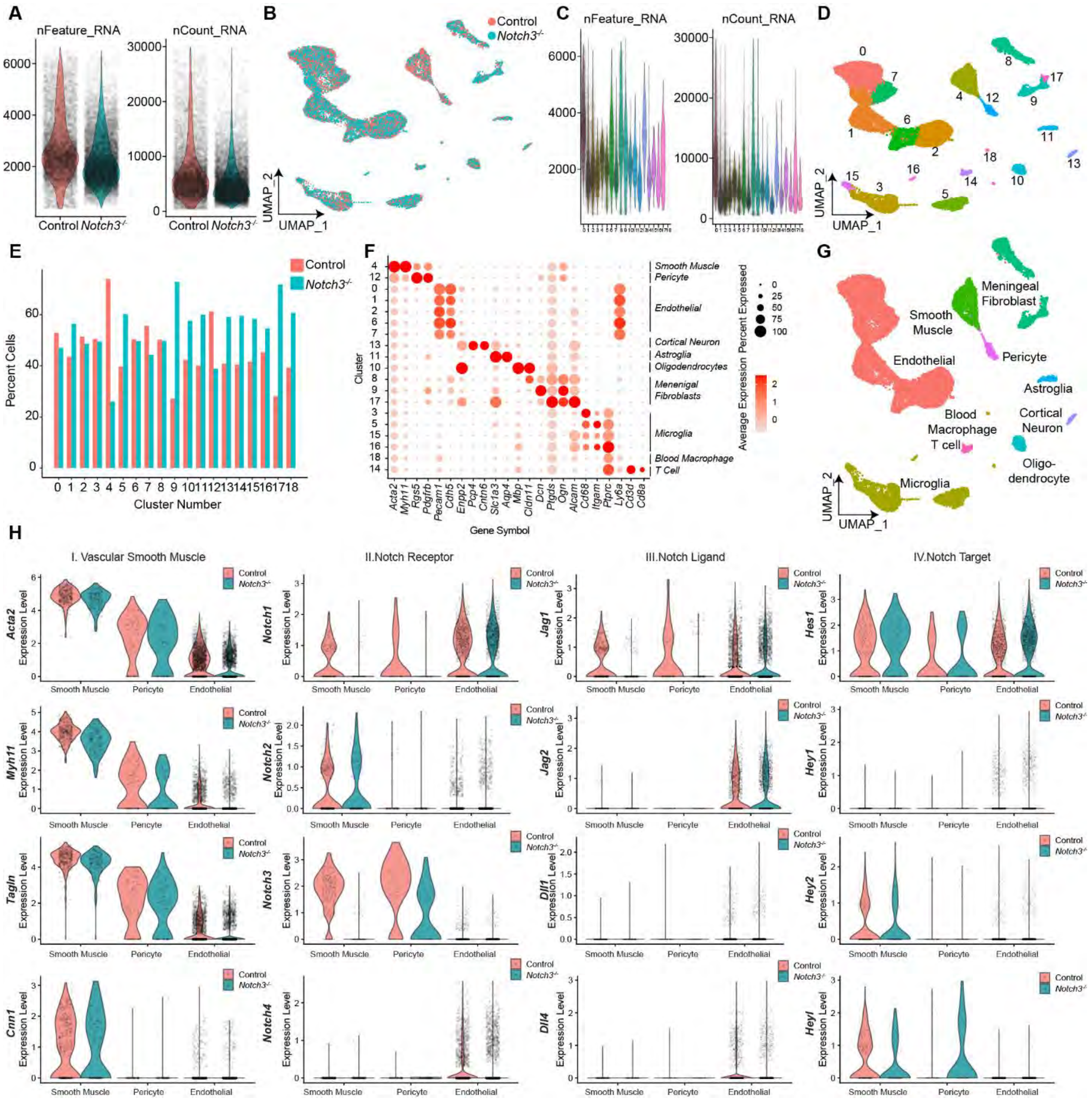

Supplemental Figure 5

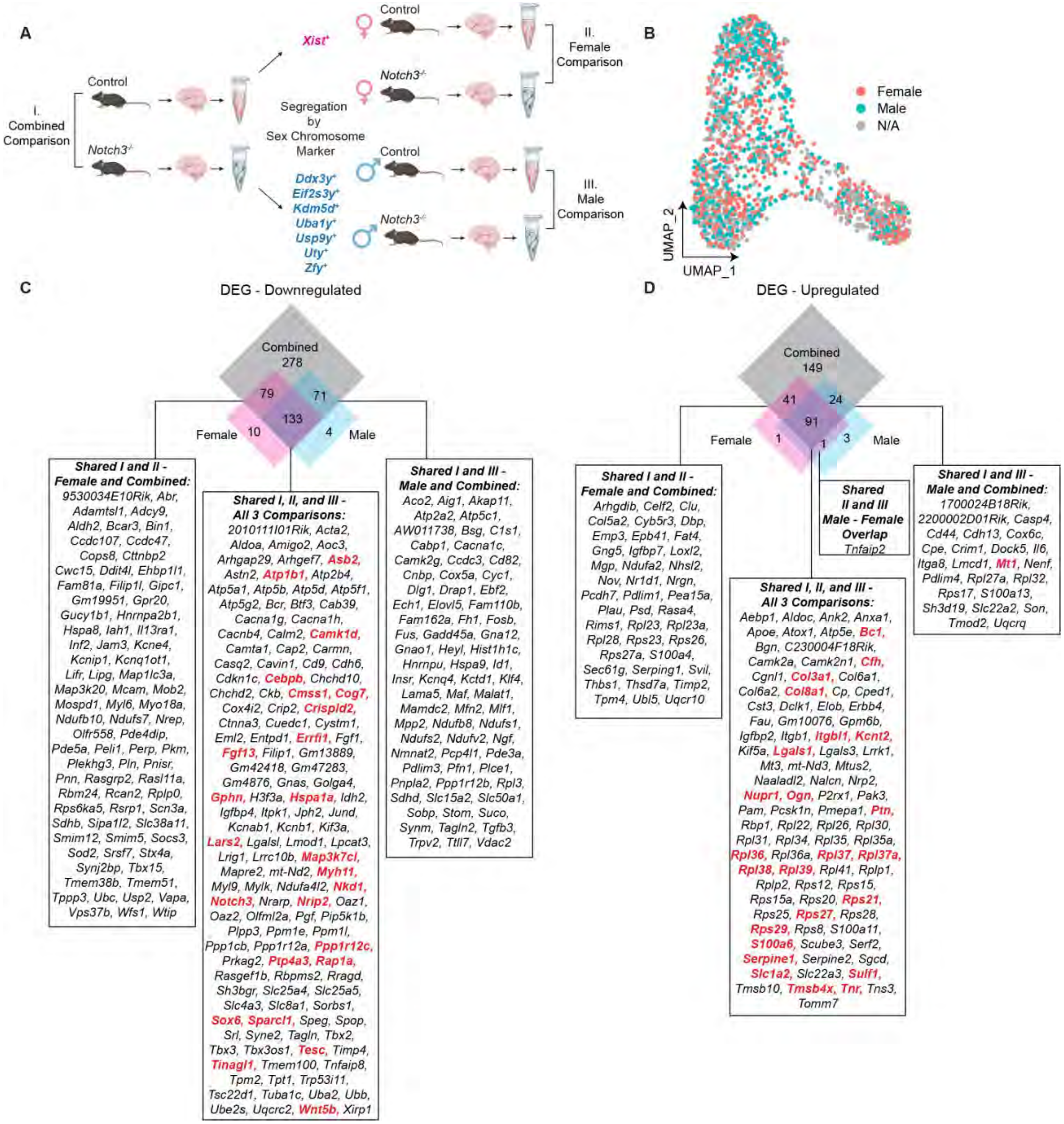

Supplemental Figure 6

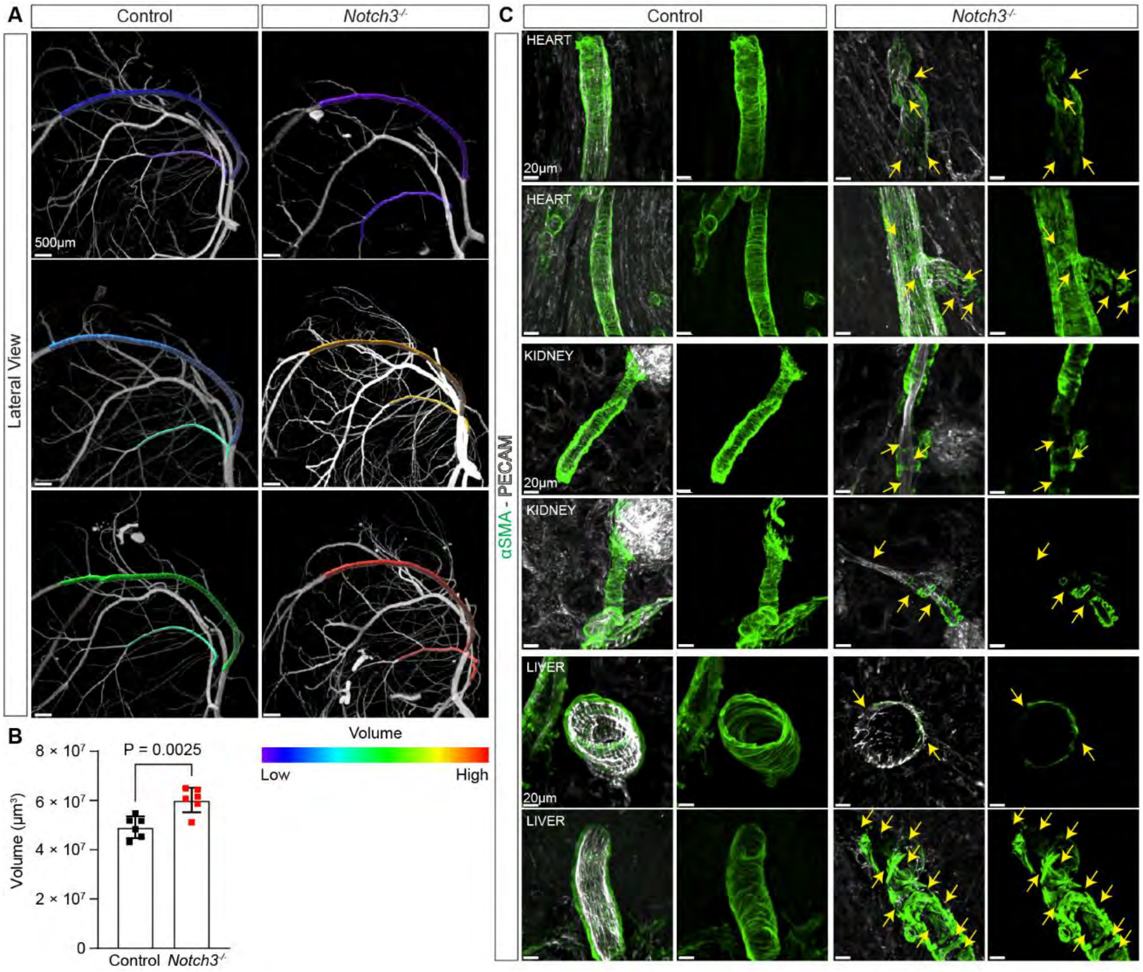

Supplemental Figure 7

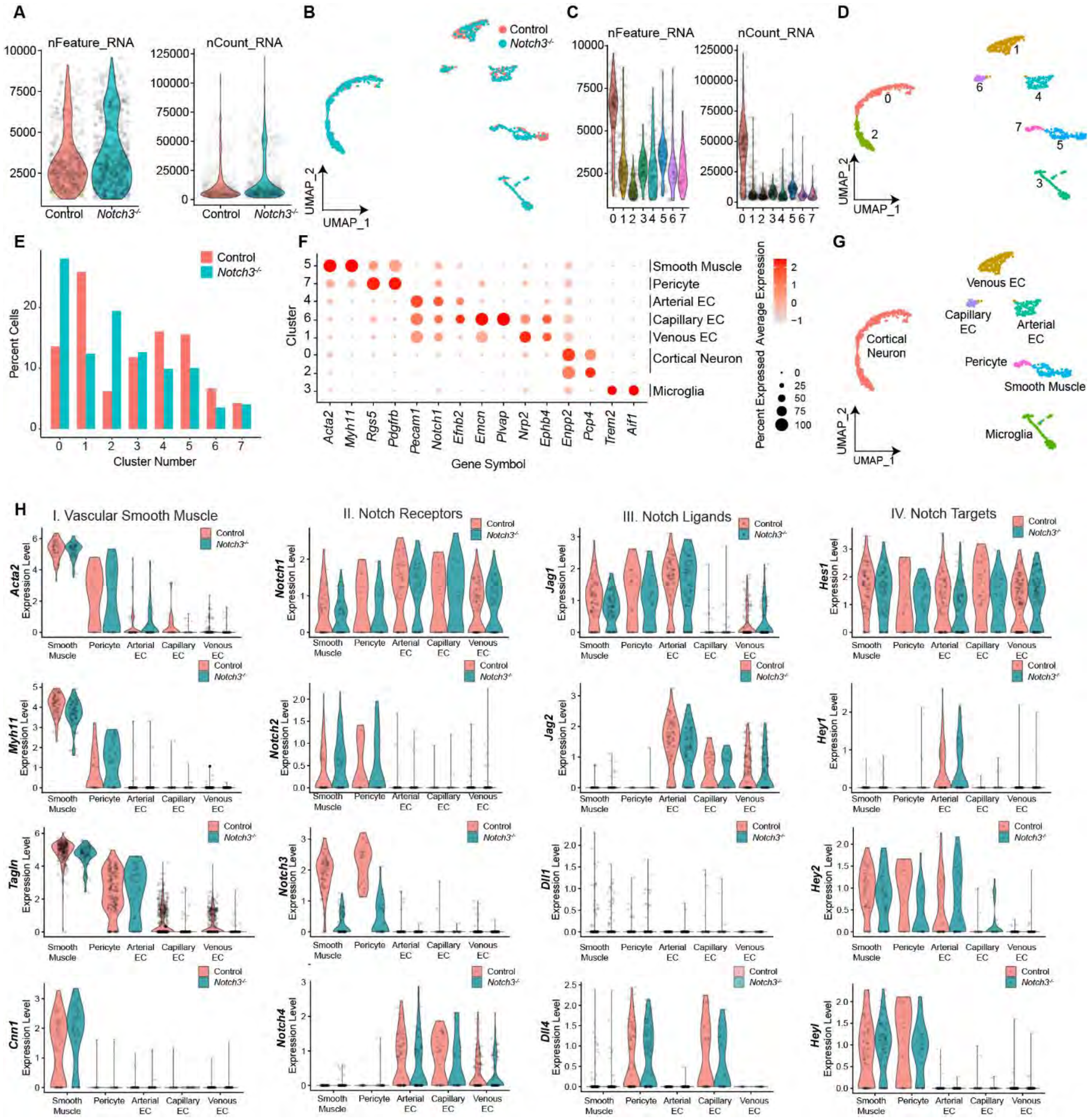

Supplemental Figure 8

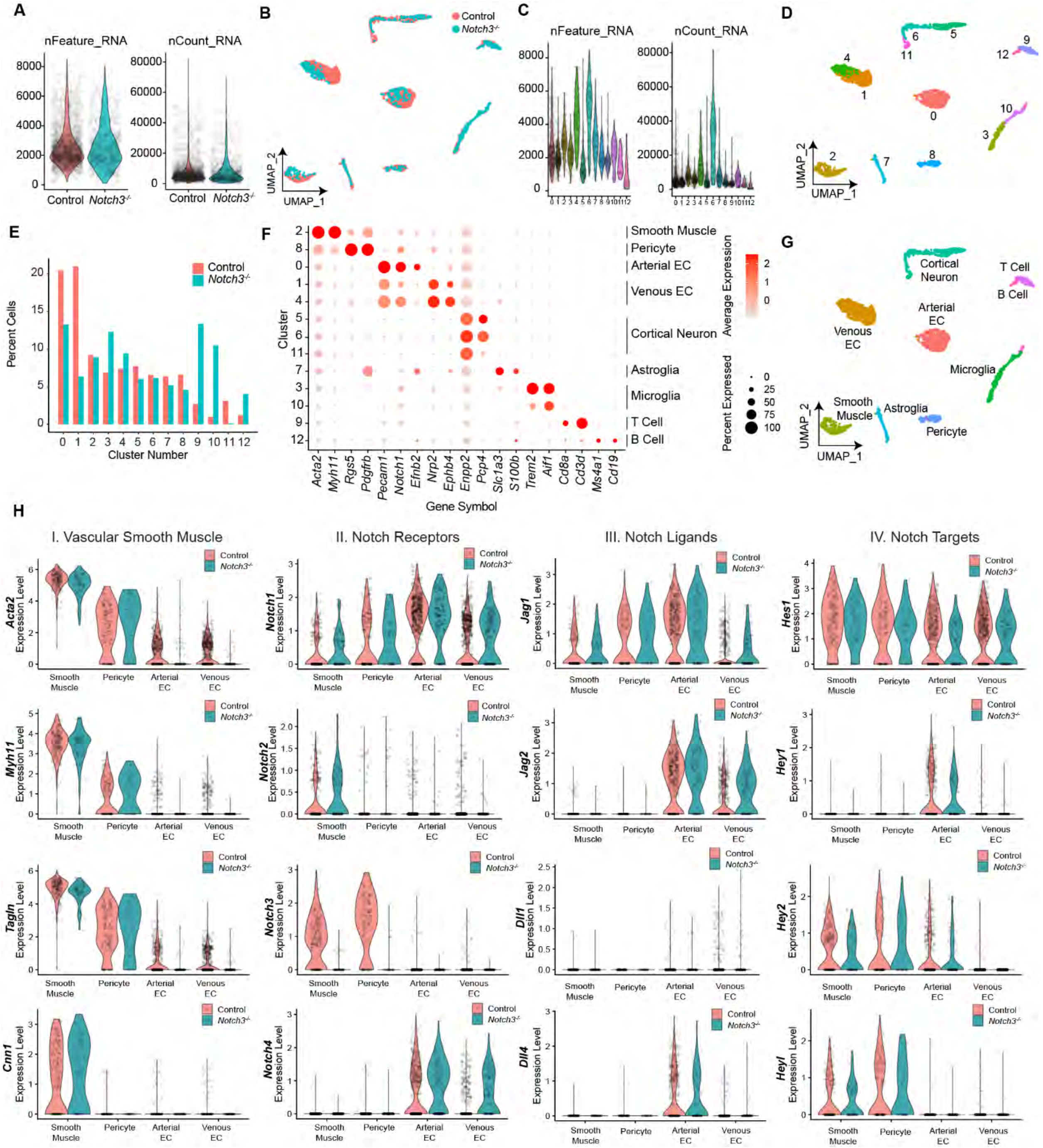

Supplemental Figure 9

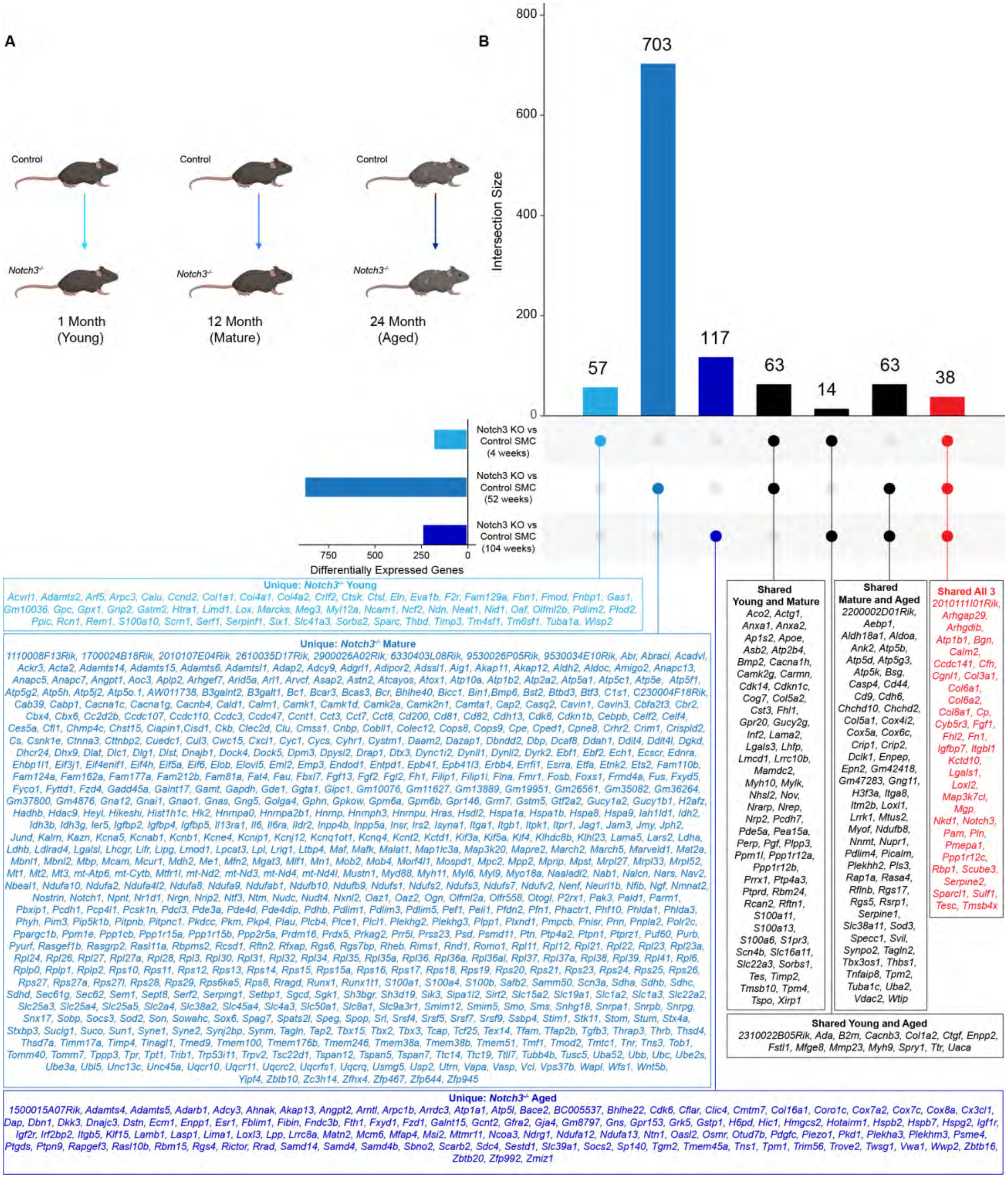

Supplemental Figure 10

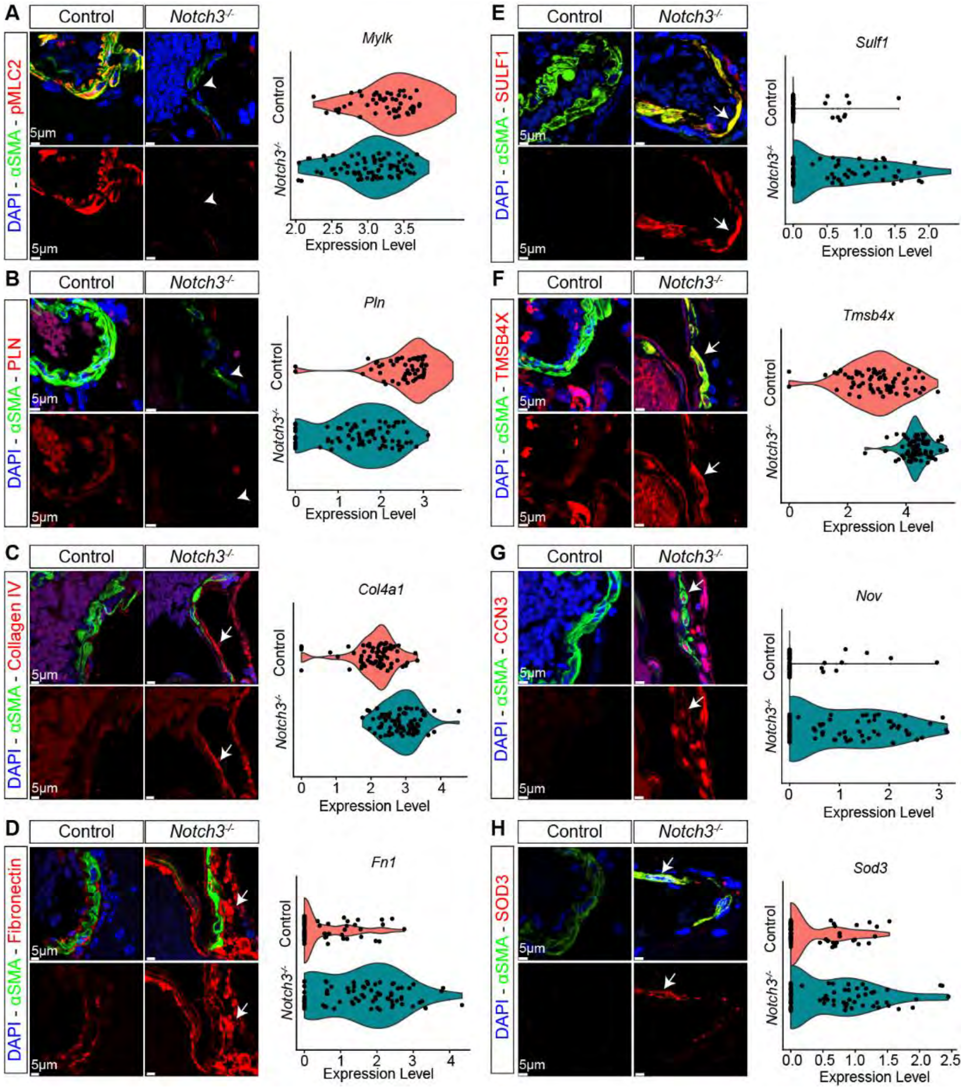

Supplemental Figure 11

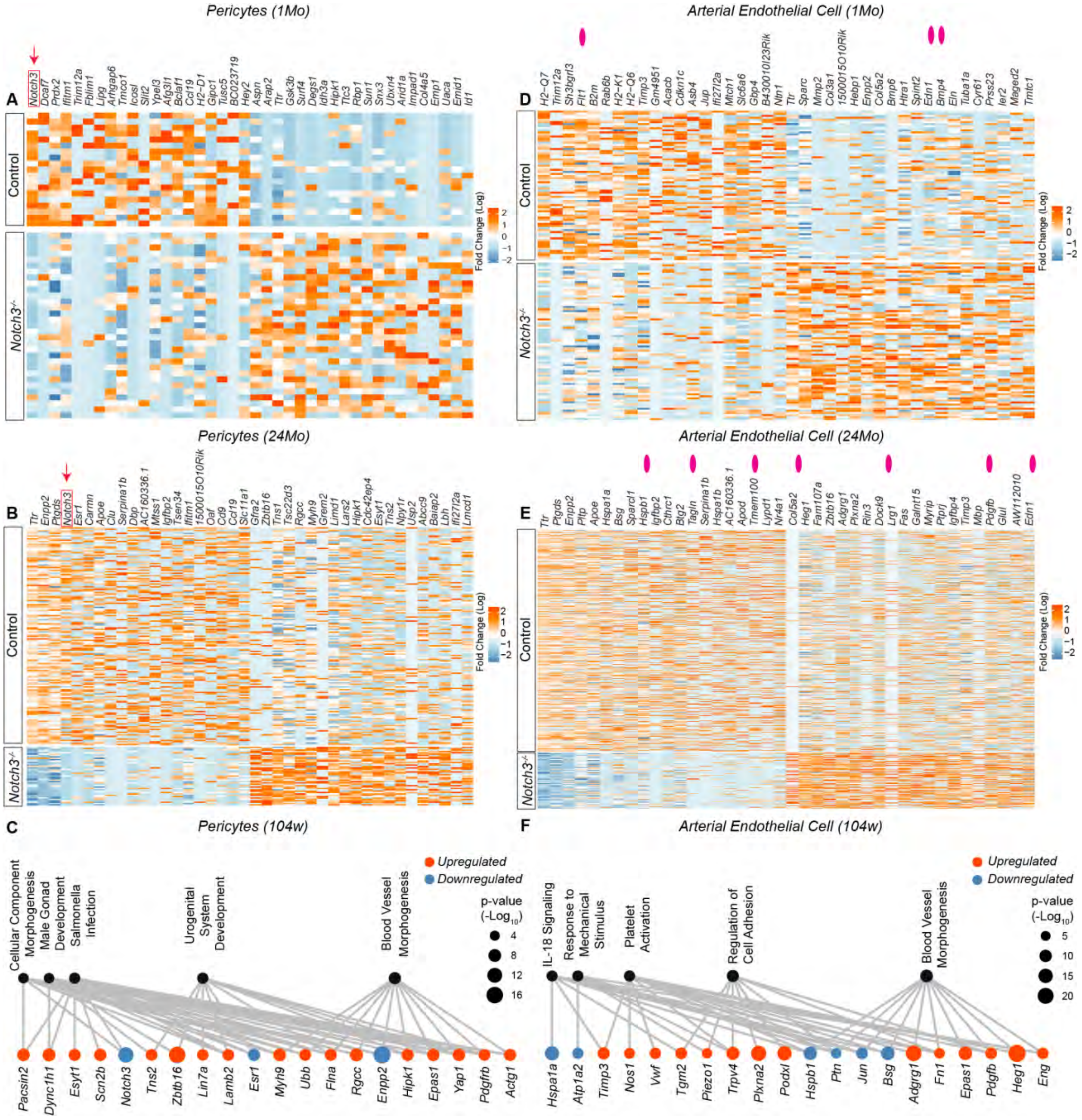

Supplemental Figure 12

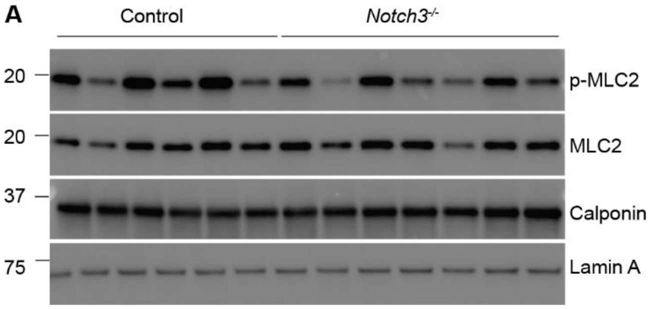

Supplemental Figure 13

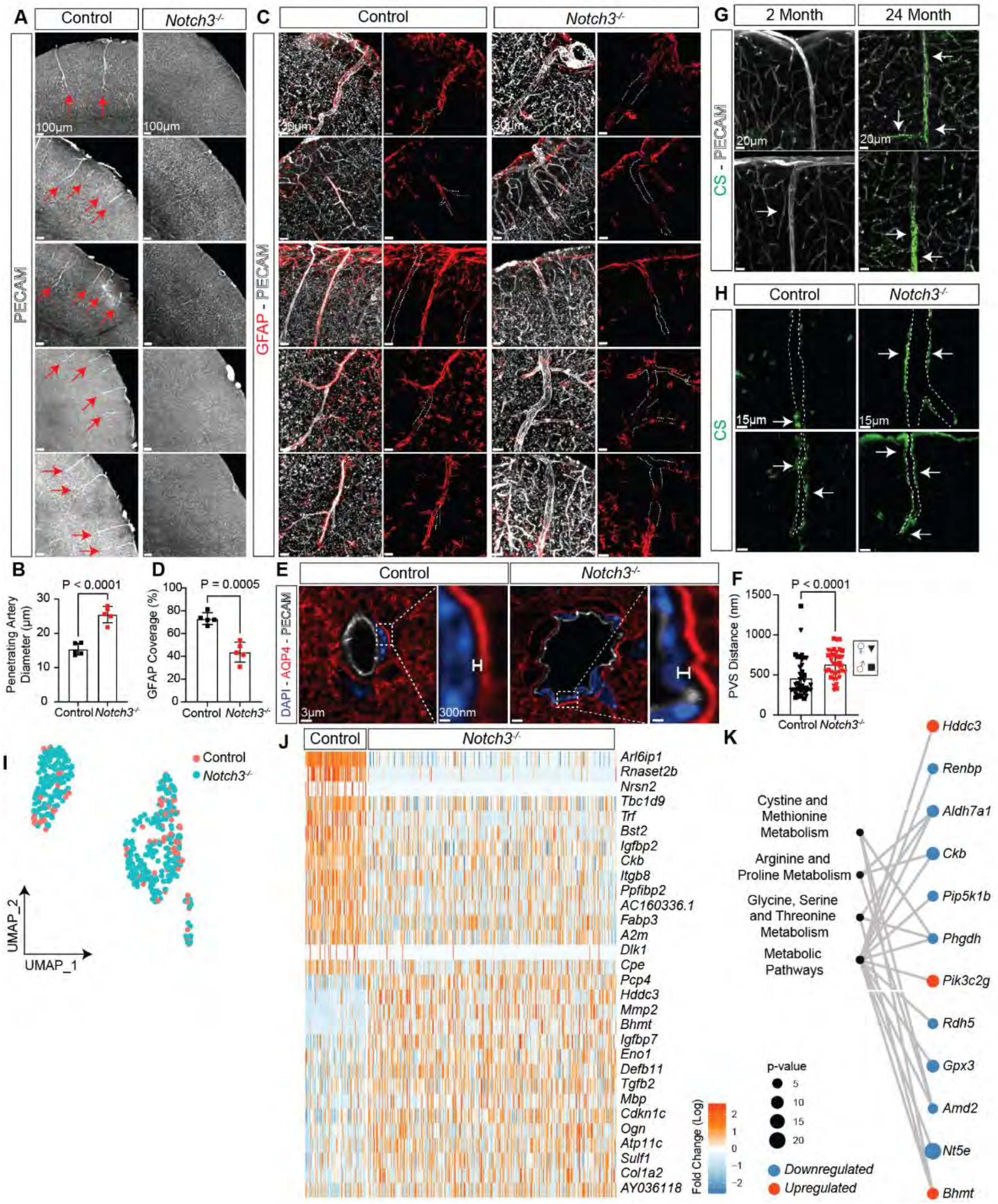

Supplement: Supplemental data [file jci-134-166134-s235.pdf]
