# Supplementary material for: Age-related loss of Notch3 underlies brain vascular contractility deficiencies, glymphatic dysfunction, and neurodegeneration in mice
Source: J Clin Invest. 2024 Jan 16;134(2):e166134. doi: 10.1172/JCI166134 (PMC10786701; doi:10.1172/JCI166134)

Full unedited gel for Figure 4E – 1 Month Samples

Phospho-Myosin Light Chain 2 (Ser19) Antibody #3671 Cell Signaling Technology

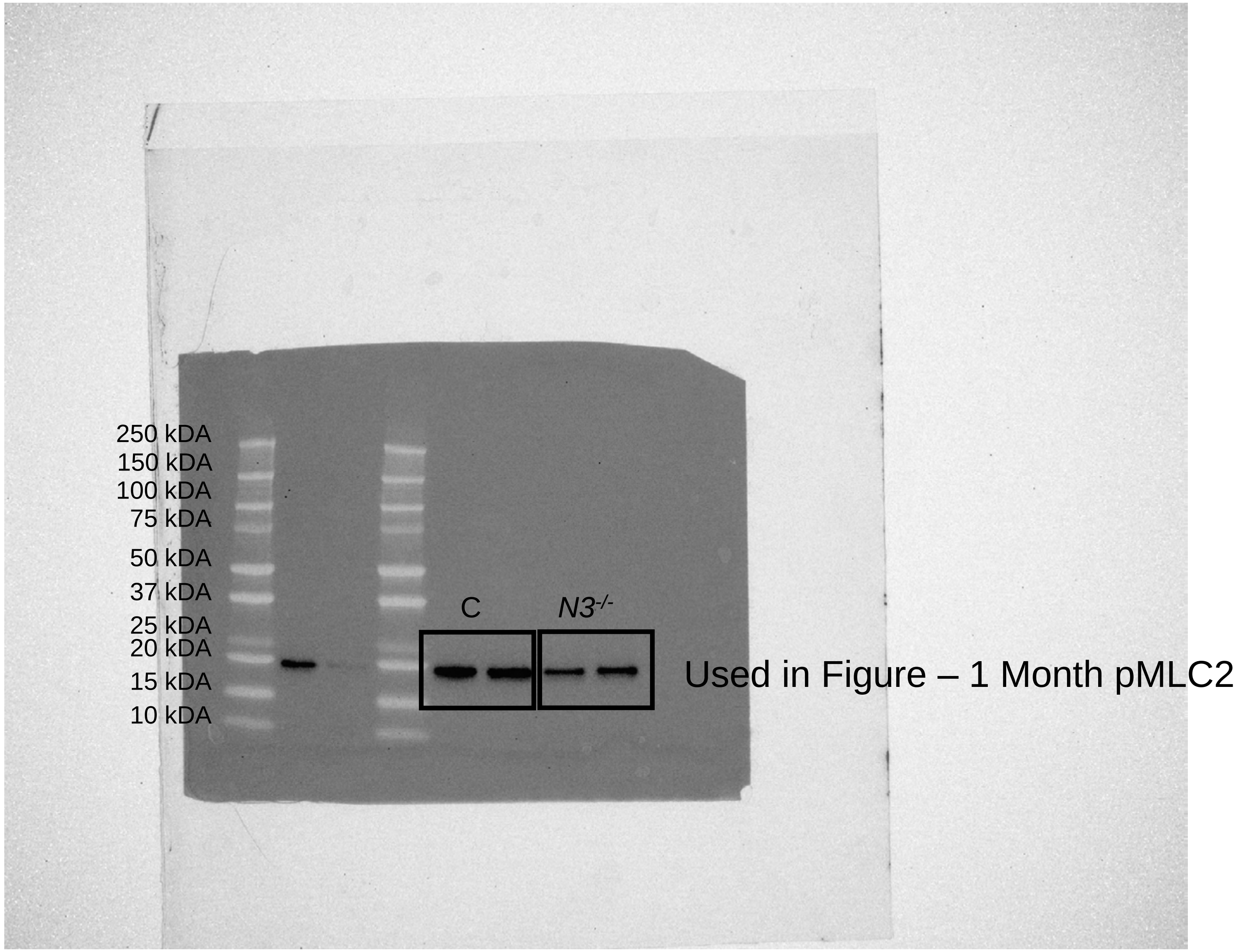

Full unedited gel for Figure 4E – 1 Month Samples

Myosin Light Chain 2 (D18E2) #8505 Cell Signaling Technology

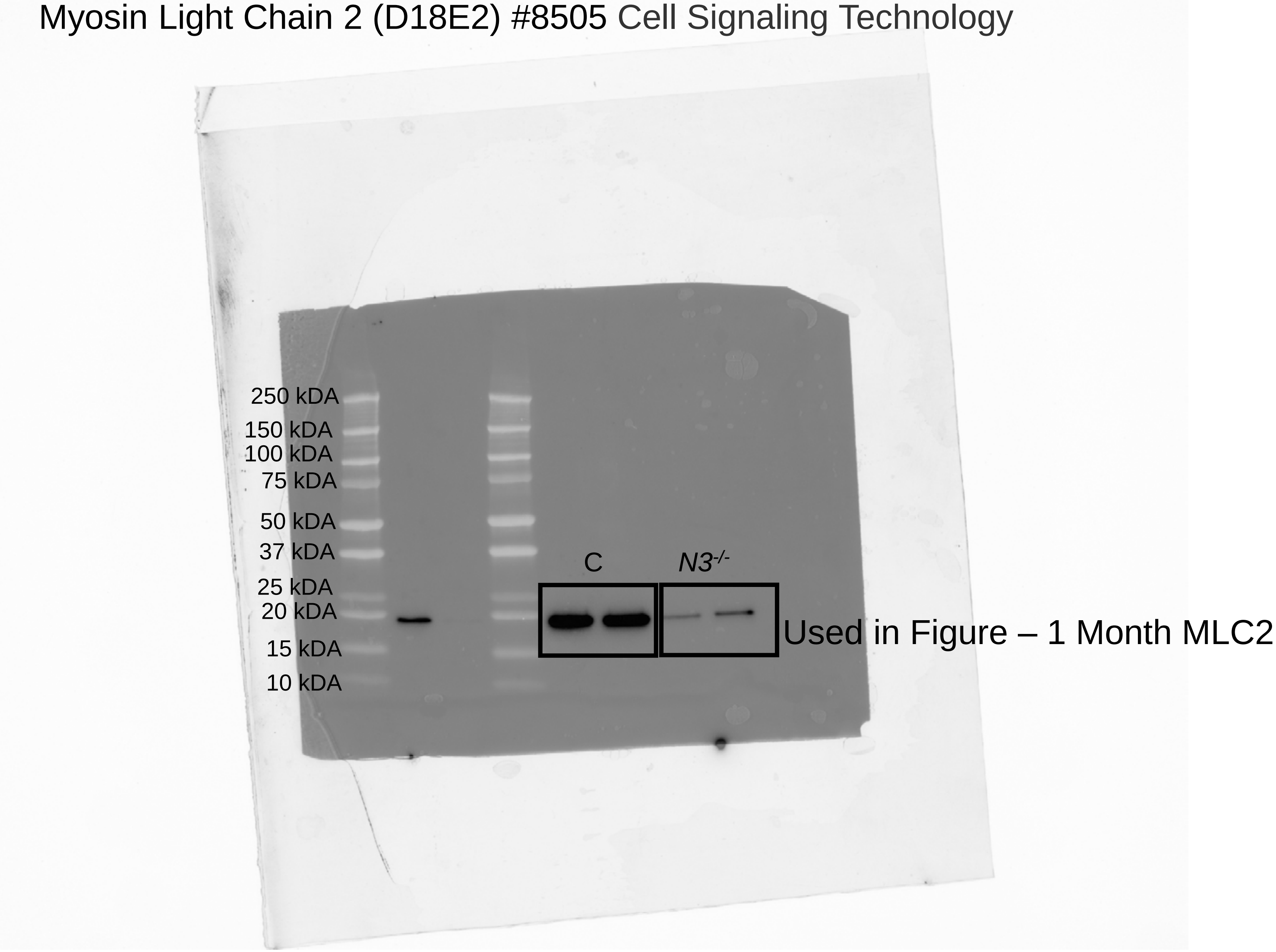

Full unedited gel for Figure 4E – 1 Month Samples

Calponin1 ab46794 - Abcam

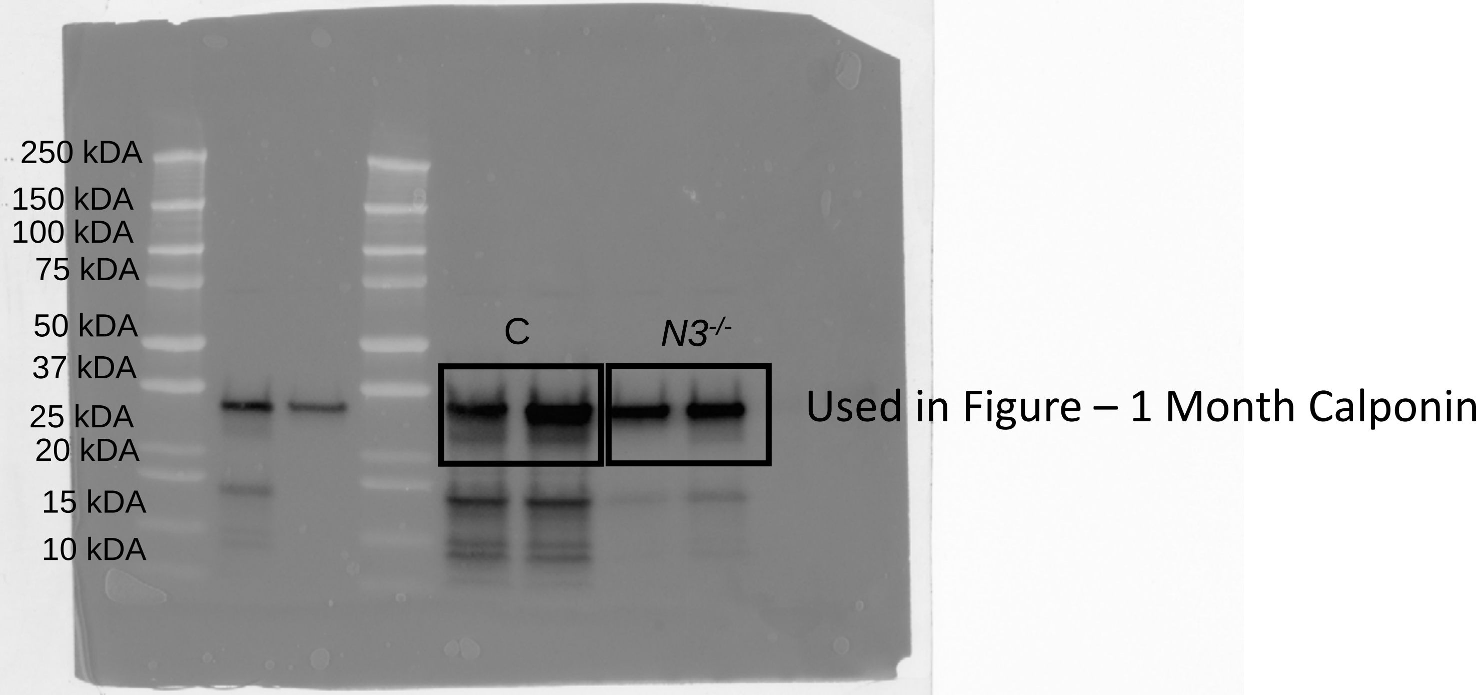

Full unedited gel for Figure 4E – 1 Month Samples

Lamin A ab26300 - Abcam

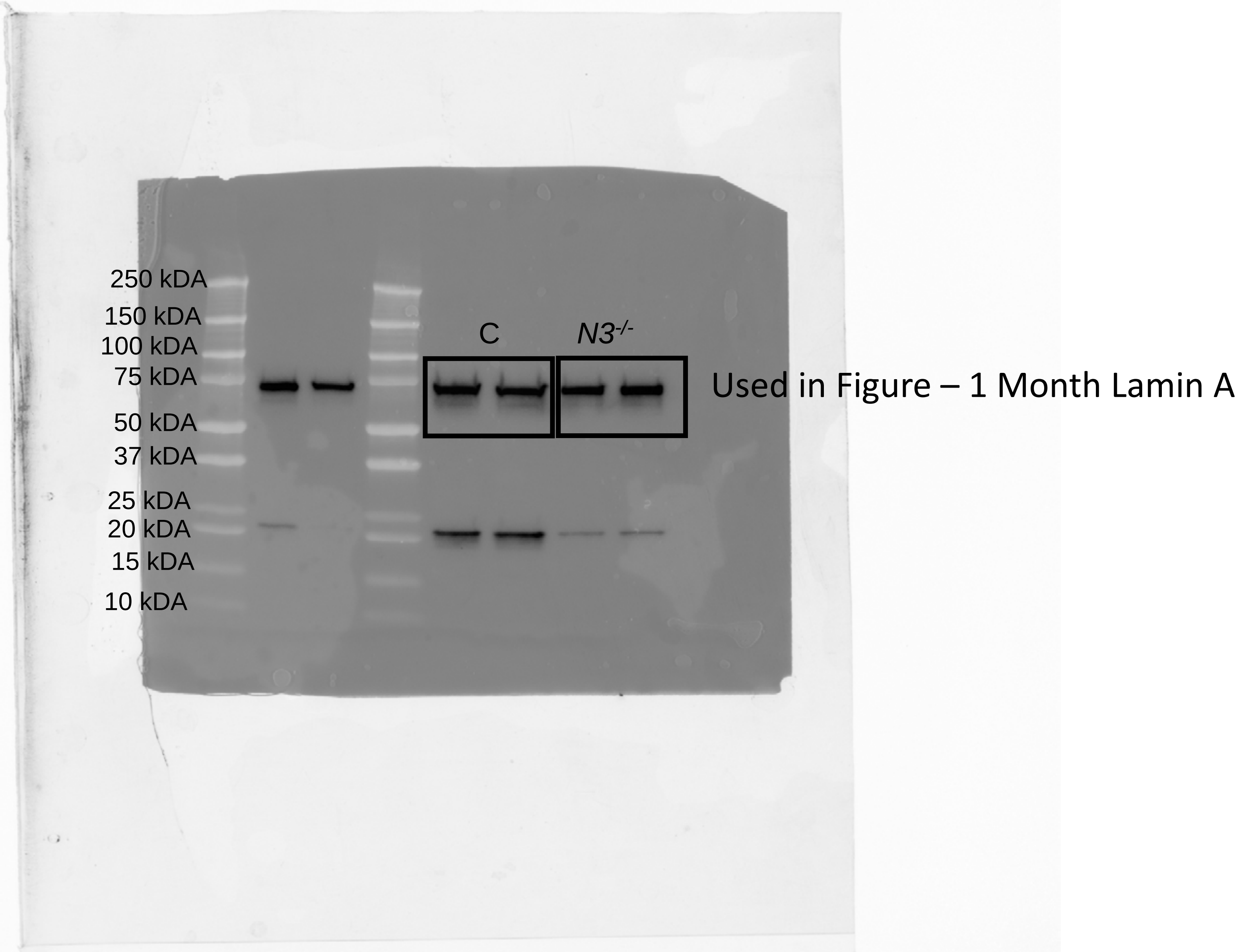

Full unedited gel for Figure 4E – 6 + 12 Month Samples

Phospho-Myosin Light Chain 2 (Ser19) Antibody #3671 Cell Signaling Technology

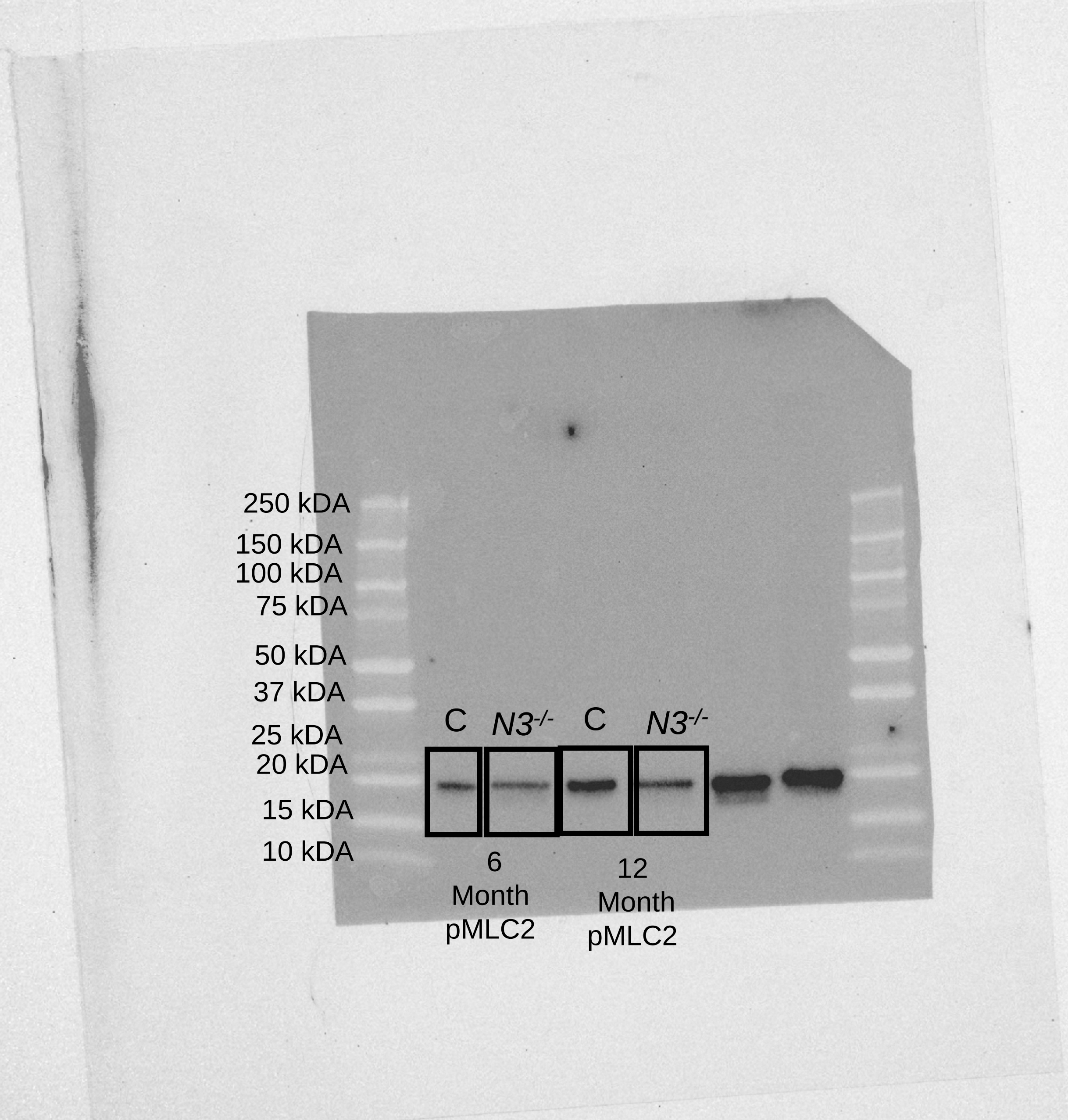

Full unedited gel for Figure 4E – 6 + 12 Month Samples

Myosin Light Chain 2 (D18E2) #8505 Cell Signaling Technology

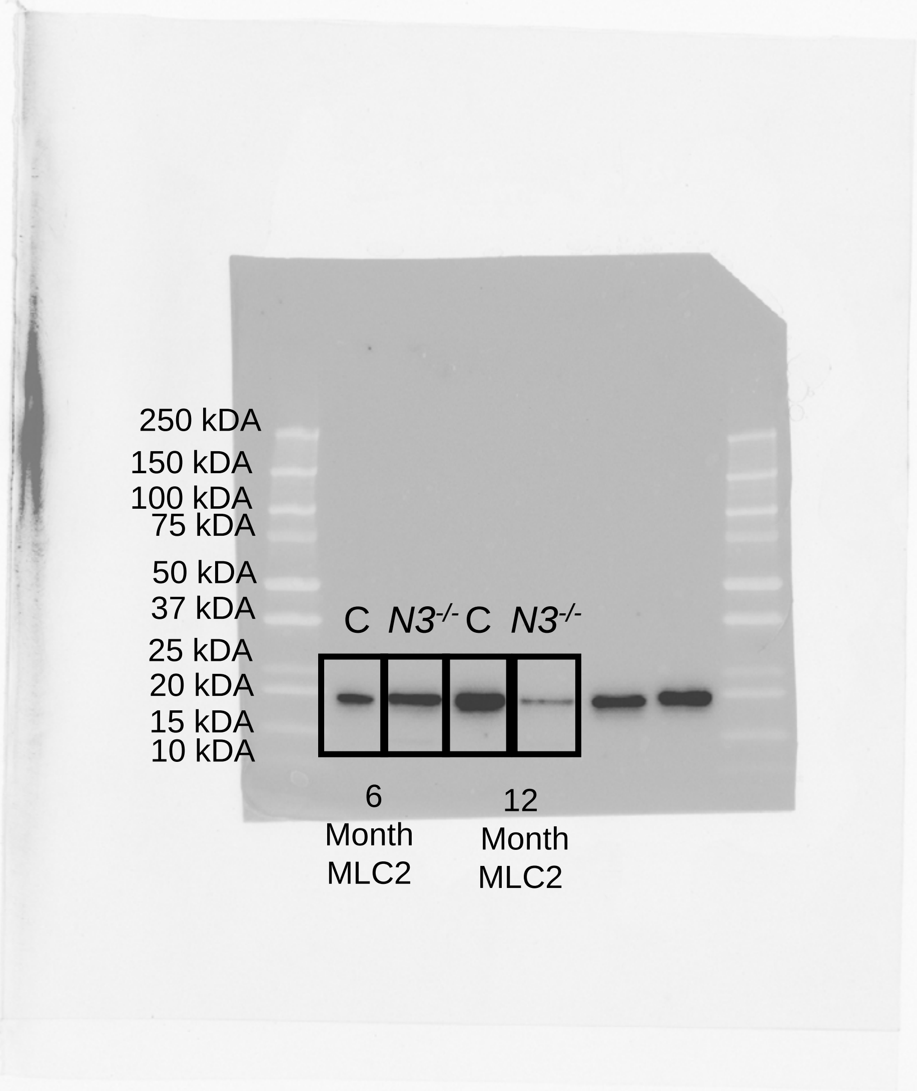

Full unedited gel for Figure 4E – 6 + 12 Month Samples

Calponin1 ab46794 - Abcam

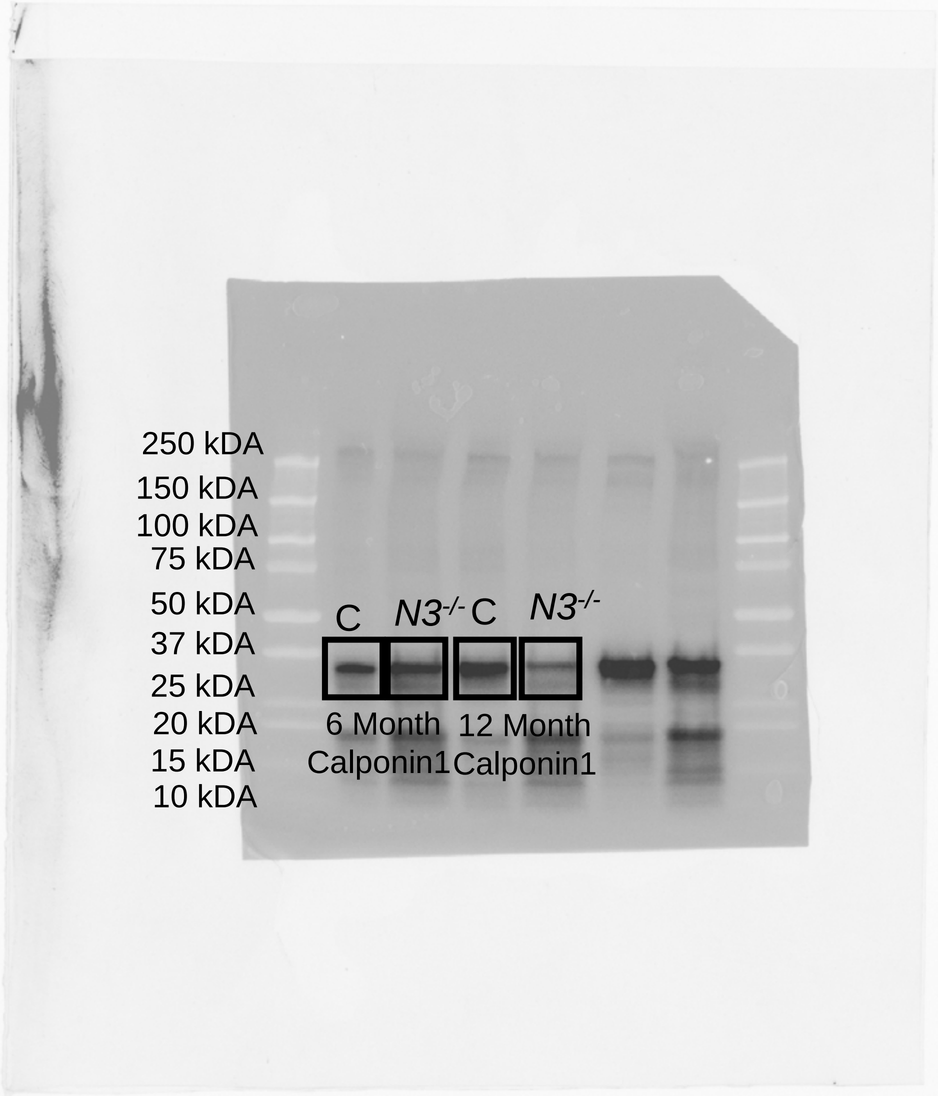

Full unedited gel for Figure 4E – 6 + 12 Month Samples

Lamin A ab26300 - Abcam

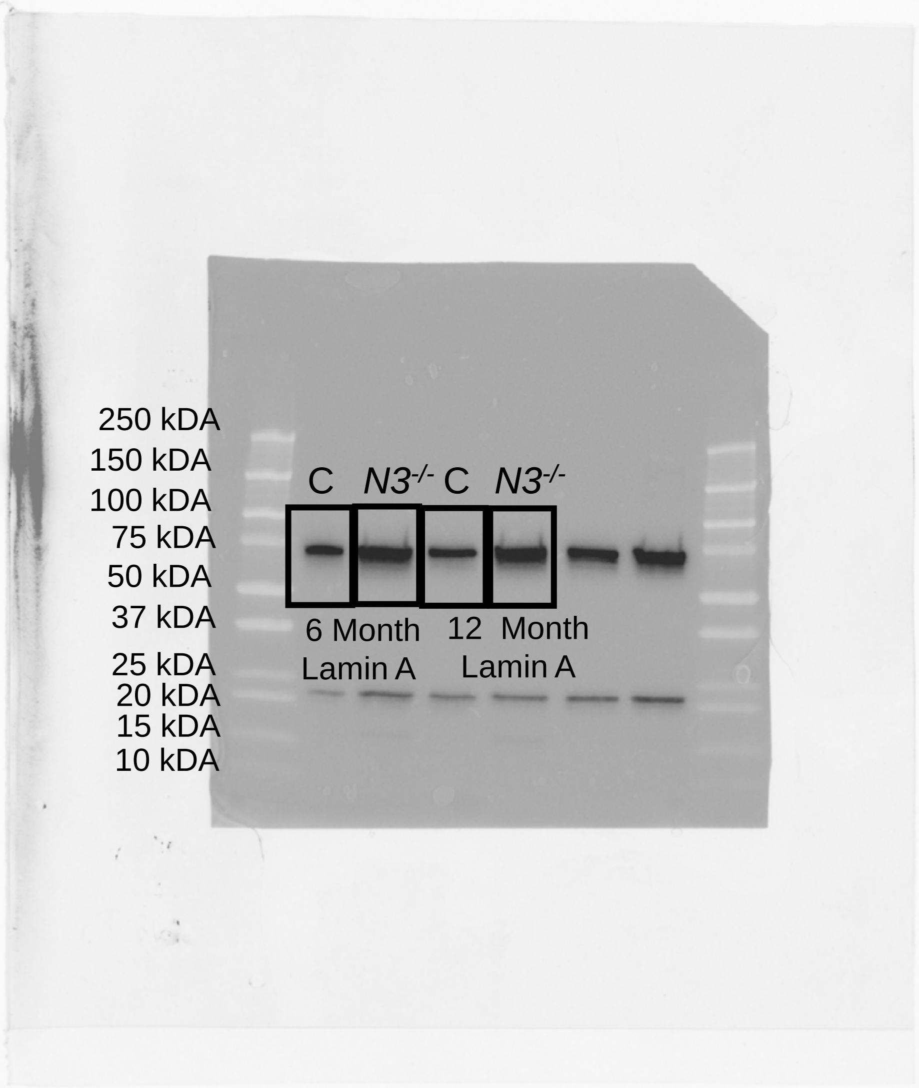

Full unedited gel for Supplemental Figure 11 – 1 Month Samples

Phospho-Myosin Light Chain 2 (Ser19) Antibody #3671 Cell Signaling Technology

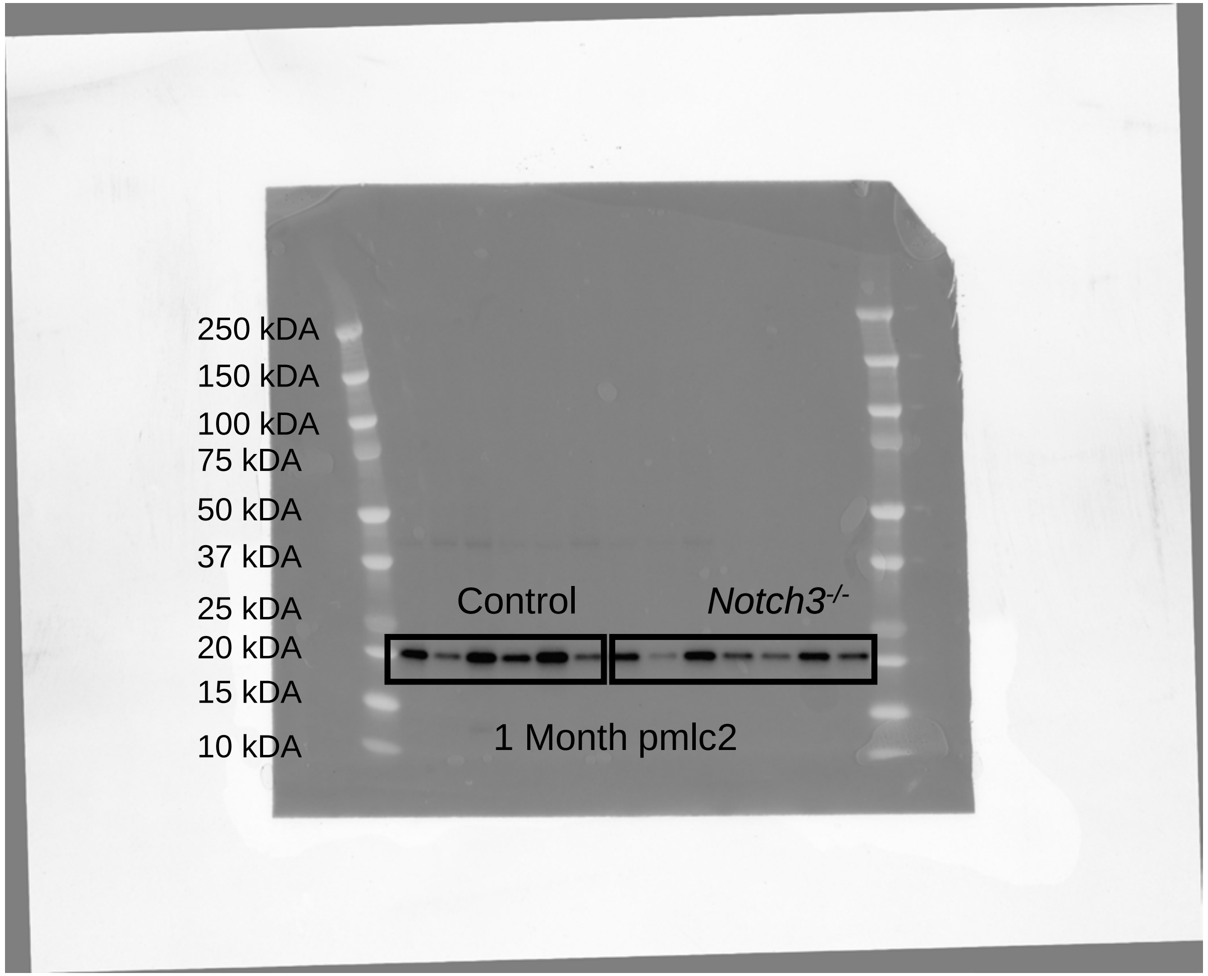

Full unedited gel for Supplemental Figure 11 – 1 Month Samples

Myosin Light Chain 2 (D18E2) #8505 Cell Signaling Technology

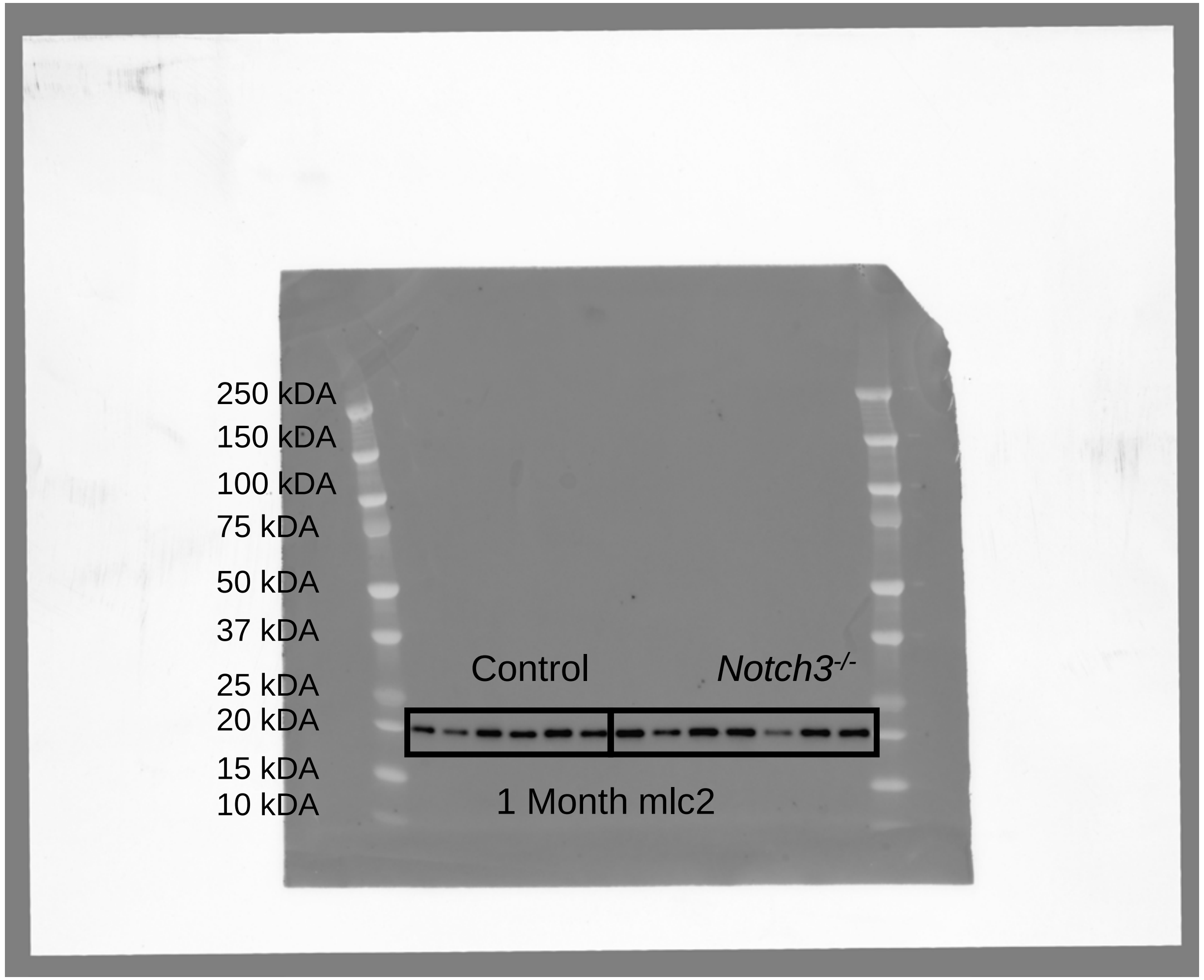

Full unedited gel for Supplemental Figure 11 – 1 Month Samples
Calponin1 ab46794 – Abcam
Lamin A ab26300 - Abcam

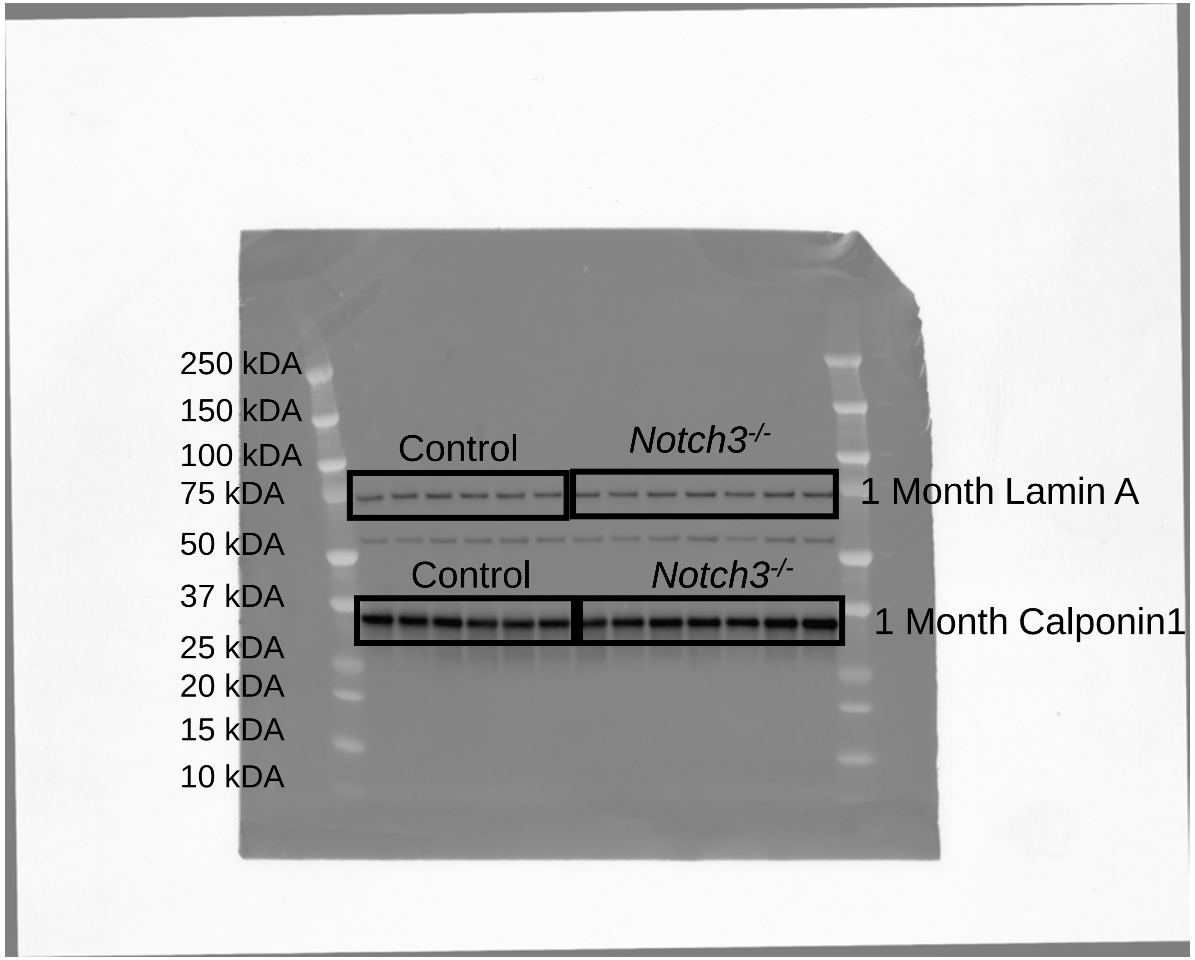

Supplement: Unedited blot and gel images [file jci-134-166134-s236.pdf]
